# Modeling the Mechanics of Cell Division: Influence of Spontaneous Membrane Curvature, Surface Tension, and Osmotic Pressure

**DOI:** 10.3389/fphys.2017.00312

**Published:** 2017-05-19

**Authors:** Elena Beltrán-Heredia, Víctor G. Almendro-Vedia, Francisco Monroy, Francisco J. Cao

**Affiliations:** ^1^Departamento de Física Atómica, Molecular y Nuclear, Universidad Complutense de MadridMadrid, Spain; ^2^Departamento de Química Física I, Universidad Complutense de MadridMadrid, Spain; ^3^Translational Biophysics, Instituto de Investigación Sanitaria Hospital 12 de Octubre (imas12)Madrid, Spain

**Keywords:** cell division, membrane constriction, bending energy, spontaneous curvature, surface tension, osmotic pressure, perturbative methods, analytical models

## Abstract

Many cell division processes have been conserved throughout evolution and are being revealed by studies on model organisms such as bacteria, yeasts, and protozoa. Cellular membrane constriction is one of these processes, observed almost universally during cell division. It happens similarly in all organisms through a mechanical pathway synchronized with the sequence of cytokinetic events in the cell interior. Arguably, such a mechanical process is mastered by the coordinated action of a constriction machinery fueled by biochemical energy in conjunction with the passive mechanics of the cellular membrane. Independently of the details of the constriction engine, the membrane component responds against deformation by minimizing the elastic energy at every constriction state following a pathway still unknown. In this paper, we address a theoretical study of the mechanics of membrane constriction in a simplified model that describes a homogeneous membrane vesicle in the regime where mechanical work due to osmotic pressure, surface tension, and bending energy are comparable. We develop a general method to find approximate analytical expressions for the main descriptors of a symmetrically constricted vesicle. Analytical solutions are obtained by combining a perturbative expansion for small deformations with a variational approach that was previously demonstrated valid at the reference state of an initially spherical vesicle at isotonic conditions. The analytic approximate results are compared with the exact solution obtained from numerical computations, getting a good agreement for all the computed quantities (energy, area, volume, constriction force). We analyze the effects of the spontaneous curvature, the surface tension and the osmotic pressure in these quantities, focusing especially on the constriction force. The more favorable conditions for vesicle constriction are determined, obtaining that smaller constriction forces are required for positive spontaneous curvatures, low or negative membrane tension and hypertonic media. Conditions for spontaneous constriction at a given constriction force are also determined. The implications of these results for biological cell division are discussed. This work contributes to a better quantitative understanding of the mechanical pathway of cellular division, and could assist the design of artificial divisomes in vesicle-based self-actuated microsystems obtained from synthetic biology approaches.

## Introduction

**Figure 1:**
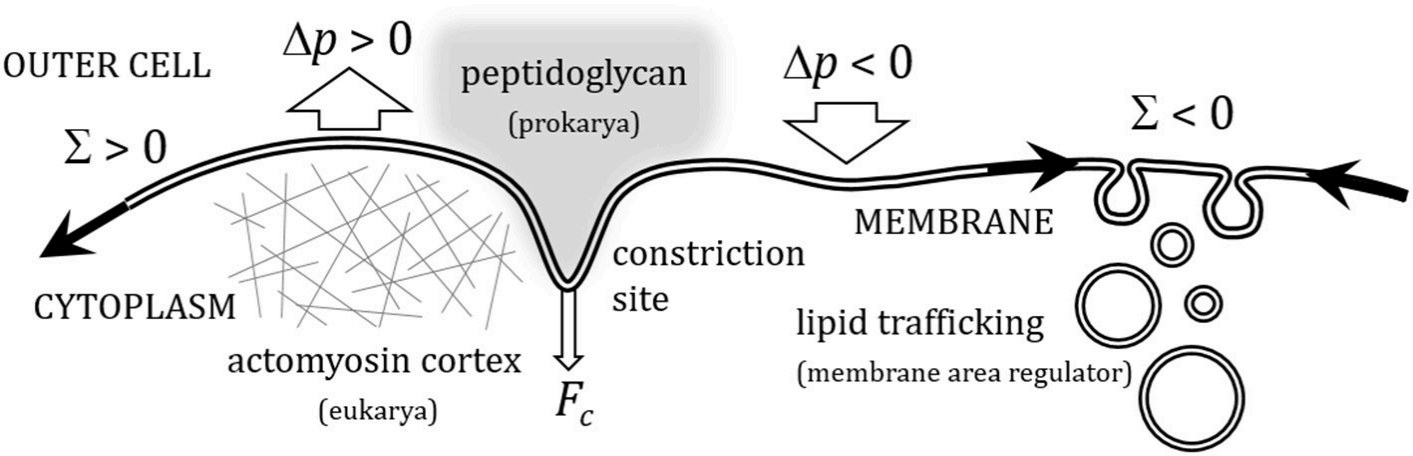
**Sketch depiction of the different modes of deformation possible in a flexible membrane under the action of a constriction force (F_c_) (representative of the constriction deformations in cellular plasma membranes at the site of cell division), and of external stress fields applied either transversally, as a hydrostatic osmotic pressure (Δp), or longitudinally, as a lateral membrane tension (Σ)**. Positive osmotic pressure (Δ*p* > 0), represents a cell at the inflated state of turgor, whereas negative osmotic pressure (Δ*p* < 0) is identified with a flaccid cellular membrane in a hypertonic medium. Regarding lateral membrane tensions, positive surface tension (Σ > 0) represents biological situations of membrane tension underlateral extensional stresses induced by cortical tensions induced by either the eukaryote cytoskeleton, or the peptidoglycan layer in bacteria; negative surface tension (Σ < 0) represents situations of regulated creation of membrane area under *in situ* membrane biogenesis, or membrane uptake from membrane shuttles coming from the metabolic route of lipid synthesis (lipid trafficking).

The cell division cycle is a central process in biology, the essential mechanism whereby cells grow and duplicate (Carlson, 2007). The mechanics of cell division is an essential part of the epigenetic program that supports cellular reproduction in all living organisms (Boal, 2012). The division program of any cellular organism involves changes in cell shape that are directly determined by the intrinsic deformability of the cellular plasma membrane. Far from being a passive element, the mechanics of the cellular plasma membrane is known to be physically, as well as biochemically, influenced by different transport processes, particularly, membrane biogenesis shuttled by lipid trafficking from the sites of metabolic synthesis to the cellular membranes (Blom et al., 2011), and stress-induced membrane remodeling occurred under the action of the cytokinetic machinery which, together with other passive skeletal structures, form the cellular divisome. Cytokinetic machinery is different in prokaryotes (Bi and Lutkenhaus, 1991; Romberg and Levin, 2003; Dajkovic and Lutkenhaus, 2006; Lan et al., 2007) and eukaryotes (Weiss, 1961; Cao and Wang, 1990; Rappoport, 1996; Alberts et al., 2007; Carlson, 2007; Lecuit and Lenne, 2007), but both provide mechanisms to generate constriction forces. Cytokinetic membrane remodeling is assumed to arise from a mechanical interplay between membrane tension, osmotic stresses and constriction forces exerted by the divisome. These membrane stresses underlie subcellular force effectors, which are structurally and functionally coupled to dynamically adaptable plasma membrane, the extracellular medium and the cytoskeleton (Lecuit and Lenne, 2007). In prokaryote division, the constricted cellular membrane is maintained under tension by the resistance of an outer peptidoglycan layer, which is dynamically linked to the inner lipid membrane (Koch et al., 1981; Huang et al., 2008; Bisson-Filho et al., 2017). In eukaryotes, however, cortical tensions generated under actomyosin contraction are assumed to be the main source of membrane tension during cytokinesis (Manning et al., 2010; Stewart et al., 2011). Secondarily, membrane trafficking may have the effect of buffering membrane tension by varying cell membrane surface (Sens and Turner, 2006). A quantitative insight on the membrane configurations that minimize the mechanical energy during cytokinesis is an important topic in cell biophysics (Lipowsky, 1991; Boal, 2012). Such membrane-focused rationale should allow us to compute the forces needed to divide the cell, thus providing a better understanding about the different routes of cell division in different organisms (Szostak et al., 2001; Chen, 2009; Budin and Szostak, 2011). Cell growth and further division requires indeed de novo synthesis of plasma membrane (Alberts et al., 2007). All cells can synthesize lipid molecules that are dynamically incorporated into their membranes (Morré, 1975; Nohturfft and Zhang, 2009). Biosynthetic lipid transport ensures that each cellular membrane have dynamically regulated an adequate lipid composition, which supports the functions of the associated proteins (Alberts et al., 2007). Cells have developed several, often redundant, mechanisms to transport lipids during the different stages of the cell cycle (Jackowski, 1996; McCusker and Kellog, 2012; Sanchez-Alvarez et al., 2015), which synchronize with the membrane growth occurred during cytokinetic progression (Dobbelaere and Barral, 2004; Albertson et al., 2005; Boucrot and Kirchhausen, 2007). In this article, we provide a minimal physical model for membrane constriction that considers either, impeded growth of membrane area characterized by positive membrane tension, which requests mechanical work to be exerted by the cytokinetic machinery (Lan et al., 2007; Lecuit and Lenne, 2007), or facilitated membrane growth characterized by negative membrane tension. Figure 1 depicts the possible modes of deformation of a model (lipid bilayer) membrane under stresses induced by constriction forces, and external fields with different orientations. A great amount of energy, which ultimately depends on cell size and membrane rigidity, is needed to distort the unconstricted initial configuration of the deformable membrane. The knowledge of these energies is especially interesting to know how the cell performs the large curvature deformations required for membrane constriction at the site of division.

In the present paper, the natural cell is depicted as a vesicle compartment enclosed by a lipid bilayer membrane in which a given constriction force is applied to create a circumferential furrow positioned at the cell equator. Different methods are available to obtain the minimum energy shape of a membrane under given constrains and boundary conditions. They are based on calculating the membrane bending energy with the Canham-Helfrich Hamiltonian (Canham, 1970; Helfrich, 1973) and minimizing it through numerical procedures (Seifert and Lipowsky, 1995; Jülicher and Lipowsky, 1996), perturbation methods (Höger et al., 2010; Almendro-Vedia et al., 2015), or variational approaches (Almendro-Vedia et al., 2013). In a previous paper (Almendro-Vedia et al., 2015), we combined a perturbation expansion for small deformations with a variational approach to compute the minimum energy shapes during the symmetrical constriction of a tensionless vesicle. Here, using a similar framework, we derive analytical formulas during constriction under more general conditions, which account of the additional effects of non-zero spontaneous curvature, membrane tension, and osmotic pressure. Additionally, exact results are computed numerically by solving the corresponding Euler-Lagrange equations (see Section 1 of Supplementary Material). This let us determine the accuracy of the approximate analytical results. By expanding the quantities up to sixth-order of perturbation in the deformations around the non-constricted shape, a good agreement between analytic and exact results is reached for low and intermediate constriction stages. Once the shape that minimizes the energy was calculated, other relevant properties of the system were obtained. Therefore, the proposed method should be sufficiently powerful to map the energy landscape of several mechanical pathways required for optimal cell division in a wide variety of biological situations.

This paper is organized as follows: in Section Method, we present the model used to compute the mechanical energy of an axisymmetric vesicle. In Section Approximate Analytical Expressions, we derive the analytical approximate formulas for the main properties of the constricted vesicle up to sixth-order of perturbation. In the next subsections, these formulas are compared with the (exact) solution of the Euler-Lagrange equations computed numerically following the procedure explained in Section 1 of Supplementary Material (SM). In Section Osmotic Pressure and Surface Tension Effects with No Spontaneous Curvature, we show the effects of the surface tension and osmotic pressure in the case of zero spontaneous curvature, in Section Spontaneous Curvature Effects we analyze the effects of the spontaneous curvature, focusing on its impact on the constriction force, especially at the onset of spontaneous constriction and, in Section Constant area and Constant Volume Conditions, we show how to extend the model for constant area and constant volume conditions. In Section Discussion we discuss the main results in the context of the relevant biological situations and finally, in Section Conclusions, we expose our conclusions.

## Methods

### Simplified mechanical model for cells and vesicles

As previously stated, the natural cell is depicted as a vesicle compartment enclosed by a lipid bilayer membrane in which a given constriction force is applied to create a circumferential furrow positioned at the cell equator. The cellular membrane is characterized by bending rigidity, spontaneous curvature and surface tension. The turgor of the vesicle is maintained under a positive difference of osmotic pressure between the inside cell and the outside extracellular milieu, which represents hypotonic conditions. Flaccid configurations are defined, in general by iso-, hypertonic conditions characterized by zero, or negative, osmotic pressure. Whereas positive membrane tension represents tensioned membrane vesicles forced to create area at the expenses of delivering work of dilation, negative membrane tension will be allowed to consider flaccid vesicles under continuous membrane biogenesis. The problem will be considered in the regime where mechanical work due to osmotic pressure, surface tension, and bending energy are comparable. We extend here the technique presented in Almendro-Vedia et al. (2015), which combines a perturbation expansion for small deformations with a variational approach to compute the minimum energy shapes during the symmetrical constriction of a tensionless vesicle. In such reference problem, a flaccid vesicle was assumed to be constricted at isotonic conditions, and to have a homogeneous membrane with zero spontaneous curvature and negligible tension. In that case, the initial configuration was a spherical vesicle.

**Figure 2:**
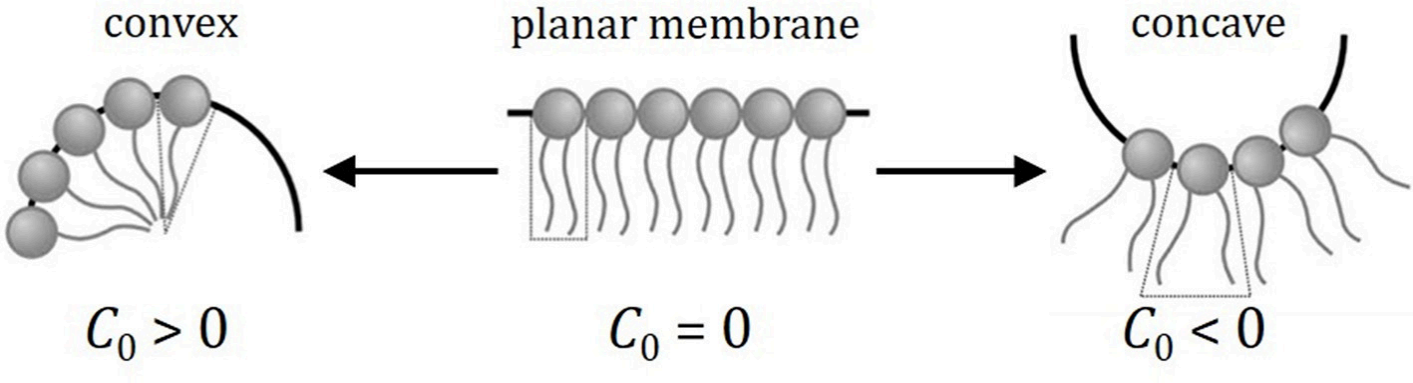
**Cartoon illustrating how local membrane curvature is determined by the molecular structure of the constituting lipids**. Usual phospholipids with a cylindrical molecular aspect assemble as planar membrane aggregates (only a monolayer is shown). In this case (central panel), the equilibrium configuration essentially corresponds to flat bilayer with a zero spontaneous curvature. Charged phospholipids, or lysed species with only one acyl chain present, which show an inverted-cone molecular aspect, cause the membrane to spontaneously bend in a convex configuration (left panel). Inclusion of these membrane molecular formers with a bigger polar head than the thin hydrophobic counterpart leads to situations with positive values of local spontaneous curvature (*C*_0_ > 0). Conversely, cone-like phospholipids (right panel), with a big hydrophobic counterpart thicker than the polar head, leads to membrane aggregates with a concave configuration, which represents an equilibrium bending characterized by a negative spontaneous curvature (*C*_0_ < 0).

The constriction region was described with approximate solutions based on trigonometric functions, whose local curvature is allowed to change depending on the constriction stage. We found previously in Almendro-Vedia et al. (2013) that such ansatz accurately reproduced the results of numerical computations in a broad range of constriction stages. When the spontaneous curvature, surface tension, and osmotic pressure are non-zero, the initial equilibrium configuration of the vesicle (or the cell) is, in general, non-spherical, but can be approximately represented by an ellipsoid, which can be oblate, prolate or spherical in function of the specific values of these parameters. This represents a more general physical scenario and lets us analyze the effect of the spontaneous curvature, the surface tension and the osmotic pressure on vesicle constriction and explain in more detail the biological and physical meaning of these quantities. Spontaneous curvature, *C*_0_, describes membranes with possible asymmetries in the two lipid monolayers resulting in a convex (*C*_0_ > 0), flat (*C*_0_ = 0), or concave (*C*_0_ < 0) membrane at mechanical equilibrium (see Figure 2). The symmetrical case results in a flat membrane at mechanical equilibrium (*C*_0_ = 0), which was the case discussed in Almendro-Vedia et al. (2015). Surface tension, Σ, is defined as the mechanical work per unit area required to increase the membrane area (Σ > 0). It allows to describe different extensional states of biological membranes as the key regulator of cell surface mechanics (Booth et al., 2007; Lecuit and Lenne, 2007). The differential pressure between inside and outside the vesicle, Δ*p*, usually realized as an osmotic pressure, gives the work per unit volume to increase the vesicle volume and allows describing different turgor states of the constricted vesicles, or cells, either turgid (Δ*p* > 0), or flaccid (Δ*p* ≤ 0). Specifically, we consider analytic solutions to the general problem of a constricted vesicle constrained by non-zero values of spontaneous curvature, osmotic pressure, and membrane tension, in the regime where these effects and bending energy are comparable, this is when *C*_0_*R*_*m*_ ≈ 1, and $\Delta pR_{m}^{2}\approx \Sigma R_{m}^{2}\;\approx \;\kappa {\left(1-C_{0}R_{m}\right)}^{2}$ (*R*_*m*_ being the vesicle radius, and κ the bending modulus).

### Elastic energy of a membrane vesicle: bending hamiltonian and total energy under non-zero osmotic pressure and non-zero surface tension

The membrane of a vesicle, or of a living cell, is composed of a lipid bilayer with a thickness that is much smaller than the dimensions of the vesicle. Therefore, the lipid bilayer can be represented approximately by a two-dimensional mathematical surface in the context of the mechanics of the whole cell. In 1973, Helfrich (1973), proposed a simple expression for the bending energy of a membrane in terms of the contributions from mean curvature *H* (first term, *E*_*m*_) and Gaussian curvature *K* (second term, *E*_*G*_), which are the two geometrical invariants that define the local curvature of the membrane:

(1) $$E_{b}\;=\;E_{m}\;+\;E_{G}=\frac{\kappa }{2}\int_{\Omega }{\left(2H-C_{0}\right)}^{2}dA\;+\;\kappa_{G}\int_{\Omega }KdA.$$

Here, Ω is the closed surface that defines the membrane vesicle, and *dA* its element of area. The parameters κ and κ_*G*_ are the bending modulus and the Gaussian bending rigidity, respectively. The spontaneous curvature, *C*_0_, permits to describe bilayers that are spontaneously curved in their equilibrium state due to the compositional inhomogeneity between the inner and the outer monolayers. This term represents the spontaneous tendency of the membrane to build up in a concave (as *C*_0_ < 0), convex (as *C*_0_ > 0), or flat (as *C*_0_ = 0) surface (see Figure 2). In this work, we assume that *C*_0_ is uniform over the vesicle.

In terms of the local principal curvatures of the membrane surface, *C*_1_ and *C*_2_, we have *H* = (*C*_1_ + *C*_2_)/2 and *K* = *C*_1_*C*_2_, and the bending energy of the vesicle takes the form:

(2) $$E_{b}=\frac{\kappa }{2}\int_{\Omega }{\left(C_{1}\;+\;C_{2}\;-\;C_{0}\right)}^{2}dA\;+\;\kappa_{G}\int_{\Omega }C_{1}C_{2}dA.$$

For a spherical shell of radius *R*_0_, *C*_1_ = *C*_2_ = 1/*R*_0_, the bending energies are $E_{m}^{\left(\text{sph}\right)}\;=\;8\pi \kappa {\left(1-R_{0}C_{0}/2\right)}^{2}$ and $E_{G}^{\left(\text{sph}\right)}\;=\;4\pi \kappa_{G}$ for the mean and Gaussian contributions, respectively. A non-zero value for the spontaneous curvature has strong effects on the configuration of the spherical shell. First, it introduces a characteristic length scale $l_{c}\approx \;C_{0}^{-1}$, differently to the case of zero spontaneous curvature for which the deformation energy is a size invariant, this is *E*_*b*_(*C*_0_ = 0) = 8πκ + 4πκ_*G*_. Since the bending energy of a spherical shell with *C*_0_ ≠ 0 is dependent on *R*_0_ as $E_{b}\left(R_{0};C_{0}\right)\;=\;8\pi \kappa {\left(1-R_{0}C_{0}/2\right)}^{2}\;+\;4\pi \kappa_{G}$, it minimizes at a radius $R_{0}=R_{\text{min}}\;=\;2C_{0}^{-1}$, with the evident consequence that the spherical shell with the lowest bending energy $E_{b}^{\left(\text{min}\right)}\left(R_{\text{min}}\right)\;=\;4\pi \kappa_{G}$ corresponds to the particular size $R_{\text{min}}=2C_{0}^{-1}$ at *C*_0_ ≠ 0. This conclusion is true for arbitrary shapes (Boal, 2012), meaning that the bending energy is a function not only of cell shape but also of cell size at *C*_0_ ≠ 0. In addition, the sign of *C*_0_ influences the favored shape of the deformed vesicle (Boal, 2012); predominantly convex pear-like shapes are preferred if *C*_0_ > 0, and predominantly concave shapes are favored if *C*_0_ < 0 (Figure 2).

Under the osmotic pressure Δ*p* (the inner minus the outer pressure; Δ*p* > 0 for an inflated vesicle) and under the action of the surface tension Σ, the total energy of a vesicle is given by:

(3) $$E_{T}\;=\;E_{b}\;+\;\int_{\Omega }\Sigma dA\;+\;\Delta p\int_{V}dV,$$

where *dV* is the element of volume enclosed by the vesicle. Here, we assume that Σ is uniform along the membrane surface and Δ*p* isotropic. Consequently, we can express the Equation (3) as:

(4) $$E_{T}\;=\;E_{b}\;+\;\Sigma \text{A }+\;\Delta pV.$$

Obviously, changing the membrane shape from its equilibrium configuration changes the total energy (Equation 4). However, the Gauss-Bonnet theorem^1^, shows that the integral over the Gaussian curvature, the second term in Equations (1) and (2), is constant for surfaces with the same topology. Since the constriction process in a vesicle does not change its topology, and only involves shapes that are topologically equivalent to a sphere (no holes), the contribution of *E*_*G*_ can be ignored because it remains constant, with $E_{G}\;=\;E_{G}^{\left(\text{sph}\right)}=4\pi \kappa_{G}$, independently of the size and shape of the vesicle. For the final state, in which the vesicle splits into two separated daughters, it is required to consider the Gaussian contribution since the topological change to two spheres requires an increase of curvature energy by Δ*E*_*G*_ = 4πκ_*G*_. Therefore, during the constriction process (before the final fission), we only analyze the variations of energy due to the mean curvature *E*_*m*_ and the effects of osmotic pressure and surface tension.

**Figure 3:**
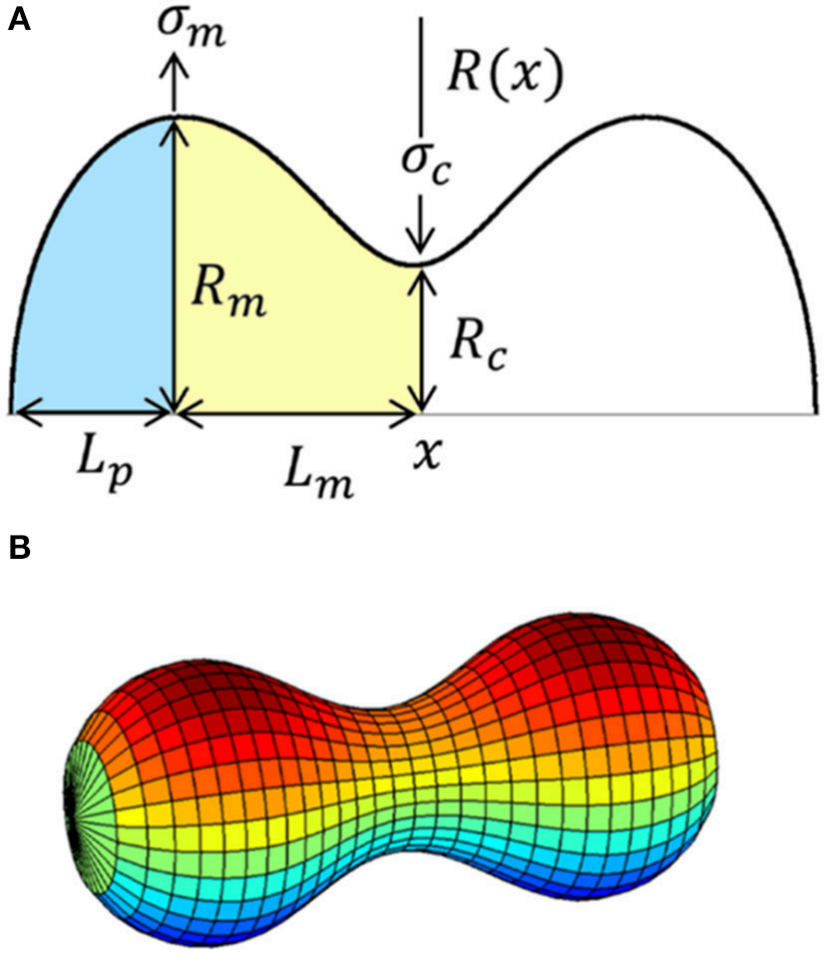
**(A)** Profile *R*(*x*) of a symmetrically constricted vesicle with the axis of symmetry along the *x*–axis and its characteristic parameters. Left polar cap is shaded in yellow and the left half of the constriction zone is shaded in blue. **(B)** Surface obtained from the revolution around the *x*–axis of the previous profile *R*(*x*).

We consider the particular case of axisymmetric shapes with the axis of symmetry along the *x*–axis. When these shapes are represented in Cartesian coordinates as $\vec{r}=\left(x,y,h\left(x,y\right)\right)$ with *h*(*x, y*), the surface profile can be given as a height on the *x* − *y* plane:

(5) $$h(x,y)\;=\;\pm \sqrt{R^{2}(x)\;-\;y^{2}},$$

where *R*(*x*) is the functional form describing the membrane profile in the *x* − *z* plane (see Figure 3A). If the membrane surface is located between *x*_*i*_ and *x*_*f*_, its bending energy is given by Boal (2012):

(6) $$E_{m}\;=\;\pi \kappa \int_{x_{i}}^{x_{f}}K_{m}(x)dx,$$

with the kernel

(7) $$K_{m}(x)\;=\;\frac{{\left[1\;+\;R_{x}^{2}\;-\;R_{xx}R\;-\;RC_{0}{\left(1\;+\;R_{x}^{2}\right)}^{3/2}\right]}^{2}}{R{\left(1+R_{x}^{2}\right)}^{5/2}},$$

where *R*_*x*_ = ∂*R*/∂*x* and *R*_*xx*_ = ∂*R*_*x*_/∂*x* are, respectively, the first and second derivatives of the membrane profile *R*(*x*). Furthermore, other relevant vesicle properties can also be computed, particularly the membrane area and the volume enclosed. For a given profile shape *R*(*x*), the area of the corresponding revolution surface around the *x*–axis is:

(8) $$A\;=\;2\pi \int_{x_{i}}^{x_{f}}R\sqrt{1+R_{x}^{2}}dx,$$

and the volume enclosed by this surface is:

(9) $$V\;=\;\pi \int_{x_{i}}^{x_{f}}R^{2}dx.$$

Note that Equations (7)–(9) are independent of the coordinate *y*, as expected for surfaces with rotational symmetry around *x*. Along the constriction pathway, the vesicle will take the shapes that minimize the total energy *E*_*T*_ (up to thermal effects). In particular, *E*_*T*_ must be stationary under an infinitesimal scale transformation $\vec{r}\to \lambda \vec{r}$ with small λ − 1. This leads to the following transformations (Seifert and Lipowsky, 1995), κ → κ, *C*_0_ → *C*_0_/λ, *C*_1_ → *C*_1_/λ, *C*_2_ → *C*_2_/λ, *A* → λ^2^*A*, *V* → λ^3^*V*, Σ → Σ/λ^2^, and Δ*p* → Δ*p*/λ^3^. This means that the shape that minimizes the energy with *C*_0_, Σ, and Δ*p*, has the same energy (and also minimize the energy) under an overall dilatation $\vec{r}\to \lambda \vec{r}$ with *C*_0_ → *C*_0_/λ, Σ → Σ/λ^2^, and Δ*p* → Δ*p*/λ^3^. Note that when *C*_0_ = Σ = Δ*p* = 0, the total energy of the vesicle, which is equal to the bending energy, becomes size invariant. This particular case was previously studied by us (Almendro-Vedia et al., 2013, 2015). Here, we consider the more general case, where *C*_0_, Σ, and Δ*p* are non-zero, and analyze the effects of these parameters for the more relevant properties of the system.

### Perturbation method

We consider the constriction process of a membrane vesicle with rotational symmetry around the longitudinal axis and with central symmetry. The break of the central symmetry can be treated as a stability problem against a linear perturbation from the symmetric case (Almendro-Vedia et al., 2013, 2015). The initial vesicle deforms by the action of a radial tension exerted as a constriction ring at its equator, which decreases the equator radius till formation of a saddled neck that becomes thinner and thinner under the action of the constriction force (see Figure 3). These processes will be followed by the vesicle splitting into two separated daughter vesicles. In previous papers (Almendro-Vedia et al., 2013, 2015), we restricted the study to the case of zero spontaneous curvature *C*_0_ = 0, negligible tension Σ = 0, and no pressure difference between internal and external media Δ*p* = 0, in which the total energy of the vesicle corresponds exclusively to the bending energy (up to thermal effects). In that particular case, the unconstricted initial configuration is a sphere of radius *R*_*m*_ and the constriction process is assumed to proceed by keeping this maximum radius *R*_*m*_ constant, which is equivalent to consider that the two polar caps are hemispheres of radius *R*_*m*_ during the whole process. In the present case, as the parameters *C*_0_, Σ, and Δ*p* are non-zero, the initial configuration is not, in general, a sphere of radius *R*_*m*_, but a spheroid with polar radius *R*_*m*_ (distance from the center to the upper pole of the spheroid). This spheroid can be an oblate spheroid (when the polar distance *L*_*p*_, see Figure 3A, is smaller than *R*_*m*_), a prolate spheroid (when the polar distance *L*_*p*_ is greater than *R*_*m*_) or a sphere (when the polar distance *L*_*p*_ is equal to *R*_*m*_). The value of the dimensionless ratio *L*_*p*_/*R*_*m*_ will depend on the particular values of the dilatation invariant products *C*_0_*R*_*m*_, $\Sigma R_{m}^{2}$, and $\Delta pR_{m}^{3}$. As in Almendro-Vedia et al. (2013, 2015) the constriction is assumed to proceed by keeping the polar radius *R*_*m*_ constant, which implies that, by fixing constant *C*_0_, Σ, and Δ*p*, the shape of the polar caps remains equal to the initial configuration at all stages of constriction. Consequently, the total energy of the polar caps does not change during constriction, making all energy variations arise from central constriction region that goes from *R*_*c*_ = *R*_*m*_ to *R*_*c*_ = 0 (see Figure 3). The case of constant *R*_*m*_ may describe cells whose structure or contents (cytoskeleton, peptidoglycan wall, nucleoid exclusion) exert an effective line tension at the maximum radius sites toward the exterior, Σ_*m*_. Similarly, the force needed to constrain up to a radius *R*_*c*_ is delivered by an effective line tension Σ_*c*_ around all the constriction ring and directed toward the cell interior (see Figure 3A). Each constriction state can be characterized by a constriction parameter *s*, which is defined in terms of the ratio between the constriction radius *R*_*c*_ and the polar radius *R*_*m*_ in the form:

(10) $$s\;=\;1\;-\;R_{c}/R_{m}.$$

**Figure 4:**
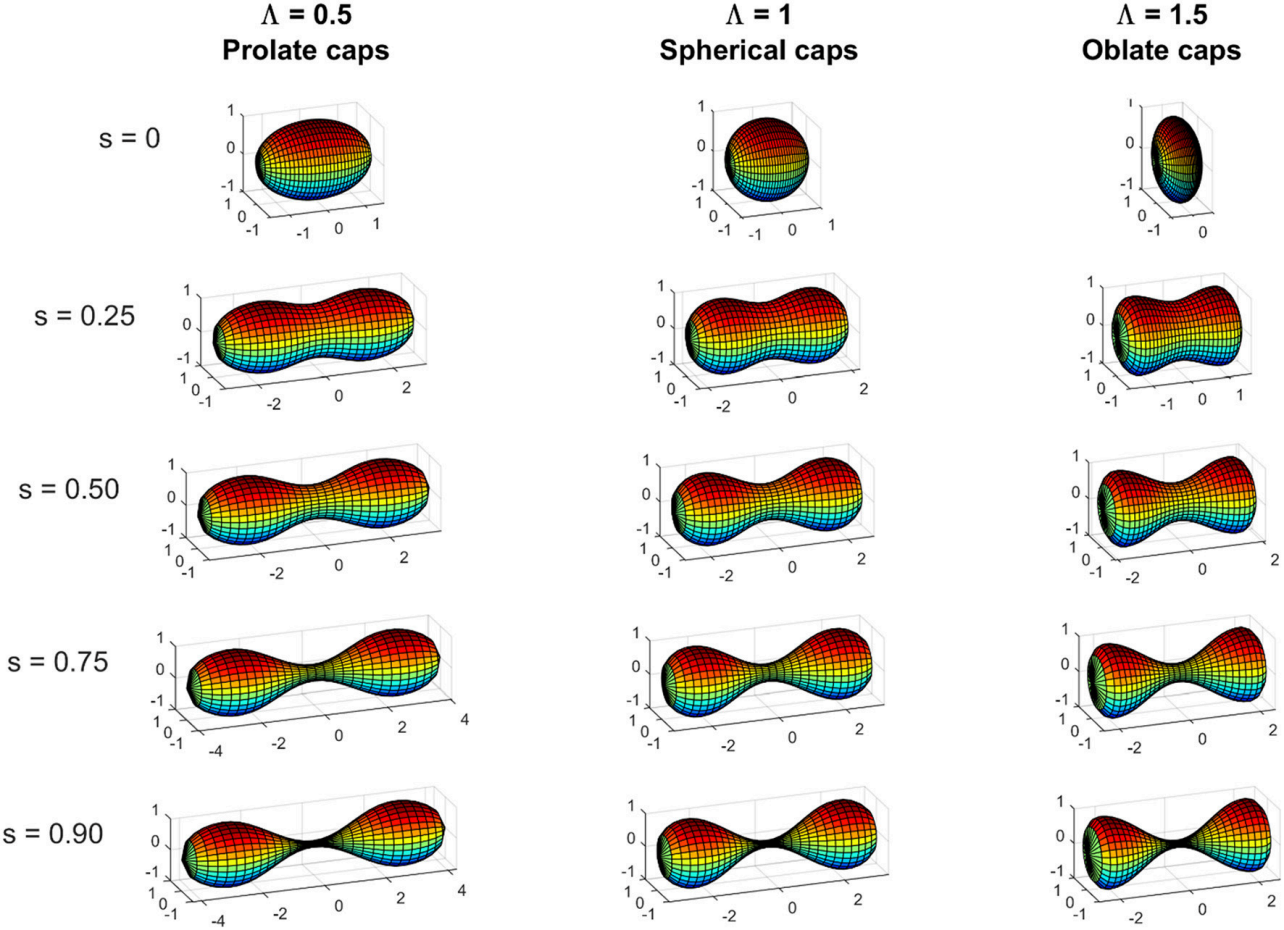
**Shapes at various constriction stages characterized by the constriction parameter ***s*** (Equation 10) (***s*** = 0; ***s*** = 0.25; ***s*** = 0.5; ***s*** = 0.75; and ***s*** = 0.9) for initially unconstricted prolate, spherical, and oblate vesicles**. Prolate case (*L*_*p*_ = 1.5*R*_*m*_) with Λ = 0.5, spherical case (*L*_*p*_ = *R*_*m*_) with Λ = 1, and oblate case (*L*_*p*_ = 0.5*R*_*m*_) with Λ = 1.5, and Γ = −24 for the three cases represented (see Equations 24 and 25) for the definitions of Λ and Γ in terms of *C*_0_*R*_*m*_, $\tilde{\Sigma }R_{m}^{2}$ and ($\Delta \tilde{p}R_{m}^{3}$). When Γ > −29: Λ < 1 gives prolate polar caps (i.e., *L*_*p*_ > *R*_*m*_), Λ = 1 gives spherical polar caps (i.e., *L*_*p*_ = *R*_*m*_), and Λ > 1 gives oblate polar caps (i.e., *L*_*p*_<*R*_*m*_); while when Γ < −29 the opposite relation between the values of Λ and the shape of the polar holds.

This parameter increases from *s* = 0 when there is no constriction and *R*_*c*_ = *R*_*m*_ to *s* = 1 when the constriction is maximal and *R*_*c*_ = 0 (see Figure 4).

Once the origin of the *x* coordinate is established in the middle point of the vesicle (see Figure 3), the constriction profile is given by:

(11) $$R\left(x;s\right)=\{\begin{array}{lll} R_{\text{left polar cap}}(x) & \text{if} & x\;\in \;[-L_{p}\;-\;L_{m},\;-L_{m}]\\ R_{\text{cz}}(x;s) & \text{if} & x\;\in \;[-L_{m},L_{m}]\\ R_{\text{right polar cap}}(x) & \text{if} & x\;\in \;[L_{m},\;L_{m}\;+\;L_{p}] \end{array}$$

where *L*_*m*_ and *L*_*p*_ represent the half of the length of the constriction zone and the polar distance, respectively (see Figure 3A). Note that the constriction profile must be continuous in the boundaries of the zones. In addition, since the shape has central symmetry we have the relation *R*_left pole cap_(*x*) = *R*_right pole cap_(−*x*).

The perturbative method allows us to easily obtain approximate analytical formulas in terms of *C*_0_, Σ, Δ*p* and the scaling parameter *R*_*m*_. We need to determine the shapes that minimize the total energy of the vesicle along the constriction pathway, i.e., for each constriction stage *s* between 0 and 1. In order to determine the approximate shape we assume an appropriate ansatz for *R*(*x*) in each of the existence intervals. These ansatzs will be expressed in terms of the characteristic length rates of each zone: *L*_*p*_/*R*_*m*_ for the polar caps and *L*_*m*_/*R*_*m*_ and *s* for the constriction zone (recall that the polar caps remain constant independently of *s*). The constriction profile *R*(*x*), together with its first and second derivatives, *R*_*x*_ = ∂*R*/∂*x* and *R*_*xx*_ = ∂*R*_*x*_/∂*x*, allow us computing the integrand *K*_*m*_ (Equation 7) in each zone. Recall that we are assuming that *C*_0_ and Σ are uniform along the whole surface. In order to apply the perturbative method it is convenient to define a small deformation function and expand *K*_*m*_ in power series of it and of its first- and second-order derivatives. Then, introducing this simplified integrand in the total energy (Equation 6), we can perform the integration between the two boundaries that define the corresponding surface interval (Equation 11). Finally, the total energy minimization with respect to the characteristic length of each zone provides their optimal values:

(12a) $$\frac{\partial E_{T,\text{ polar caps}}\left(L_{p},R_{m},C_{0},\Sigma ,\Delta p,\kappa \right)}{\partial L_{p}}\;=\;0\;\overset{\text{yields}}{\to }L_{p}^{\text{opt}},$$

(12b) $${\frac{\partial E_{T,\text{ cz}}(s,\;L_{m},\;R_{m},C_{0},\;\Sigma ,\;\Delta p,\kappa )}{\partial L_{m}}|}_{s}=\;0\;\overset{\text{yields}}{\to }\;L_{m}^{\text{opt}}(s).\;$$

As the polar caps do not change their shape during the whole constriction process, the polar distance *L*_*p*_ (Equation 12a) is independent of the constriction parameter *s*, (and therefore, the other properties of the system calculated on the polar caps zone are independent of *s* too). However, the length of the constriction zone changes with the constriction parameter *s*. Once the optimal total length ${L_{p}^{\text{opt}}+L}_{m}^{\text{opt}}\left(s\right)$ is obtained, it is possible to determinate approximate analytical expressions for the more relevant properties of the system as are the total energy, the membrane area, the volume enclosed and the constriction force at any stage of constriction. These quantities will have the form of a series expansion in powers of the constriction parameter, *s*. In general, we found that, as expected, that the higher the order included, the better the predictions obtained.

### Shape of the polar caps zone: area and volume

The polar caps of a tensionless vesicle (Σ = 0), without pressure difference (Δ*p* = 0) and for zero spontaneous curvature (*C*_0_ = 0) with maximum radius *R*_*m*_ fixed constant, remain as hemispheres of radius *R*_*m*_ during the whole constriction process (Almendro-Vedia et al., 2013, 2015). However, if these parameters (Σ, Δ*p*, and *C*_0_) are not zero, we have to consider a more general profile for the polar caps. We consider here an ellipsoid with semi-axis *R*_*m*_ (polar radius) and *L*_*p*_ (polar distance) centered in *x* = *L*_*m*_ (see Figure 3A) with rotational symmetry around *x*-axis given by:

(13) $$R_{\text{right polar cap}}\;(x)\;=\;\pm \;R_{m}\sqrt{1-{\left(\frac{x-L_{m}}{L_{p}}\right)}^{2}},$$

with *x*∈ [*L*_*m*_, *L*_*m*_+*L*_*p*_]. Now, in order to apply the perturbative method, we define the small deformation function in the polar caps zone as (see Figure 3):

(14) $$\varepsilon \;=\;\frac{L_{p}-R_{m}}{R_{m}},$$

which leads to:

(15) $$L_{p}\;=\;R_{m}(1+\varepsilon ).$$

The global sign of ε determines the shape of the polar caps. When ε is negative, positive, or zero, the polar caps are oblate, prolate, or spherical, respectively.

Once we have calculated the length *L*_*p*_, other relevant magnitudes can be obtained, particularly the membrane are of the polar caps and the volume enclosed on them. Using the expressions for a surface of revolution (Equations 8 and 9) with the profile of the Equation (13), and integrating between the limits *x*_*i*_ = *L*_*m*_ and *x*_*f*_ = *L*_*m*_ + *L*_*p*_ (see Figure 3A) we obtain for the membrane area:

(16) $$A_{\text{polar caps}}/A_{\text{sph}}\;=\;1\;+\;\frac{2}{3}\varepsilon \;+\;\frac{1}{15}\varepsilon^{2}\;+\;\ldots ,$$

where $A_{\text{sph}}\;=\;4\pi R_{m}^{2}$, and for the volume enclosed:

(17) $$V_{\text{polar caps}}/V_{\text{sph}}\;=\;1\;+\;\varepsilon \;=\;L_{p}/R_{m},$$

where $V_{\text{sph}}=4/3\pi R_{m}^{3}$. Note that the (Equation 16) can be also obtained by expanding the surface area of the spheroid around ε = 0, $A_{\text{spheroid}}\;=\;2\pi R_{m}^{2}\;\left[1\;+\;L_{p}/\left(R_{m}e\right)\text{ArcSin}\left(e\right)\right]$ with $e^{2}\;=\;1-{\left(R_{m}/L_{p}\right)}^{2}\;=\;\varepsilon \left(\varepsilon \;+\;2\right)/{\left(1\;+\;\varepsilon \right)}^{2}$; and the (Equation 17) is exact and corresponds to the volume of the spheroid, $V_{\text{spheroid}}\;=\;4/3\pi R_{m}^{2}L_{p}$.

### Shape of the constriction zone: area and volume

In Almendro-Vedia et al. (2015), we used the variational approach to find the shape that minimize the energy for different constriction stages in the case of *C*_0_ = 0, Σ = 0 and Δ*p* = 0. There, we considered a family of solutions of the form:

(18) $$R(x;s)\;=\;R_{0}(x)\;+\;\sum_{i=1}^{\infty }a_{i}R_{i}(x)$$

in order to describe the constriction region, where the assumed zeroth-order function family was:

(19) $$R_{0}(x;s)\;=\;R_{m}\left\{1-\frac{s}{2}\left[1+\text{cos}\left(\frac{\pi x}{L_{m}}\right)\right]\right\}.$$

This simple zeroth-order provided good approximations for low and intermediate constriction regimes, as we previously saw in Almendro-Vedia et al. (2013). Consequently, we use here this term as the profile of the constriction region in order to apply the perturbative method to the general case. We define the small deformation function in the constriction region as:

(20) $$u(x;s)\;=\;R_{m}-R_{\text{cz}}(x;s)\;=\;\left(R_{m}/2\right)s\left[1+\text{cos}\left(\pi x/L_{m}\right)\right].$$

Introducing *R*_cz_(*x*; *s*) in terms of *u*(*x*; *s*) in the kernel of the total energy (Equation 7) and expanding it up to the fourth-order of perturbation in *u* (a higher-order expression can be found in Section 2 of SM) we obtain:

(21) $$\begin{array}{l} K_{T,\text{cz}}=\;\frac{1}{R_{m}}\;-2C_{0}\;+\;C_{0}^{2}R_{m}\;+\;\Delta \tilde{p}R_{m}^{2}\;+\;2R_{m}\tilde{\Sigma }\\ \;+\;\frac{1}{R_{m}^{2}}u\;-\;C_{0}^{2}u\;-\;2\Delta \tilde{p}R_{m}u\;-2\;\tilde{\Sigma }u\;+\;2uu_{xx}\\ \;-\;2\;C_{0}R_{m}u_{x}\;+\;\frac{1}{R_{m}^{3}}u^{2}\;+\;\Delta \tilde{p}u^{2}\;+2\;C_{0}uu_{xx}\;+\;R_{m}u_{xx}^{2}\\ \;-\;\frac{1}{2R_{m}}u_{x}^{2}\;+\;\tilde{\Sigma }R_{m}u_{x}^{2}\;+\;\frac{1}{R_{m}^{4}}u^{3}\;-3\;u_{x}^{2}u_{xx}\\ \;+\;2C_{0}R_{m}u_{x}^{2}u_{xx}-\;uu_{xx}^{2}\;-\;\frac{C_{0}^{2}}{2}uu_{x}^{2}\;-\;\frac{1}{2R_{m}^{2}}uu_{x}^{2}\\ \;-\tilde{\Sigma }uu_{x}^{2}\;+\;\frac{1}{R_{m}^{5}}u^{4}\;-2\;C_{0}uu_{x}^{2}u_{xx}\;-\;\frac{5R_{m}}{2}u_{x}^{2}u_{xx}^{2}\\ \;+\;\frac{3}{8R_{m}}u_{x}^{4}\;-\;\frac{1}{2R_{m}^{3}}u^{2}u_{x}^{2}\;-\;\frac{C_{0}^{2}R_{m}}{8}u_{x}^{4}\;+\;\ldots . \end{array}$$

As in the polar caps zone, once we know the dimensionless ratio *L*_*m*_/*R*_*m*_, we can determine other relevant vesicle properties, as are the membrane area of the constriction zone and the volume enclosed on it. Introducing the functional form *R*(*x*) in terms of the small-*u*(*x*) variable (Equation 20) in the formula of the membrane area (Equation 8) and expanding the integrand in a Taylor series up to fourth order in *u*, we obtain:

(22) $$\begin{array}{l} A_{\text{cz}}=\;2\pi \int_{x_{i}}^{x_{f}}R\sqrt{1\;+\;R_{x}^{2}}dx\;=\;2\pi \int_{x_{i}}^{x_{f}}[R_{m}\;-\;u\;+\frac{R_{m}}{2}u_{x}^{2}\\ \;-\;\frac{1}{2}uu_{x}^{2}\;-\;\frac{R_{m}}{8}u_{x}^{4}\text{+}\ldots ]dx. \end{array}$$

Similarly, expressing the integrand of the formula of the volume enclosed (Equation 9) in terms of the small variable *u*(*x*) (Equation 20) we obtain the exact result:

(23) $$\begin{array}{l} V_{\text{cz}}=\;\pi \int_{x_{i}}^{x_{f}}R^{2}dx\;=\;\pi \int_{x_{i}}^{x_{f}}{\left(R_{m}\;-\;u\right)}^{2}dx\\ \;=\pi \int_{x_{i}}^{x_{f}}\left(u^{2}\;-\;2R_{m}u\;+\;R_{m}^{2}\right)\;dx. \end{array}$$

### Exact numerical method: euler-lagrange equations

Analytical formulas derived with the perturbative method are compared with the (exact) solution of the Euler-Lagrange equations computed numerically. The Euler-Lagrange equations do not have an analytical solution in general, but can be solved numerically and different methods have been developed to solve them. As we previously made when we studied the case with *C*_0_ = Σ = Δ*p* = 0 in Almendro-Vedia et al. (2015), we use the methodology proposed in Jülicher and Lipowsky (1996) and Seifert and Lipowsky (1995), and apply it to axisymmetric shapes subject to equatorial constriction stress with polar radius *R*_*m*_ maintained constant (see Section 1 of SM for a brief explanation of the numerical procedure followed).

### Experimental values of bending and gaussian moduli

Experimental measurements of the bending modulus κ of lipid bilayers in the fluid state give values in the order of 10^−19^J, or 10−20 *k*_*B*_*T* at ambient temperatures (Marsh, 2006; Rodríguez-García et al., 2009; Boal, 2012; Nagle, 2013). They are obtained mostly either from analysis of thermally induced bending fluctuations, or more recently from pipette-aspiration techniques (Marsh, 2006). Observations of the phase behavior of lipid bilayers suggests that κ_*G*_ ≈ −0.8κ (Siegel and Kozlov, 2004; Marsh, 2006), which yields a Gaussian energy contribution to the energy of membrane fusion 4πκ_*G*_ in the order of 10^−18^J, or 100*k*_*B*_*T* at ambient temperatures (Mingyang et al., 2012). Recall that the Gaussian curvature energy is constant for surfaces with the same topology, independently of the size and shape of the surface. Only when fusion process happen, will the Gaussian energy contribution be considered.

## Results

The fundamental scales of the physical problem are determined both by the polar radius *R*_*m*_, which defines the spatial scale, and the bending rigidity κ, which defines the energy scale. In a scaling description, given a set of constitutive parameters (κ, *C*_0_, *R*_*m*_), the perturbation problem can be analytically solved for different values of the external fields (Σ, Δ*p*). The geometrical descriptors (volume, area, length, etc…) and the mechanical ones (energy, force, etc.) will be defined in terms of power series of two form parameters, the small parameter κ, which defines the shape aspect of the polar caps (see Equation 14), and the constriction parameter *s*, which defines the shape of the constriction region (see Equation 19). Furthermore, the two conditions for energy minimization (see Equation 12) establish additional constraints in the equilibrium problem, which are described by two independent linear relationships between constitutive properties and external fields; these are:

(24) $$\Lambda \;=\;{\left(1-C_{0}R_{m}\right)}^{2}\;+\;2\tilde{\Sigma }R_{m}^{2}\;+\;\Delta \tilde{p}R_{m}^{3},$$

(25) $$\Gamma =\;{\left(2-C_{0}R_{m}\right)}^{2}\;+\;2\tilde{\Sigma }R_{m}^{2}\;-1,$$

With:

(26) $$\tilde{\Sigma }=\frac{\Sigma }{\kappa },\;\Delta \tilde{p}=\frac{\Delta p}{\kappa }.$$

where Λ and Γ represent functional forms for the variations of the elastic Hamiltonian that minimize the energy of the vesicle for generalized geometry. In the particular case of constant Λ and Γ, these functional forms are linked to geometrical conditions that minimize the energy and constitute generalized Young-Laplace equations (Seifert et al., 1991; Zheng and Liu, 1993), which establish the equilibrium condition between the surface tension and the differential pressure for the different spheroidal geometries defined by the specific values of Λ and Γ. In particular, values of Λ ≠ 1 correspond to spheroids while Λ = 1 stands for the sphere. The meaning of Λ is more cumbersome, however since $\Gamma \;=\;\Lambda \;+\;2\left(1-C_{0}R_{m}\right)\;-\;\Delta \tilde{p}R_{m}^{3}$, it essentially refers to the inflation status of a given spheroidal shape. The exact numerical method depicted in Section Exact Numerical Method: Euler-Lagrange Equations, and further described in Section 1 of SM, allows for an accurate description of the constriction pathways of the different spheroids, whose initial surface area and volume are mutually linked for given values of the constitutive parameters {*C*_0_, Σ, Δ*p*} through Equations (24)–(26). A graphical summary of the main results is shown in Figure 4, which shows the constriction shapes of representative spheroids along the minimal energy pathway defined at constant Λ. In the following, the approximate solutions provided by perturbation method proposed in Sections Simplified Mechanical Model for Cells and Vesicles to Shape of the Constriction Zone: Area and Volume are compared with the exact solutions provided by the numerical analysis of the Euler-Lagrange equations (see Section Exact Numerical Method: Euler-Lagrange Equations).

### Approximate analytical expressions

In this subsection we show the approximate analytical expressions obtained for both polar caps and constriction zone in the regime where mechanical work due to osmotic pressure, surface tension, and bending energy are comparable, i.e. $\Delta p\;R_{m}^{3}\;\approx \;\Sigma R_{m}^{2}\;\approx \;\kappa {\left(1-C_{0}R_{m}\right)}^{2}$. A similar condition applies for the spontaneous curvature, which is restricted to the interval −1 ≤ *C*_0_*R*_*m*_ ≤ 1. We have derived the expressions of the characteristic length, the total energy, the membrane area, and the volume enclosed in both zones. These expressions are given in terms of the spontaneous curvature *C*_0_, the surface tension Σ, the osmotic pressure Δ*p*, and the maximum radius *R*_*m*_. In the constriction zone these expressions also depend on the constriction parameter *s* (recall that the polar caps remain constant independently of *s*). Finally, we have obtained the constriction force from the variations in the total energy during the constriction stage.

#### Polar caps zone

Introducing the expression of *L*_*p*_ (Equation 15) in Equation (13) and this, in turn, in Equation (7), we can integrate the resulting kernel between the limits *x*_*i*_ = *L*_*m*_ and *x*_*f*_ = *L*_*m*_ + *L*_*p*_ (see Figure 3A) and obtain an approximate analytical expression for the total energy of the right polar cap. As the left and the right polar caps are identical (due to the central symmetry assumed) it is enough to consider one of them and then multiply the expression by a factor 2. Up to second order of perturbation in ε, the total energy of the caps is:

(27) $$\begin{array}{l} E_{T,\text{polar caps}}=\;E_{\text{sph}}\;+\;\frac{4\pi \kappa }{3}(\Lambda \;-\;1)\varepsilon \\ \;+\;\frac{2\pi \kappa }{15}(\Gamma \;+\;29)\varepsilon^{2}\;+\;\ldots , \end{array}$$

where

(28) $$\begin{array}{l} E_{\text{sph}}=\;2\pi \kappa /3(2\Lambda \;+\;\Gamma \;+\;7-4C_{0}R_{m})\\ \;=\;\pi \kappa \left[8\;+\;2R_{m}^{2}C_{0}^{2}\;-\;8C_{0}R_{m}\;+\;4R_{m}^{2}\tilde{\Sigma }\;+\;4/3R_{m}^{3}\Delta \tilde{p}\right], \end{array}$$

Our analytical approximation is valid for small departure from the spherical shape of the polar caps, i.e., as long as |ε| ≪ 1. This implies that the quantities calculated in the polar caps zone are a slight modification of those corresponding to a sphere of radius *R*_*m*_. After integration, the total energy of the polar caps is minimized with respect to *L*_*p*_ (Equation 12a), obtaining the analytical expression for the optimal polar distance *L*_*p*_ of the caps. This length defines the shape of minimal energy and determines if the initial vesicle was an oblate spheroid (ε < 0), a prolate spheroid (ε > 0), or a sphere (ε = 0). The optimal value of *L*_*p*_ resulting from the minimization is determined by the Equation (15) with:

(29) $$\varepsilon \;=\;-\frac{5(\Lambda \;-\;1)}{\Gamma \;+\;29},$$

which states the linear dependence between the function ε that defines the changes in the shape of the polar caps and the shape parameter Λ, which is determined by the initial shape of the vesicle. For the case of an initially spherical vesicle Λ = 1, Equation (29) establishes that the caps remain spherical under an arbitrary small deformation, this is ε = 0 (see Figure 4).

Note that Γ is greater than −29 when $\tilde{\Sigma }R_{m}^{2}>-28\;-\;{\left(2-C_{0}R_{m}\right)}^{2}$. This means that a wide range of values of the dilatation invariant products *C*_0_*R*_*m*_ and $\tilde{\Sigma }R_{m}^{2}$ give a positive denominator in the Equation (29) and, in this case, the shape of the polar caps will be determined by the value of Λ (see Figure 4): Λ = 1 (or equivalently $\Delta \tilde{p}R_{m}^{3}\;=\;2C_{0}R_{m}-C_{0}^{2}R_{m}^{2}-2\tilde{\Sigma }R_{m}^{2})$ would correspond to invariably spherical polar caps, but Λ < 1 (or equivalently $\Delta \tilde{p}R_{m}^{3}\;<\;2C_{0}R_{m}\;-\;C_{0}^{2}R_{m}^{2}\;-\;2\tilde{\Sigma }R_{m}^{2}$) would correspond to prolate polar caps, and Λ > 1 (or equivalently $\Delta \tilde{p}R_{m}^{3}>2C_{0}R_{m}-C_{0}^{2}R_{m}^{2}-2\tilde{\Sigma }R_{m}^{2})$ would correspond to oblate polar caps. If Γ is less than −29, which corresponds to strongly negative surface tension $2\tilde{\Sigma }R_{m}^{2}<-28-{\left(2-C_{0}R_{m}\right)}^{2}$, then the denominator of Equation (29) takes negative values, and the correlation between the sign of Λ−1 and the shape of the polar caps is inverted. If Γ = −29, the perturbative approach is not valid since gives ε = −∞.

**Figure 5:**
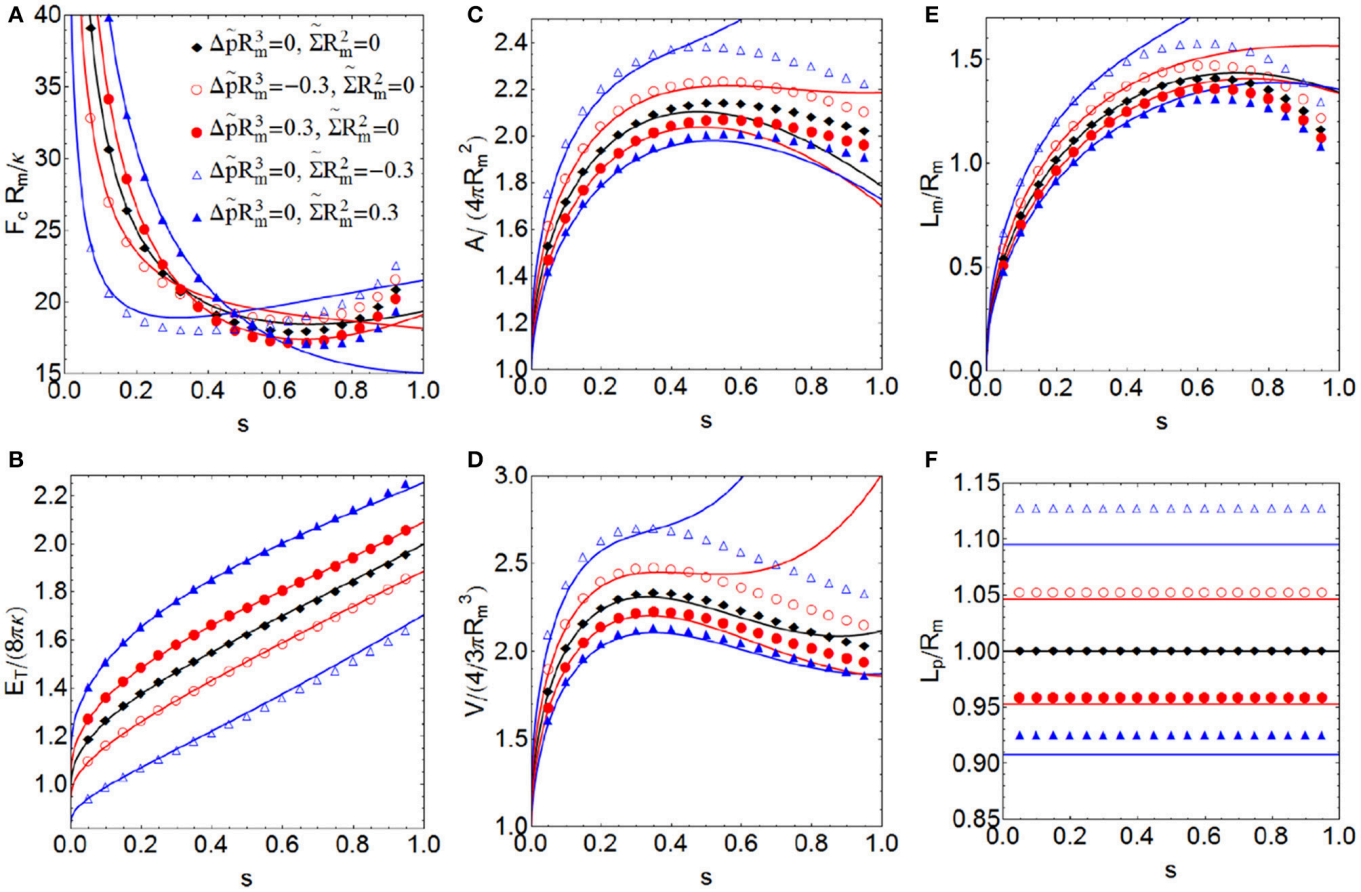
**More relevant properties of a constricted vesicle with zero spontaneous curvature C_0_ = 0 at all stages of constriction for different values of the dilatation invariant products $\tilde{\Sigma }$${\text{R}}_{\text{m}}^{2}$ and $\Delta \tilde{\text{p}}{\text{R}}_{\text{m}}^{3}$, which are associated with surface tension and pressure, respectively**. Total energy *E*_*T*_ in units of 8πκ **(A)**, constriction force *F*_*c*_ in units of *R*_*m*_/κ **(B)**, total area *A* in units of $4\pi R_{m}^{2}$ **(C)**, total volume *V* in units of $4/3\pi R_{m}^{3}$ **(D)**, constriction length *L*_*m*_ in units of *R*_*m*_ **(E)** and polar distance *L*_*p*_ in units of *R*_*m*_ **(F)**. Comparison between the exact numerical results (points) with the approximate analytical expressions obtained up to sixth order of perturbation (lines).

**Figure 6:**
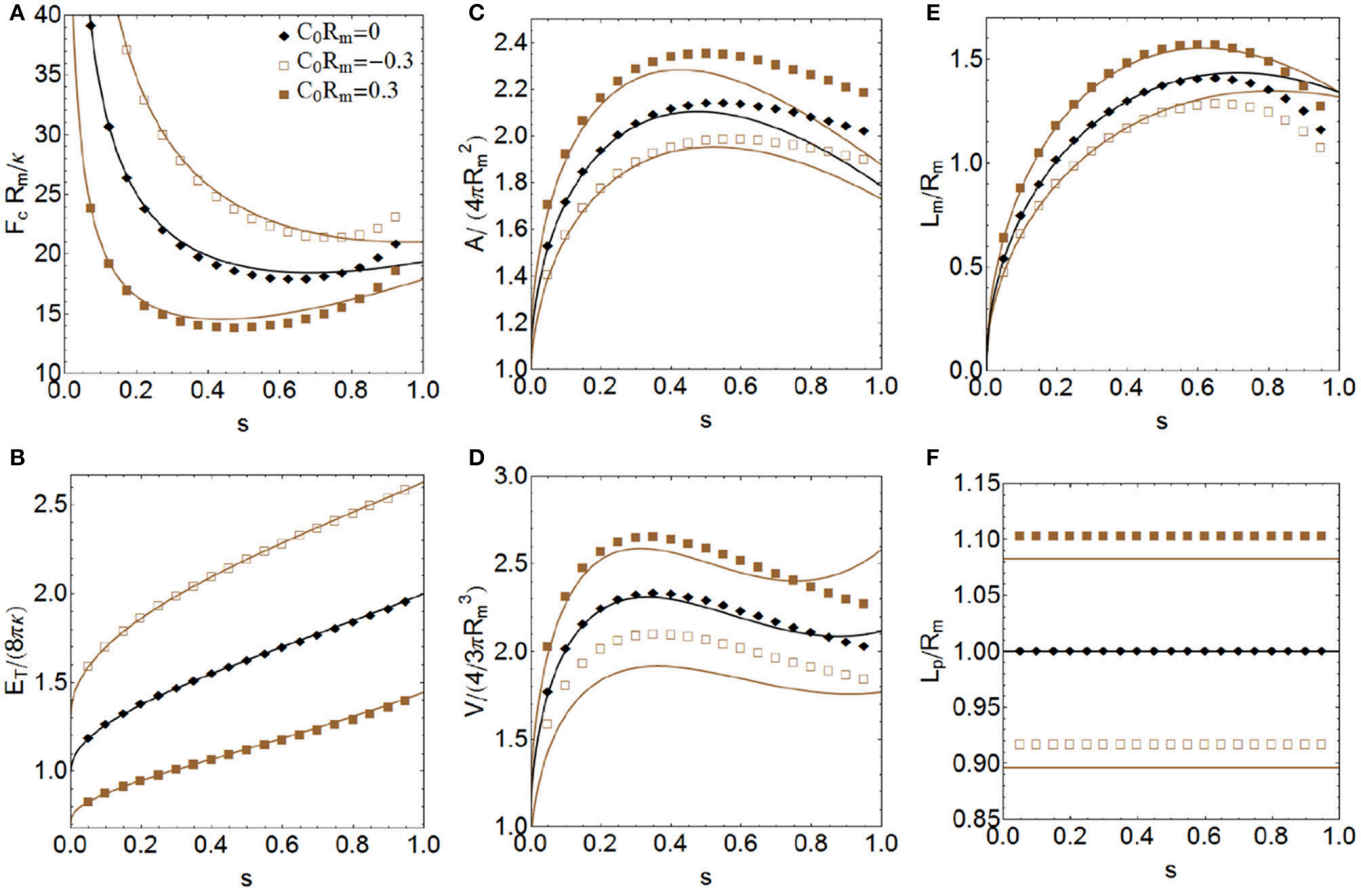
**More relevant properties of a constricted vesicle at all stages of constriction for Σ = Δ***p*** = 0 and three values of the product ***C***_0_***R***_m_, corresponding to have negative, zero, and positive spontaneous curvature**. Total energy *E*_*T*_ in units of 8πκ **(A)**, constriction force *F*_*c*_ in units of *R*_*m*_/κ **(B)**, total area *A* in units of $4\pi R_{m}^{2}$ **(C)**, total volume *V* in units of $4/3\pi R_{m}^{3}$ **(D)**, constriction length *L*_*m*_ in units of *R*_*m*_ **(E)** and polar distance *L*_*p*_ in units of *R*_*m*_ **(F)**. Comparison between the exact numerical results (points) with the approximate analytical expressions obtained up to sixth order of perturbation (lines).

Substituting the perturbative parameter ε (Equation 29) in Equations (27), (16), and (17) we obtain the approximate analytical expressions for the energy of the polar caps, their membrane area, and the volume enclosed on them, respectively. For any combination of values of *C*_0_*R*_*m*_, $\tilde{\Sigma }R_{m}^{2}$, and $\Delta \tilde{p}R_{m}^{3}$ giving |ε| ≪ 1 (Equation 29), the errors between the numerical and the analytical calculations for the polar caps are lower than 5% in all the properties determined. These errors are lower in the cases in which the ratio *L*_*p*_/*R*_*m*_ is closer than 1, since in these cases the perturbative parameter ε becomes smaller in modulus (see Figures 5F, 6F).

#### Constriction zone

Integrating Equation (21) between the limits *x*_*i*_ = −*L*_*m*_ and *x*_*f*_ = *L*_*m*_ (see Figure 3A), we obtain an approximate analytical expression for the total energy of the constriction zone. Minimizing this energy with respect to *L*_*m*_ (Equation 12b) allows deriving the perturbative expansion for the optimal value of the constriction length:

(30) $$\begin{array}{l} L_{m}(s)/R_{m}\approx \frac{\pi }{2}{\left(\frac{6}{\Lambda }\right)}^{1/4}s^{1/2}\{1\;+\;\frac{1}{288\Lambda }[72(\Lambda \;-\;\Gamma )\\ \;+(\Gamma \;-\;4)6^{3/2}\Lambda^{1/2}\;-256^{1/2}\Lambda^{3/2}]s\;+\ldots \} \end{array}$$

This formula determines the aspect ratio of the shape of minimal energy for each constriction stage, characterized by the constriction parameter *s*, up to fourth-order in the perturbative expansion (see Section 2 of SM for a higher-order formula).

Substituting the optimal constriction length in the equation for the total energy (Equation 6), we obtain the approximate analytical expression for the increase in the total energy due to constriction:

(31) $$\begin{array}{l} \Delta E_{T}(s)/\kappa \;=\;E_{T,\text{cz}}(s)/\kappa \;\approx \;\frac{4}{3}\pi^{2}6^{1/4}\Lambda^{3/4}s^{1/2}\\ \;\{1-\frac{3}{576\Lambda }[56^{1/2}\Lambda^{3/2}\;+\;168\Lambda \\ \;-\;(\Gamma \;-\;4)6^{3/2}\Lambda^{1/2}72\Gamma ]s\;+\;\ldots \}. \end{array}$$

This expression is written up to fourth order in the perturbative expansion and a higher-order formula is shown in Section 2 of SM.

Once we have calculated the dimensionless ratio *L*_*m*_/*R*_*m*_ (Equation 30), we can determine the membrane area of the constriction zone and the volume enclosed on it. Integrating the Equation (22) between the constriction zone limits *x*_*i*_ = −*L*_*m*_ and *x*_*f*_ = *L*_*m*_ (see Figure 3A) with *L*_*m*_ given by the Equation (30), we obtain the increase of area during constriction:

(32) $$\begin{array}{l} \Delta A(s)/R_{m}^{2}\;=\;A_{\text{cz}}(s)/R_{m}^{2}\;\approx \;2\pi^{2}{\left(\frac{6}{\Lambda }\right)}^{1/4}{\text{s}}^{1/2}\\ \;\{1\;+\;\frac{1}{576\Lambda }[(\Gamma \;-\;4)6^{3/2}\Lambda^{1/2}\;-\;72\Gamma \;\\ \;\;-\;216\Lambda \;-6^{1/2}\Lambda^{3/2}]s\;+\;\ldots \}. \end{array}$$

As for other quantities, this is a fourth-order perturbation expression but a higher-order formula can be found in Section 2 of SM. At the initial stages of constriction (when *s* → 0) the increase of area is given by the leading term of the Equation (32), i.e., $2\pi^{2}{R_{m}^{2}\left(6/\Lambda \right)}^{1/4}s^{1/2}$, which is equal to π*R*_*m*_ × 2*L*_*m*_ with *L*_*m*_ given by the leading term of the Equation (30). Since 2π*R*_*m*_ × 2*L*_*m*_ corresponds to the increase of area of a cylinder of length 2*L*_*m*_ and polar radius *R*_*m*_, this means that that a near-cylindrical neck with length 2*L*_*m*_ is formed at the initial stages of constriction (see Figure 4).

Similarly, integrating the Equation (23) between the constriction zone limits *x*_*i*_ = −*L*_*m*_ and *x*_*f*_ = *L*_*m*_ (see Figure 3A) with *L*_*m*_ given by the Equation (30), we obtain the approximate analytical expression for the increase of volume during constriction up to fourth-order in the perturbative expansion of the energy integrand (see Section 2 of SM for higher-order expression):

(33) $$\begin{array}{l} \Delta V/R_{m}^{3}\;=\;V_{\text{cz}}/R_{m}^{3}\;\approx \;\pi^{2}{\left(\frac{6}{\Lambda }\right)}^{1/4}{\text{s}}^{1/2}\{1\;+\;\frac{1}{576\Lambda }\\ \;[(\Gamma \;-\;4)6^{3/2}\Lambda^{1/2}\;-\;72\Gamma \;-\;504\Lambda \\ \;-256^{1/2}\Lambda^{3/2}]s\text{+}\ldots \}. \end{array}$$

Analogously to variations of area, we find that the increase of volume at the beginning of constriction (*s* → 0) is given by the leading term of the Equation (33), i.e., $\pi^{2}{R_{m}^{3}\left(6/\Lambda \right)}^{1/4}{\text{s}}^{1/2}$, which is equal to $2\pi R_{m}^{2}\times 2L_{m}$ with *L*_*m*_ given by the leading term of the Equation (30). This product corresponds to the increase of volume of a cylinder of length 2*L*_*m*_ and polar radius *R*_*m*_, which again corresponds with having a near-cylindrical neck with length 2*L*_*m*_ at the initial stages of constriction (see Figure 4). Once we have calculated the properties for the polar caps and constriction zone, we sum both contributions to obtain the total values.

Finally, we determine the constriction force from the derivative of the total energy with respect to constriction radius:

(34) $$F_{c}\;\equiv \;-\frac{dE_{T}}{dR_{c}}\;=\;-\frac{dE_{T}}{ds}\frac{ds}{dR_{c}}\;=\;\frac{1}{R_{m}}\frac{dE_{T}}{ds},$$

which gives

(35) $$\begin{array}{l} F_{\text{c}}(s)R_{m}/\kappa \approx \;\frac{2\pi^{2}6^{1/4}\Lambda^{3/4}}{3s^{1/2}}\{1\;-\;\frac{1}{192\Lambda }[56^{1/2}\Lambda^{3/2}\;+\;168\Lambda \\ \;-\;(\Gamma \;-\;4)6^{3/2}\Lambda^{1/2}-72\Gamma ]s\;+\;\ldots \}. \end{array}$$

The constriction force scales inversely proportional to *R*_*m*_, i.e., the smaller is the vesicle, the greater is the constriction force required. In other words, smaller cells are harder to constrict. In contrast, the force required to constriction scales proportional to the bending modulus κ. This implies stronger constriction forces for less flexible membranes. In the general case, in which the parameters *C*_0_, Σ, and Δ*p* are non-zero, the analytical expressions obtained are divided by powers of Λ^1/4^ (see Equations 30–33, and 35), which implies a divergence in the results when Λ → 0. Therefore, our analytical approach is valid as far as the values of the dilatation invariant products *C*_0_*R*_*m*_, $\tilde{\Sigma }R_{m}^{2}$ and $\Delta \tilde{p}R_{m}^{3}$ do not give a Λ close to zero (Equation 24), and the deformation functions used in the perturbative expansion (ε for polar caps and *u* for constriction zone) are much lower than 1 in modulus. Conditions giving Λ < 0 (Equation 24) (or equivalently $\Delta \tilde{p}R_{m}^{3}<2C_{0}R_{m}-C_{0}^{2}R_{m}^{2}-2\tilde{\Sigma }R_{m}^{2}-1$) provide complex analytical results. This means that the analytical method cannot be applied under conditions with Λ ≤ 0.

There is a good agreement between the exact results and the approximate analytical expressions for low and intermediate constriction regimes, (approximately up to *s* ≈ 0.65), (see Figures 5, 6). This indicates that the ansatz used to parameterize the constriction zone (Equation 19) is extremely efficient in describing the exact result in these stages (as it was in Almendro-Vedia et al., 2013, 2015, for a more particular case). For higher constrictions, the errors are bigger, as a consequence of the zeroth-order function family assumed for the ansatz (Equations 18 and 19). Results that were more accurate would require a constriction profile more precise than Equation (19), including more terms of the family of solutions in order to better accounts for the strong changes of curvature occurring in the constriction zone. The analytical result for the case with $\tilde{\Sigma }R_{m}^{2}\;=\;-0.3$ and *C*_0_ = Δ*p* = 0 (empty blue triangles of Figure 5) differs from the exact numerical values more than the other cases. The reason is that this combination of parameters gives the closer-to-zero value of Λ and the analytical formulas diverge as Λ goes to zero.

### Osmotic pressure and surface tension effects with no spontaneous curvature

In this subsection, we analyze the effects of osmotic pressure Δ*p* and surface tension Σ in the more relevant properties of a membrane vesicle with zero spontaneous curvature. The lower values of surface tension and the osmotic pressure difference, the lower the energies of the vesicle and the smaller constriction forces required (see Figures 5A,B, respectively). This means that membranes with small or negative tension (Σ ≤ 0) and immersed in a hypertonic medium (Δ*p* < 0) have less energy and constrict more easily than tensioned membranes (Σ > 0), immersed in an isotonic or hypotonic medium (Δ*p* ≥ 0). As we noted in Almendro-Vedia et al. (2013, 2015), a kick-off force is required to initiate constriction from the initial configuration. However, once the symmetry is initially broken, smaller forces are sufficient to advance cell constriction. At the high constriction regime, the constriction force increases in order to overcome the curvature barrier involved in the pre-fissioned state (see Figure 5B). The total energy of the system (see Figure 5A) increases along the constriction pathway up to double at maximal constriction (when *s* → 1). In the final two-spheres fission state we have to consider the additional Gaussian curvature energy contribution of 4πκ_*G*_ ≈ −100*k*_*B*_*T*, since κ_*G*_ ≈ −0.8κ (Siegel and Kozlov, 2004; Marsh, 2006), in order to account for the topological change occurred. Inflated vesicles immersed in a hypotonic medium (Δ*p* > 0) have more volume than vesicles immersed in an isotonic medium (Δ*p* = 0), and these last have more volume than shrunk vesicles immersed in hypertonic medium (Δ*p* < 0), (see Figure 5D). This inflation-shrinking process is explained by the osmotic turgor of the living cells. When vesicles (or cells) are placed in a hypotonic medium, water rushes into the membrane increasing the volume of the vesicle. In contrast, when vesicles are placed in a hypertonic solution, water flows out of the vesicle into the surrounding solution, decreasing its volume. When the surface tension is positive, the increase in membrane area is lower under constriction, and vice versa (see Figure 5C). This is explained since positive surface tension implies a tensioned status of the membrane vesicle, which describes biological situations of positive cortical tension with high energetic cost for membrane area extension (Lecuit and Lenne, 2007). Conversely, negative surface tension implies a floppy status, which describes biological situations with a low energetic cost for membrane area extension (Masters et al., 2013). Negative surface tension is equivalent to a net production of membrane, which actually request a negative mechanical work for membrane dilation (Solon et al., 2006). Finally, the lower values of Σ and Δ*p*, the larger the reduced constriction length *L*_*m*_/*R*_*m*_ (see Figure 5E). Moreover, when Δ*p* and Σ are positive, *L*_*p*_/*R*_*m*_ < 1, and the polar caps are oblate (see Figure 5F), which corresponds to an inflated vesicle with a tensioned membrane. In contrast, when Δ*p* and Σ are negative, *L*_*p*_/*R*_*m*_ > 1, and the polar caps are prolate, which corresponds to a deflated vesicle with a tensionless membrane. If Σ = Δ*p* = 0, *L*_*p*_/*R*_*m*_ = 1, then Λ = 1, and the polar caps are spherical. This result let us relate the shape of the polar caps with the properties of the system. Vesicles with oblate polar caps require more constriction force and contain more energy, less membrane area, and less volume enclosed than vesicles with prolate polar caps. The values $\tilde{\Sigma }R_{m}^{2}\;=\;\pm 0.3$ and $\Delta \tilde{p}R_{m}^{3}\;=\;\pm 0.3$ used in Figure 5 correspond to Σ = ±1.2 × 10^−8^ N/m and Δ*p* = ±1.2 × 10^−2^ N/m^2^, respectively, a realistic set of values reasonably compatible with a cell-sized artificial vesicle (*R*_*m*_≈1μm) (Claessensa et al., 2008; Ogleçka et al., 2012) with a relatively flexible lipid bilayer membrane (κ ≈ 10 − 20 *k*_*B*_*T*) (Marsh, 2006; Rodríguez-García et al., 2009; Boal, 2012; Nagle, 2013) (Equation 26). Specifically, giant unilamellar vesicles with sizes ranging a few microns, subjected to osmotic stresses of the order of 10 mOsM as much (Δ*p* < 0.01Pa), normally exhibit a lateral tension of the order of 10^−9^−10^−8^ N/m (Käs and Sackmann, 1991; Rodríguez-García et al., 2009).

### Spontaneous curvature effects

In this subsection, we present the results for the constriction process of a membrane vesicle with negligible surface tension (Σ = 0) and no osmotic pressure difference (Δ*p* = 0) for two values of the product *C*_0_*R*_*m*_, corresponding a positive and a negative spontaneous curvature, respectively. In this way, we can analyze the effects of having a convex (*C*_0_ > 0) or a concave (*C*_0_ < 0) membrane with respect to the flat configuration (*C*_0_ = 0), see Figure 2. Although here we are considering constant spontaneous curvature, recall that, in general, *C*_0_ is not uniform over the membrane of a real cell (Emoto et al., 2005; Renner and Weibel, 2011). The spontaneous curvature has an important effect on the constriction force (see Figure 6B), and a concerted inhomogeneous distribution may play a crucial role in coordinating the contractile rearrangement with the membrane remodeling during cytokinesis (see Discussion). We see that membranes with global positive spontaneous curvatures are more easily constricted (require smaller constriction forces) than flat membranes. In contrast, membranes with global negative spontaneous curvature need higher constriction forces. As in Section Osmotic Pressure and Surface Tension Effects with No Spontaneous Curvature, we can relate the constriction force required with the shape of the polar caps (see Figure 6F). Vesicles with oblate polar caps (when *L*_*p*_/*R*_*m*_ < 1) require more constriction forces than vesicles with prolate polar caps (when *L*_*p*_/*R*_*m*_ > 1). The total energy of the vesicle increases as a function of the stage of constriction up to near double its value at the final stage (see Figure 6A). The energy of the vesicles whose membranes have global negative spontaneous curvature is greater than the energy in the flat configuration, while for membranes with global positive spontaneous curvature the energy is lower. As Σ = Δ*p* = 0, the total energy of the vesicle in Figure 6A is exclusively the bending energy. As in Section Osmotic Pressure and Surface Tension Effects with No Spontaneous Curvature, in the final fissioned state we have to consider the additional Gaussian curvature energy contribution of 4πκ_*G*_ ≈ −100*k*_*B*_*T* (Siegel and Kozlov, 2004; Marsh, 2006), in order to account for the topological change. The increase of the membrane area, vesicle's enclosed volume and constriction length along the constriction pathway is shown in Figures 6C–E, respectively. Vesicles with *C*_0_ < 0, which have oblate polar caps (see Figure 6F), have less area, less volume, and less constriction length than vesicles with *C*_0_ > 0, which have prolate caps. The values *C*_0_*R*_*m*_ = ±0.3 used in Figure 6 correspond to $C_{0}\;=\;\pm \;0.3\mu {\text{m}}^{-1}$ for a cell-sized vesicle (*R*_*m*_ ≈ 1μm).

**Figure 7:**
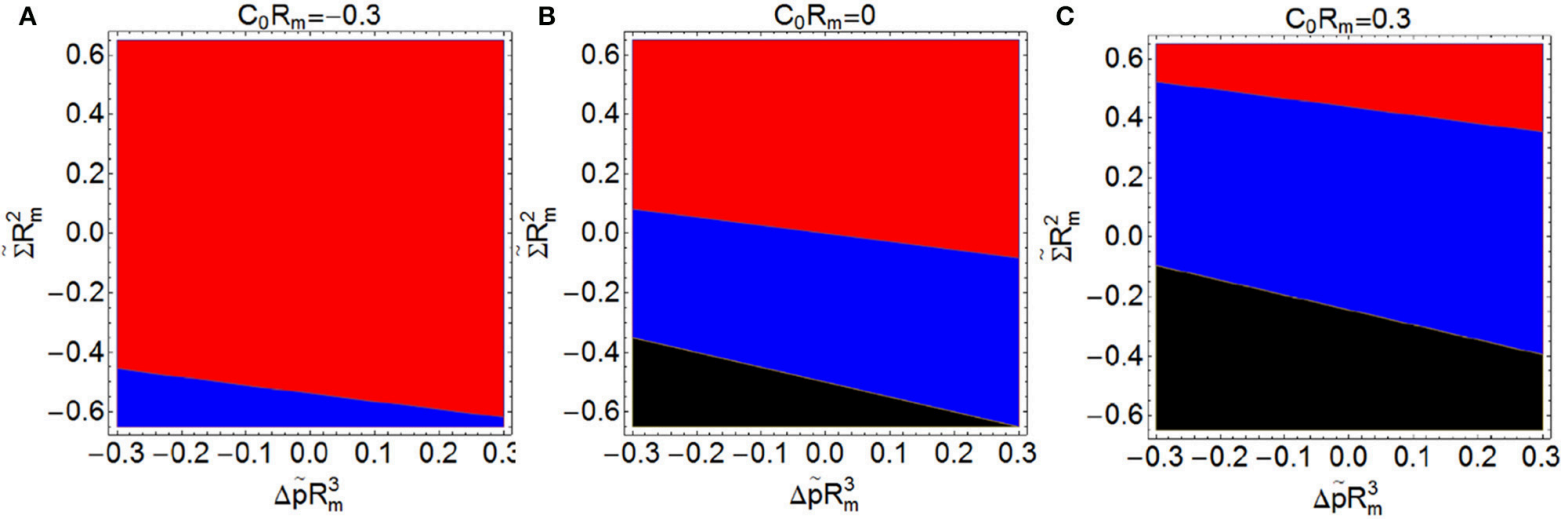
**Constriction force ***F***_***c***_ compared with the reference constriction force ***F***_***c***, 0_ (defined as the constriction force in the case with ***C***_0_ = Σ = Δ***p*** = 0), both forces are computed at the beginning of constriction (***s*** = 0.2)**. (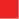 *F*_*c*_ > *F*_*c*, 0_, 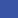 *F*_*c*_ < *F*_*c*, 0_, 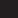 *F*_*c*_ imaginary: impossible constriction). Constriction force is shown as a function of $\tilde{\Sigma }R_{m}^{2}$ (*y*-axis) and $\Delta \tilde{p}R_{m}^{3}$ (*x*-axis) with *C*_0_*R*_*m*_ = −0.3 **(A)**, *C*_0_*R*_*m*_ = 0 **(B)**, and *C*_0_*R*_*m*_ = 0.3 **(C)**. Regions shaded in blue (red) correspond to conditions giving constriction forces lower (larger) than *F*_*c*, 0_ and regions shaded in black correspond to conditions under which constriction is impossible (imaginary analytical results and no numerical solution).

Finally, we address the analysis of the constriction force when *C*_0_*R*_*m*_, $\tilde{\Sigma }R_{m}^{2}$, and $\Delta \tilde{p}R_{m}^{3}$ are different from zero simultaneously. Figure 7 shows three plots with the different regimes of spontaneous curvature: 7A with *C*_0_*R*_*m*_ = −0.3, 7B with *C*_0_*R*_*m*_ = 0, and 7C with *C*_0_*R*_*m*_ = 0.3, varying $\tilde{\Sigma }R_{m}^{2}$ between -0.6 and 0.6 (*y*-axis) and $\Delta \tilde{p}R_{m}^{3}$ between −0.3 and 0.3 (*x*-axis). We have calculated the constriction force at the beginning of constriction (*s* = 0.2) and compare it with the reference constriction force *F*_*c*, 0_, defined as the constriction force at this stage in the case of *C*_0_ = Σ = Δ*p* = 0. For given values of Σ and Δ*p*, the larger the positive spontaneous curvature, the smaller is the constriction force required (see Figure 7B). This means that prolate-shaped elongated shapes whose membranes have an uniform *C*_0_ > 0 are more easily constricted, i.e., those cells tend globally to build up in a convex configuration. Membranes with positive spontaneous curvature tend to form vesicles of smaller radius 2/*C*_0_, which favors the formation of two separated vesicles. Thus, introducing a positive spontaneous curvature extends the region of negative constriction force, i.e., the region where constrictions is an energetically favorable process (see Figure 7).

**Figure 8:**
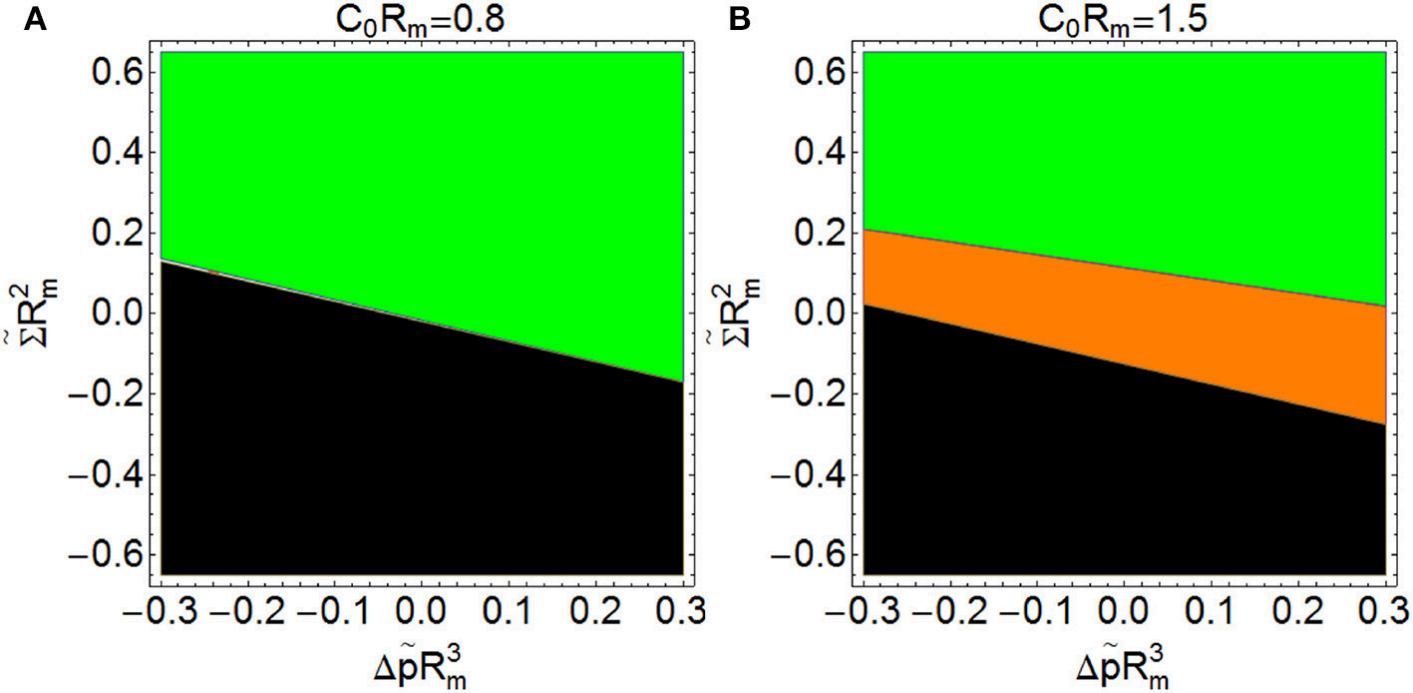
**Constriction force at the beginning of constriction (***s*** = 0.2) as a function of $\tilde{\Sigma }R_{m}^{2}$(*y*-axis) and $\Delta \tilde{\text{p}}R_{m}^{3}$ (x-axis) with ***C***_0_***R***_***m***_ = 0.8 (A)** and with *C*_0_*R*_*m*_ = 1.5 **(B)**. (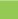 *F*_*c*_ > 0: Non-spontaneous constriction, 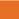 *F*_*c*_ < 0: Spontaneous constriction, 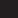 *F*_*c*_ imaginary: impossible constriction). The regions shaded in green correspond to the cases in which an external force is required for constriction. The region shaded in orange corresponds to conditions leading to spontaneous constriction (negative constriction forces). Finally, the regions shaded in black correspond to conditions under which constriction is impossible (imaginary analytical results and no numerical solution. If *C*_0_*R*_*m*_ > 1, it is possible to get spontaneous constriction for a certain range of the products $\tilde{\Sigma }R_{m}^{2}$ and $\Delta \tilde{p}R_{m}^{3}$, but if *C*_0_*R*_*m*_ < 1 there is no combination of surface tension and osmotic pressure leading to spontaneous constriction.

When the product *C*_0_*R*_*m*_ is greater than 1 (this is, when membranes tend to form vesicles with radius smaller than 2*R*_*m*_, or cylinder sections with radius smaller than *R*_*m*_), it is possible to get spontaneous constriction for a certain range of the products $\tilde{\Sigma }R_{m}^{2}$ and $\Delta \tilde{p}R_{m}^{3}$ (see Figure 8B, shaded in orange). This means that spontaneous constriction can be induced with appropriate low values of surface tension and osmotic pressure. If the product *C*_0_*R*_*m*_ is smaller than 1 (this is, if membranes tend to form vesicles with radius bigger than 2*R*_*m*_, or cylinder sections with radius larger than *R*_*m*_), there is no combination of surface tension and osmotic pressure leading to spontaneous constriction (see Figure 8A).

### Constant area and constant volume conditions

Instead of considering the polar radius *R*_*m*_ constant, other conditions as constant area or constant volume could be additionally addressed. In these cases, a re-dimensioning strategy can be used, as described in the previous works (Almendro-Vedia et al., 2013, 2015) defining a rescaling parameter λ with the following scaling transformations κ → κ, *E*_*T*_ → *E*_*T*_, *C*_0_ → *C*_0_/λ, *C*_1_ → *C*_1_/λ, *C*_2_ → *C*_2_/λ, *A* → λ^2^*A*, *V* → λ^3^*V*, Σ → Σ/λ^2^, and Δ*p* → Δ*p*/λ^3^. This parameter corresponds to:

(36) $$\lambda (\text{constant }A)\;=\;\sqrt{\frac{A(s\;=\;0)}{A(s)}},$$

for constant area condition and to:

(37) $$\lambda (\text{constant }V)\;=\;\sqrt[{3}]{\frac{V(s\;=\;0)}{V(s)}},$$

for constant volume condition. *A*(*s* = 0) and *V*(*s* = 0) are, respectively, the membrane area and the volume enclosed by the initial spheroid.

## Discussion

### Lipid bilayer membrane vesicles

Lipid molecules dispersed in water have the property to self-assemble spontaneously into a bilayer membrane. The lipid bilayer constitutes the main structural ingredient of cell membranes, which endows them with a functional mechanics chiefly determined by its intrinsic elasticity and the curvature properties encoded in the topology of the molecular components (Bretscher, 1973). The resistance of bilayers to area compression and area expansion is much larger than their resistance to bending deformations, while, in the fluid state, there is no resistance to shear deformations. The bending rigidities of usual lipid bilayers in the fluid state take values about 10 − 20 *k*_*B*_*T* (Marsh, 2006; Rodríguez-García et al., 2009; Boal, 2012; Nagle, 2013). The combination of the minimization concepts discussed in this paper has allowed the systematic exploration of vesicle energetics under constriction geometry. From our analysis of equatorial constriction (Figure 5B), micron-sized vesicles with a flexible lipid bilayer and a zero spontaneous curvature have constriction forces in the range *F*_*c*_*R*_*m*_/κ ≈ 15 − 20. This corresponds to effective forces of the order of pico-newtons, or even lower, as for κ ≈ 10 *k*_*B*_*T* and *R*_*m*_ ≥ 1μm, one has *F*_*c*_ ≤ 1pN. Inclusion of non-zero values of spontaneous curvature, osmotic pressure and lateral tension leads to significant changes in the specific quantitative conditions necessary for equatorial constriction (see Figures 6–8). However, no essential change is imposed by these constraints in the qualitative picture, which is almost governed by a monotonic increase of the total energies upon increasing constriction, as in the reference case of no constraints (Σ = Δ*p* = *C*_0_ = 0).

### Global spontaneous curvature

Among the more important biophysical consequences of lipid asymmetries, the subsequent spontaneous curvature of the whole membrane has a crucial impact on the shape transformations of artificial lipid bilayer vesicles (Boal, 2012), which exhibit an extreme sensitivity to induced changes in bilayer asymmetry (Berndl et al., 1990). The simplest description incorporating asymmetry between the two monolayers is given by a non-zero spontaneous curvature *C*_0_, in which case the bending energy becomes dependent of size scale $C_{0}^{-1}$, thus being minimal for initially curved configurations. As a result, the bending energy loses size invariance and becomes depending on vesicle shape and vesicle size. As a consequence, different axisymmetric shapes of minimal bending energy can be obtained by breaking various symmetries of the sphere. Allowing for asymmetry as well as reflection symmetry, one can obtain prolate and oblate ellipsoids, which are defined by rotating an ellipse about its major and its minor axes, respectively. Negative spontaneous curvature (*C*_0_ < 0) determines polar caps with a predominant oblate shape (Λ > 1); conversely, positive spontaneous curvature (*C*_0_ > 0) determines prolate shapes elongated along the *x* axis (Λ < 1). Because of the lower energy changes of the prolate shape when subjected to equatorial constriction (see Figures 5B, 6B), the radial forces needed to constrict are significantly smaller in the case of prolate shapes (Λ < 1) than in oblates (Λ > 1). According to Equation (2), the energies of vesicle shapes depend on the value of the spontaneous curvature in addition to membrane tension and osmotic pressure difference, thus, we need to expand our parameter space to three dimensions. The results of minimal energy calculations performed on the basis of Equation (2) subjected to the three parameters (*C*_0_, Δ*p*, Σ) are summarized in Figures 5, 6. Total energies increase with increasing constriction from a value compatible with a single vesicle to a 2-fold value compatible with vesicle fission. The sharper energy changes under initial constriction (s < 0.2) are observed for inflated oblates (Δ*p* ≥ 0, Σ ≥ 0, *C*_0_ < 0 thus Λ > 1), which demand on higher positive constriction forces than in the reference case of a floppy spherical vesicle (Λ = 1). For prolates shapes (Λ < 1), the required constriction forces are, in general, smaller than for oblates. Obviously, prolates shapes are easier contractible and stretchable than oblates, which explains the calculated decrease of the constriction force with increasingly positive spontaneous curvature. At large constriction (s → 1), however, total energies vary almost linearly in all cases, which implies a very similar constriction force range *F*_*c*_*R*_*m*_/κ ≈ 20, in the whole space of parameters. For a vesicle (or cell) of micrometer size with a flexible membrane with κ ≈ 10 − 20 *k*_*B*_*T* (Marsh, 2006; Rodríguez-García et al., 2009; Boal, 2012; Nagle, 2013), in case of favoring constriction under positive global curvature preferring convex prolates shapes, the constriction forces fall in the range of piconewton, below the value expected for zero spontaneous curvature (see Figure 7B).

### Lipid asymmetry and local spontaneous curvature

A non-zero local spontaneous curvature arises primarily from asymmetry factors in the membrane, particularly differences in the shape and aspect of the component lipids between the two sides of the bilayer. Figure 2 depicts the geometry exhibited by some lipid molecules as the driving force that causes spontaneous membrane curvature (Israelachvilli, 1992; Ritacco et al., 2010; Boal, 2012). Most frequent membrane-formers are cylinder-shaped lipid molecules, which are prone to self-assemble as flat bilayers with a zero spontaneous curvature. Lipids with a polar head group area larger than the cross-sectional area measured at the level of the acyl chains show an inverted-cone shape and tend to curve the membrane positively; in other words, they exhibit positive spontaneous curvature and make the membrane prone to convexity. Prototypical of positive-curvature are lyso-phospholipids, which are intermediates in phospholipidic metabolism, resulted from partial hydrolysis and removing one of the acyl chains of the phospholipids. Due to their inverted-cone molecular aspect, these lyso-phospholipids cannot self-assemble as planar bilayers but form inverse hexagonal phase. When incorporated into bilayers, such non-bilayer forming lipids introduce packing stresses, which, in turn, can affect membrane integrity. Although found only in small amounts in biological cell membranes, lyso-phospholipids have a functional role usually related to cell activation and apoptosis (Munder et al., 1979). Conversely, cone-shaped lipids with a small head cross-section as compared to hydrophobic tails, such as polyunsaturated lipids, diacyl glycerol (DAG), phosphatidylethanolamines (PE) and cardiolipin (CL) exhibit a negative spontaneous curvature (Martens and McMahon, 2008), which make the membrane prone to concavity. During cell division, the process of membrane constriction ends up with a separation of the lipid bilayer of the two daughter cells followed by a fusion of the opposite membranes in a region of high concavity that requires dynamic changes of the lateral distribution and the local composition of membrane lipids. Accumulated experimental evidence points out to the possible mechanical role of negative-curvature lipids during late constriction (Emoto et al., 2005; Litvak et al., 2005; van Meer et al., 2008; Donaldson, 2009; Heberle and Feigenson, 2011; Renner and Weibel, 2011). Those findings demonstrate that the localized production of negative-curvature lipids is required for the proper completion of membrane dynamical process in highly negative-curvature sites, where the local accretion of asymmetric lipids may play a crucial role. Therefore, any extended theoretical model of cell division might consider local negative curvature concentrated in the constriction site of the cell membrane. Localized non-zero spontaneous curvature makes the membrane to be locally prone to a specific curvature, convex (*C*_0_ > 0) or concave (*C*_0_ < 0), depending of the sign of the spontaneous curvature (see Figure 2). Since the constriction site is saddle-shaped, local negative values of the spontaneous curvature may contribute to minimize the local bending energy of the membrane, thus making more realistic further models of cell division, specifically, those accounting for the local accretion of negative curvature lipids in the constriction region.

### Budding and spontaneous fission

Binary fission and budding are two scission mechanisms exploited by cells in asexual reproduction pathways. Major difference between binary fission and budding is that in budding there is an asymmetric outgrowth from the parent individual vesicle, or cell, producing a bud, but in binary fission the parent symmetrically splits into two more or less identical offspring. In biological cells, budding is a rather frequent event, because it represents the first step in the production of transport vesicles which shuttle between different compartments of the cell. The simplest approach to understand budding involves a consideration of the lateral and transverse organization of lipids within a membrane, which induces spontaneous curvature followed by morphological change. In an early hypothesis to bud formation, Sheetz and Singer suggested that a local change in the surface area of the two monolayers could lead to negative membrane curvature, inducing the formation of a membrane neck (Sheetz and Singer, 1974). However, advanced models for bud formation in biological cells emphasize a chief role for membrane coating proteins (Schekman and Orci, 1996). In biological constriction processes, the membrane undergoes large mechanical deformations. Although lipids may serve to define the site of bud emergence (Lipowsky, 1992), or determine the onset of divisional constriction (Emoto et al., 2005; Renner and Weibel, 2011), there is almost certainly through the direct action of force exerting proteins (Cao and Wang, 1990; Bi and Lutkenhaus, 1991), or curvature-inducing protein coats (Schekman and Orci, 1996; Bashkirov et al., 2008; Boucrot et al., 2012), that the membrane is able to undergo the large mechanical deformations involved. However, in model vesicles, weak external perturbations suffice to lift the equilibrium constraints of constant area and volume, rendering lipid membranes susceptible of spontaneous budding and fission (Lipowsky, 1991; Miao et al., 1991). For instance, vesicles made of lipids with a weakly negative spontaneous curvature are known to undergo the budding transition at increasing temperature (Berndl et al., 1990; Käs and Sackmann, 1991; Dobereiner et al., 1993), which is equivalent to expanding membrane area. In this budding transition an initially spherical vesicle transforms, via prolate- and pear-shaped intermediates, into two asymmetric spheres, one with a daughter bud, which remains connected to the reduced mother vesicle by a narrow neck (Berndl et al., 1990; Käs and Sackmann, 1991; Dobereiner et al., 1993). In general, osmotic gradients are known to induce bio-reminiscent morphological transformations in giant unillamelar vesicles (Ogleçka et al., 2012; and refs. therein). In particular, to realize budding in protein-free vesicles made of (zero-curvature) single lipids requires large excess area in a flaccid configuration (i.e., hypertonic conditions and/or negative surface tension), which induces a spontaneous constriction process that initiates with the formation of a neck and terminates in the scission of the bud. From our calculation, budding and fission are events that could occur spontaneously under sufficiently low (or negative) surface tension (see Figure 8). Obviously, initially prolates shapes and hypertonic conditions decrease the onset for negative constriction force, thus favoring spontaneous constriction in a homogenous vesicle. In experiments with giant vesicles asymmetric budding is observed largely more frequent than much rarer events of symmetric fission (Berndl et al., 1990; Käs and Sackmann, 1991; Dobereiner et al., 1993), a reasonable fact since symmetry breaking tends to minimize the bending energy of the constricted vesicle (Almendro-Vedia et al., 2015). A further complexity that makes lipid vesicles prone to budding involves the consideration of the lateral and transverse organization of mixtures of lipids within the membrane. Changes in the amount of membrane surface giving rise to excess area, or spontaneous curvature, could occur by transbilayer flip-flop movement of phospholipids, or by lipid phase separation leading to a change in the lipid packing density (the case of heterogeneous membranes will be addressed in the next subsection). Asymmetric budding and symmetric fission in vesicles made by a mixture of lipids has already attracted much theoretical interest (Seifert, 1993; Kohyama et al., 2003; Sens, 2004). The models are all based on the minimization of the bilayer energy, but also vary depending on the interactions among the lipids in multicomponent systems, which make them to separate into phases or not. For monophasic, homogeneous vesicles, the membrane neck involved in the budding transition is produced by the lipid molecules whose local negative curvature is different from the main lipids of the membrane. If the molecules prefer a negatively curved bilayer, they will favor the formation of a bud. For biphasic, heterogeneous vesicles, a line tension exists between the two phases, trying to reduce the interface length, and favoring asymmetric budding and symmetric constriction, eventually leading to spontaneous fission (Lipowsky, 1992). This heterogeneous scenario will be further discussed the next subsection. Our results point out the easy practical availability of the onset of spontaneity for the budding/fission transition. Indeed, negative constriction forces are required for relatively low values of membrane tension, even for inflated vesicles under moderately positive osmotic pressure (see Figure 8). Obviously, the possibility for spontaneous budding/fission enhances in prolates shapes defined by high positive values of the global spontaneous curvature (see Figures 7, 8), a fact already recognized in the early studies of the morphological transitions of membrane vesicles (Berndl et al., 1990; Lipowsky, 1991; Seifert and Lipowsky, 1995). The current study allows for quantitatively determining the specific conditions for spontaneous budding/fission from very accurate analytic formulas, which provide an interesting predictive framework for the design of smart vesicle microsystems endowing the division functionality (Osawa et al., 2008).

### Biological membranes

Any cellular membrane, even in the simplest organisms like bacteria, actually consists of a complex mixture of structural lipids, proteins, and a small amount of functional glycolipids and glycoproteins involved in membrane signaling and trafficking. In the simplest mechanical depiction, a realistic cellular membrane might be modeled as a composite shell (Sackmann et al., 2002), composed by a heterogeneous lipid bilayer and adjoined cortical protein, or glycoprotein structures, such as the inner cell cortex in eukaryotes, or the outer peptidoglycan cell wall in bacteria. Such cortical structures can be described as a rigid cover somewhat connected to the fluid bilayer. From the mechanical standpoint, those rigid structures not only strengthen the lipid bilayer against the bending deformation but also bears in-plane shear, which is not supported by the fluid lipids. If the membrane skeleton, or the bacterial wall, are roughly considered to be structurally continuous, the composite cellular shell can still be regarded as a 2D continuum medium, mechanically described by the current material constitutive modeling, eventually accounting for lateral heterogeneity, plus an additional elasticity modulus describing in-plane shear rigidity. In addition, subcellular localization of the cytokinetic apparatus and related proteins is a universal feature of any prokaryote or eukaryote cell. However, although some targeting anchors are known in some organisms, the origin of polar and division-site localization remains mysterious for a large fraction of cytokinetic proteins. Ultimately, the molecular components responsible for such symmetry breaking must employ a high degree of self-organization, which could contribute with additional ingredients to the mechanics of division. For instance, curvature-induced stabilization mechanisms, based on the spontaneous curvature of localized membrane components, have been proposed to account for spontaneous lipid targeting to the poles and division site of rod-shaped bacterial cells (Huang et al., 2006). In that model, if one of the membrane components has a large intrinsic curvature, the geometrical constraint of the inner lipid membrane by the more rigid bacterial cell wall naturally leads to lipid phase separation, and the resulting clusters of high-curvature lipids are large enough to spontaneously localize at cell poles and division site (Huang et al., 2006), in agreement with the experimental evidence of localization of the phospholipid cardiolipin to the negatively curved regions of *E coli* membranes (Renner and Weibel, 2011), and polar targeting of some cytokinetic proteins during bacterial division (Huang et al., 2006). In general, aggregates of lipids, proteins, or lipid-protein complexes may localize in response to cell geometry, introducing additional ingredients of membrane mechanics that might be accounted for. Although all this complexity might be included on an extended, more realistic, model of cell division, the physical problem exceeds the limits of the present work, which can be however considered as a good starting point to obtain approximate solutions that offer a general depiction of the minimal energy mechanical pathway of divisional constriction in different organisms under different geometrical and constitutive conditions of their cellular membranes.

### Toward an integrated mechanical model of cell division

The big question, which remains still to be addressed in a comprehensive way, is to know how much of the division of real cells can be understood in terms of a simplified physical model integrating the passive mechanics of the membrane with the active actuation of a cytokinetic engine. In a minimalistic perspective, the division machinery should have evolved to fulfill, at least, the work requirements of reorganizing the cellular plasma membrane along the cell cycle, especially during cytokinesis. Certainly, the membrane deformations involved along the constriction stage of cell division in biological cells request of an expenditure of mechanical work exerted by a cytokinetic engine, which could however be working under conditions of minimal energy consumption, or perhaps nearing the onset for spontaneous constriction.

Evidence of cell division without the action of a cytokinetic machinery is already accepted to exist in complete kingdoms of archea, and in some species of bacteria including *Chlamydia* and *Planctomycetes* among others (Erickson and Osawa, 2010). The bacterial cytokinetic protein FtsZ is not present in all those organisms, usually called *Dftsz* (devoid FtsZ), which cells are able however to undergo membrane constriction and develop two daughters till mature division without FtsZ. Although *Dftsz* cells spend longer times in division, the constriction slowing-down does not affect their overall growth rate (Erickson and Osawa, 2010). All *Dftsz* bacteria that divide without FtsZ apparently use their motile apparatus to pull the two daughters apart. Under division the constriction neck appears very elongated, with the pair of daughters connected by a thin extension, similarly to the predictions of our current model at low internal pressures Δ*p* < 0, implying Λ < 1 (Equation 24) (see Figure 4, first column). Longitudinal traction-mediated cytofission appears to be a competent mechanism for cell division in bacterial cells lacking FtsZ. In those cases, the mechanics of the bending deformations coupled with the osmotic conditions should be essential for the correct understanding of this minimal work-demanding divisional mechanism. Spontaneous constriction via excess membrane area (see Sections Spontaneous Curvature Effects and Budding and Spontaneous Fission) seems to be a mechanism for nearly-spontaneous binary fission in some mutant strains of *B. subtilis*, especially L-forms that grow without a cell wall and divides without FtsZ (Leaver et al., 2009). Detailed observation of the division process of L-forms of *E. coli, Listeria*, and *B. subtilis* reveals a two-step mechanism that exploits the large excess area of those *Dftsz* cells, initiating the process by a membrane extrusion phase that leads to a long protrusion, which resolves by cleaving into smaller round progenies (Leaver et al., 2009; Erickson and Osawa, 2010). A similar nearly-spontaneous division mechanism was discovered in the eukaryote *Dictyostelium* when its myosin II gen was knocked out (De Lozanne and Spudich, 1987; Knecht and Loomis, 1987). When those myosin lacking cells (thus no cytokinetic motor is working out) are allowed to adhere to a substrate, adhesion forces restore many features of normal furrow constriction and the cells become able to undergo “illegitimate division” by following the same mechanism of traction-mediated cytofission observed in *Dftsz* bacteria.

Constant maximum radius condition may give a simplified description of cytokinesis of rod-shaped cells, like *E. coli* and *Bacillus subtilis*, in which the constant maximum radius is maintained by an external tension (due to a peptidoglycan wall) and represented in our model by the line tension *s*_*m*_. Under this condition, our model predicts unchanged poles during all the constriction process, which is consistent with the approximately constant shape of the poles in the rod-shaped cells (Field et al., 1999; Cabeen and Jacobs-Wagner, 2005; Reshes et al., 2008).

In addition, our model can be extended to other cases, as constant volume and constant area conditions (see Section Constant area and Constant Volume Conditions). Constriction at constant volume requires a nearly 30% increase in area (Almendro-Vedia et al., 2013), and may describe divided cells with intense membrane trafficking (Morré, 1975; Nohturfft and Zhang, 2009), which is known to play an important role in cytokinesis, (Albertson et al., 2005; Boucrot and Kirchhausen, 2007). On the other hand, if constriction takes place at constant area, the volume must be reduced in approximately 30% (Almendro-Vedia et al., 2013), which may describe divided cells with low or inhibited membrane trafficking. Thus, in constant area constriction, a greater initial area is required to have the same final volume. Heat shock has been shown to increase the area before division (Kutalik et al., 2005; Niven et al., 2008) and to affect membrane trafficking molecules genes expression, but also other genes as those of signaling molecules (Kim et al., 2011).

Normal cell division in evolved cells involves a mature cytokinetic engine able to exert the constriction forces that cleave the cell in the division site. This is an up-hill process that requires the expenditure of an important amount of mechanical work by a constriction machinery. However, other concomitant, perhaps redundant, constriction mechanisms could be working to favor the membrane constriction phase of cell division. Redundant systems are often exploited indeed by living cells (Edelman and Gally, 2001). As refers the constriction process during cell division, local creation of negative spontaneous curvature (*C*_0_ < 0) might contribute to favor constriction; both, hypertonic stresses (Δ*p* < 0) and biogenic processes of membrane creation (Σ < 0) mediated by lipid trafficking also favor constriction. In addition, biological cells undergoing divisional constriction there used to be subjected to area and volume restrictions during the cytokinetic phase of the cell cycle. Despite of his inherent simplicity, all these ingredients are already accounted for by our physical model, which could be used to determine the energy landscape for whole configurational space of geometrical characteristics and constitutive properties captured for different classes of cells, from bacteria to eukaryotes. The optimal pathways for the mainstream mechanism of membrane constriction can be identified on this mechanical landscape, and predictions about changing external field parameters (osmotic stress, membrane tension, etc.) could be realized and checked in view of the experimental observations. Further complexities arising from heterogeneous membrane composition and additional shear rigidity introduced by skeletal structures might contribute to complete the picture.

## Conclusions

We have derived general formulas for the more relevant properties involved in the constriction process of a vesicle in terms of the spontaneous curvature of the membrane, the surface tension and the osmotic pressure difference between internal and external environments. These approximate solutions to the constricted shape are valid in the limit where bending, pressure and tension works are comparable, i.e., in the regime where $\Delta pR_{m}^{3}\approx \Sigma R_{m}^{2}\approx \kappa {\left(1-C_{0}R_{m}\right)}^{2}$. Combining a perturbative expansion for small deformations with a variational approach, analytical expressions are obtained and compared with the exact results from numerical computations, getting a good agreement for all the properties calculated in a broad range of constriction stages. The spontaneous curvature of the membrane allows describing vesicles (or simplified cells) with compositional inhomogeneities in its two monolayers, which result in a convex (as *C*_0_ > 0), concave (as *C*_0_ < 0), or flat (as *C*_0_ = 0) membrane in the minimal energy configuration. The surface tension allows describing cellular membranes whose membrane trafficking is present without (as Σ ≤ 0) or with (as Σ > 0) energetic cost (Lecuit and Lenne, 2007; Masters et al., 2013), whereas the osmotic pressure difference represents conditions for an external milieu considered hypotonic (Δ*p* < 0), isotonic (Δ*p* = 0), or hypertonic (Δ*p* > 0) with respect to cytoplasm, which allows describing different turgor states (Campbell et al., 2008). In order to analyze the effects of these parameters (*C*_0_, Σ, and Δ*p*), we have computed the properties of the constricted vesicle for different combinations of values in the regime where these effects and bending energy are comparable. The more interesting results are those corresponding to the force required for constriction, since they show under which conditions vesicles (or cells) constrict more easily (with smaller constriction forces). This analysis is very useful either to understand the physical paths of divisional constriction in living cells or to guide the design of artificial divisomes in self-actuated microsystems. In all cases, if the vesicles (or cells) are of micro size with a flexible membrane with κ ≈ 10 − 20 *k*_*B*_*T* (Marsh, 2006; Rodríguez-García et al., 2009; Boal, 2012; Nagle, 2013), the constriction forces obtained are in the range of picoNewton. This is the range of forces practicable not only by natural divisomes, based on FtsZ rings in bacteria and in actomyosin furrows in eukaryotes, but also by other biomolecular motors. As expected, stronger constriction forces are required for higher values of surface tension and osmotic imbalance, conditions usually present in tensioned membranes of turgid cells, or vesicles. Contrarily, cells, or vesicles, with negative membrane tension, constrict more easily than tensioned membranes with lipid trafficking inhibited. Similarly, shrunk cells, or deflated vesicles, immersed in a hypertonic medium constrict easier than vesicles inflated by an isotonic or hypotonic medium. Furthermore, our analysis demonstrates that *C*_0_ has an important effect on the force required for constriction and vesicles whose membranes have *C*_0_ > 0 (i.e., whose membranes tend to build up in a convex prolate configuration, e.g., cylinder-like bacteria) are the most easily constricted. However, negative values of the local spontaneous curvature, due for instance to local concentrations of lipids with a negatively curvature, make the membranes prone to bend in a neck-like shape with a saddle curvature, so favoring spontaneous budding and symmetric fission. This result gives an idea about the mechanical constraints of the evolution pathway of the biological cell division mechanisms. The method can serve to get insight on other biological processes involving membrane bending, such as exocytosis and endocytosis, and opens a new avenue of material design in the field of bioinspired microsystems with the potential capability to perform the constriction performances intrinsic to the divisional event necessary for self-replication. The proposed method is sufficiently general, and powerful, to accommodate easily further complexities accounting for different membrane asymmetries/heterogeneities present in real cells. This is being the object of ongoing work.

## Author contributions

VA derived the first analytical expressions, implemented numerical computations, and wrote the first extensive notes comparing analytical and numerical results. EB derived additional analytical expressions, implemented numerical computations, contributed to the interpretation of the results, and wrote the first draft of the manuscript. FM contributed to the interpretation of the results and to the manuscript writing, specifically the introduction and the discussion section. FC designed and supervised research (proposed analytical method, supervised analytical derivations and numerical implementations), designed the structure of the manuscript, and contributed to the interpretation of the results and to the writing of the manuscript.

## Funding

Financial support from FPU grant 13/02826 (Ministerio de Educación, Cultura y Deporte, Spain), from MINECO (Spain) grants FIS2010-17440 and FIS2015-67765-R (to FJC), and FIS2009-1450-C02-01 and FIS2015-70339-C2-1-R (to FM) and from Comunidad de Madrid (Spain) grant S2009/MAT-1507 (to FM).

### Conflict of interest statement

The authors declare that the research was conducted in the absence of any commercial or financial relationships that could be construed as a potential conflict of interest.

## Footnotes

^1^Gauss-Bonet Formula. Available: http://mathworld.wolfram.com/Gauss-BonnetFormula.html. Accessed 6 January 2017.
