# Supplementary material for: Modeling the Mechanics of Cell Division: Influence of Spontaneous Membrane Curvature, Surface Tension, and Osmotic Pressure
Source: Front Physiol. 2017 May 19;8:312. doi: 10.3389/fphys.2017.00312 (PMC5437162; doi:10.3389/fphys.2017.00312)
Supplement: Supplementary file 1 [file Presentation1.PDF]

Supplementary Material

Modelling the mechanics of cell division: Influence of spontaneous membrane curvature, surface tension and osmotic pressure

E. Beltrán-Heredia[1,2], Víctor G. Almendro-Vedia[1,2], Francisco Monroy[2,3], and Francisco J. Cao[1*]

[1]Departamento de Física Atómica, Molecular y Nuclear, Universidad Complutense de Madrid, Madrid, Spain.

[2]Departamento de Química Física I, Universidad Complutense de Madrid, Madrid, Spain.

[3]Translational Biophysics. Instituto de Investigación Sanitaria Hospital 12 de Octubre (imas12), Madrid, Spain.

*** Correspondence:**
Corresponding Author
francao@ucm.es

1 Algorithm used to compute the exact solution

Any shape of minimal energy under certain boundary conditions can be obtained by solving the corresponding set of Euler-Lagrange equations. We use as independent coordinates to express the surface shape the arc length S along the contour of the shape, and the azimuthal angle ϕ (see Figure S1). The shape can also be given in terms of the tilt angle as a function of the arc length $\psi(S)$. Using geometrical relations, the coordinates X and Z, which are the parallel and the perpendicular distance to the axis of symmetry, respectively, are

$$\dot{X} = -\sin\psi, \text{(S1)}$$

$$\dot{Z} = \cos\psi. \text{(S2)}$$

The total energy can be expressed as

$$E_T = \pi\kappa \int_0^{S_4} L(\psi, \dot{\psi}, Z, \dot{Z}, \gamma) dS, \text{(S3)}$$

with the Lagrange function

$$L \equiv \frac{Z}{2}\left[\dot{\psi} + \frac{\sin\psi}{Z} - C_0\right]^2 + \tilde{\Sigma}Z + \frac{\Delta\tilde{p}}{2}Z^2\sin\psi + \gamma(\dot{Z} - \cos\psi). \text{(S4)}$$

The first term reflects the mean curvature contribution to the bending energy and the second and third term reflects the contributions to the energy due to the effects of the surface tension and the osmotic pressure, respectively. The last term in Eq. (S4), which involves the Lagrange parameter function $\gamma =$

$\gamma(S)$, must be introduced since the variables ψ and Z are related by Eq. (S2). The corresponding Euler-Lagrange equations for the minimization of the total energy E_T are

$$\dot{\psi} = U, \text{(S5a)}$$

$$\dot{U} = -\frac{U}{Z}\cos\psi + \frac{\cos\psi\sin\psi}{Z^2} + \frac{\gamma}{Z}\sin\psi + \frac{\Delta\tilde{p}Z}{2}\cos\psi, \text{(S5b)}$$

$$\dot{\gamma} = (U - C_0)^2/2 - \frac{\sin^2\psi}{2Z^2} + \Delta\tilde{p}Z\sin\psi + \tilde{\Sigma}, \text{(S5c)}$$

$$\dot{Z} = \cos\psi. \text{(S5d)}$$

Applying the appropriate boundary conditions, the system of ordinary differential equations can be solved and the equilibrium shapes can be obtained. The set of conditions which must be satisfied are: at the beginning, $Z(0) = 0$, $\psi(0) = 0$ and $\gamma(0) = 0$; at the end of the left pole, $Z(S_1) = R_m$ and $\psi(S_1) = \pi/2$; at the constriction ring, $Z(S_2) = R_c$ and $\psi(S_2) = \pi/2$; at the end of the right pole, $Z(S_3) = R_m$ and $\psi(S_3) = \pi/2$ and finally, at the end of the shape, $Z(S_4) = 0$ and $\psi(S_4) = \pi$ (see Figure S1). Since the initial condition $Z = 0$ is a singular point of the Eqs. (S5), we use the Taylor expansion of the variables around $S = 0$. Introducing these power series in the Eqs. (S5), and taking into account that $\psi(0) = Z(0) = \gamma(0) = 0$ and $\lim_{S\to 0} \sin\psi/Z = \dot{\psi}(0) = U(0)$, we can obtain for the initial conditions the approximate solution

$$\psi(S \to 0) = U(0)S + O(S^3), \text{(S6a)}$$

$$U(S \to 0) = U(0) + \frac{1}{4}\left[\Delta\tilde{p} + 4\tilde{\Sigma} + U(0)\big(U(0)^2 + {C_0}^2 + 4U(0)C_0\big)\right]S^2 + O(S^4), \text{(S6b)}$$

$$\gamma(S \to 0) = [(U(0) - C_0)^2/2 + \tilde{\Sigma}]S + O(S^3), \text{(S6c)}$$

$$Z(S \to 0) = S + O(S^3). \text{(S6d)}$$

Similarly, considering the Taylor expansion in order to apply the final condition $Z(S_4) = 0$, we can solve the set of Euler-Lagrange equations.

Maintaining the boundary conditions of fixed radius R_m at S_1 and S_3 and R_c at S_2 requires line tensions σ_m and σ_c, respectively. They act in opposite directions: increasing σ_c forces R_c to decrease, thus increasing constriction. This requires an increase of σ_m to maintain constant R_m during the constriction process (Almendro-Vedia *et al.* 2013, Almendro-Vedia *et al.* 2015). If the left and right poles had different polar radius, the line tensions at S_1 and S_3 would be different too. The boundary matching conditions at these points S_1, S_2 and S_3 are given by (Jülicher and Lipowsky 1996)

$$\sigma_m = \gamma^+(S_1) - \gamma^-(S_1) = \gamma^+(S_3) - \gamma^-(S_3), \text{(S7a)}$$

$$\sigma_c = \gamma^+(S_2) - \gamma^-(S_2), \text{(S7b)},$$

where the plus and minus superscripts refer to the value at the right and at the left of the boundary, respectively. The numerical method is found determining the unknown values $U(0)$, S_1, $\gamma^+(S_1)$, S_2,

$\gamma^{+}(S_2)$, S_3, $\gamma^{+}(S_3)$ and S_4 to make the solution verify the boundary conditions. In the case of symmetric constriction γ verifies the symmetric conditions $\gamma^{\pm}(S_3) = -\gamma^{\mp}(S_1)$ and $\gamma^{+}(S_2) = -\gamma^{-}(S_2)$. Once the shape equation, Eqs (S5), is solved for a constriction stage $s = 1 - R_c/R_m$, the main properties of the system can be calculated. The total energy is obtained computing the expressions in Eqs. (S3) and (S4), and the length of the shape from the Eq. (S2). From Eqs. (8) and (9) and using the relations in Eqs. (S1) and (S2), it is obtained that the membrane area can be calculated as

$$A = 2\pi \int_0^{S_4} Z dS, \text{(S8)}$$

and the vesicle volume as

$$V = \pi \int_0^{S_4} Z^2 \sin\psi dS. \text{(S9)}$$

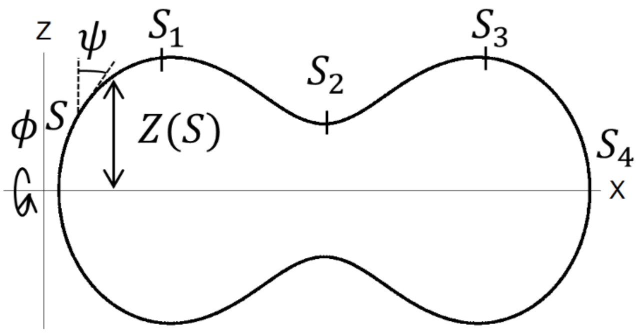

FigureS1. Scheme of the parameterization used for numerical solution.

2 Analytical expressions for constriction zone up to sixth-order perturbative expansions

The quantities of the constriction zone written up to sixth-order of perturbation are

$$
\begin{aligned}
K_{T,\mathrm{cz}} &= \frac{1}{R_m} - 2C_0 + C_0^2 R_m + \Delta\tilde{p} R_m^2 + 2R_m\tilde{\Sigma} \\
&+ \frac{1}{R_m^2}u - C_0^2 u - 2\Delta\tilde{p} R_m u - 2\tilde{\Sigma} u + 2uu_{xx} - 2C_0 R_m u_x \\
&+ \frac{1}{R_m^3}u^2 + \Delta\tilde{p} u^2 + 2C_0 uu_{xx} + R_m u_{xx}^2 - \frac{1}{2R_m}u_x^2 + \tilde{\Sigma} R_m u_x^2 \\
&+ \frac{1}{R_m^4}u^3 - 3u_x^2 u_{xx} + 2C_0 R_m u_x^2 u_{xx} - uu_{xx}^2 - \frac{C_0^2}{2}uu_x^2 - \frac{1}{2R_m^2}uu_x^2 - \tilde{\Sigma} uu_x^2 \\
&+ \frac{1}{R_m^5}u^4 - 2C_0 uu_x^2 u_{xx} - \frac{5R_m}{2}u_x^2 u_{xx}^2 + \frac{3}{8R_m}u_x^4 - \frac{1}{2R_m^3}u^2 u_x^2 - \frac{C_0^2 R_m}{8}u_x^4 \\
&+ \frac{1}{R_m^6}u^5 + \frac{15}{4}u_x^4 u_{xx} - 2C_0 R_m u_x^4 u_{xx} + \frac{C_0^2}{8}u_x^4 u + \frac{3}{8R_m^2}u_x^4 u + \frac{\tilde{\Sigma}}{4}u_x^4 u - \frac{1}{2R_m^4}u_x^2 u^3 \\
&+ \frac{1}{R_m^7}u^6 + 2C_0 uu_x^4 u_{xx} + \frac{35}{8}R_m u_x^4 u_{xx}^2 + \frac{3}{8R_m^3}u_x^4 u^2 - \frac{5}{16R_m}u_x^6 + \frac{C_0^2 R_m}{16}u_x^6 + \frac{R_m\tilde{\Sigma}}{8}u_x^6 - \frac{1}{2R_m^5}u_x^2 u^4 \\
&+ O(u_i^7). \text{ (S10)}
\end{aligned}
$$

$$
\begin{aligned}
L_m(s)/R_m \ &\approx \frac{\pi}{2}\left(\frac{6}{\Lambda}\right)^{1/4} s^{1/2} + \frac{\pi 6^{1/4} s^{3/2}}{1152\Lambda^{5/4}}\left[72(\Lambda-\Gamma) + (\Gamma-4)6^{3/2}\Lambda^{1/2} - 256^{1/2}\Lambda^{3/2}\right] \\
&+ \frac{\pi 6^{1/4} R_m s^{5/2}}{221184\Lambda^{9/4}}\Big[1505\Lambda^3 + 6006^{1/2}\Lambda^{5/2} + 36(7\Gamma + 24C_0R_m + 32)\Lambda^2 \\
&- (7\Gamma + 96C_0R_m + 120)246^{1/2}\Lambda^{3/2} + (\Gamma^2 - 152\Gamma + 576\, C_0R_m - 848)36\Lambda \\
&- 4326^{1/2}\Lambda^{1/2}\Gamma(\Gamma-4) + 4320\Gamma^2\Big] + \cdots. \text{ (S11)}
\end{aligned}
$$

$$
\begin{aligned}
E_{T,\mathrm{cz}}(s)/\kappa &\approx \frac{4}{3}\pi^2 6^{1/4}\Lambda^{3/4}s^{1/2} - \frac{\pi^2 6^{1/4} s^{3/2}}{144\Lambda^{3/4}}\left[56^{1/2}\Lambda^2 + 168\Lambda^{3/2} - (\Gamma-4)6^{3/2}\Lambda - 72\Gamma\Lambda^{1/2}\ \right] \\
&+ \frac{\pi^2 6^{1/4} s^{5/2}}{27648\Lambda^{7/4}}\Big[215\Lambda^{7/2} + 10806^{1/2}\Lambda^3 + (7\Gamma + 24C_0R_m + 800)12\Lambda^{5/2} \\
&- (55\Gamma + 96C_0R_m - 72)246^{1/2}\Lambda^2 + (848 - 576C_0R_m - 232\Gamma - \Gamma^2)36\Lambda^{3/2} \\
&+ (\Gamma-4)1446^{1/2}\Gamma\Lambda - 864\Gamma^2\Lambda^{1/2}\Big] + \cdots \text{ (S12)}.
\end{aligned}
$$

$$
\begin{aligned}
A_{\mathrm{cz}} = 2\pi\int_{x_i}^{x_f} R\sqrt{1+R_x^2}\,dx = 2\pi\int_{x_i}^{x_f}\Big[&R_m - u + \frac{R_m}{2}u_x^2 - \frac{1}{2}uu_x^2 - \frac{R_m}{8}u_x^4 + \frac{1}{8}uu_x^4 \\
&+ \frac{R_m}{16}u_x^6 + \cdots\Big]dx. \text{ (S13)}
\end{aligned}
$$

$$A_{\mathrm{cz}}(s)/R_m^2 \approx 2\pi^2\left(\frac{6}{\Lambda}\right)^{1/4}\mathrm{s}^{1/2}+\frac{\pi^2 6^{1/4}\mathrm{s}^{3/2}}{288\Lambda^{5/4}}\left[(\Gamma-4)6^{3/2}\Lambda^{1/2}-72\Gamma-216\Lambda-6^{1/2}\Lambda^{3/2}\right]$$
$$+\frac{\pi^2 6^{1/4}\mathrm{s}^{5/2}}{55296\Lambda^{9/4}}\left[1841\Lambda^3+12006^{1/2}\Lambda^{5/2}-36(\Gamma-24C_0R_m+128)\Lambda^2\right.$$
$$-(7\Gamma+96C_0R_m+24)246^{1/2}\Lambda^{3/2}+(\Gamma^2+40\Gamma+576\,C_0R_m-848)36\Lambda$$
$$\left.-4326^{1/2}\Lambda^{1/2}\Gamma(\Gamma-4)+4320\Gamma^2\right]+\cdots.\ \text{(S14)}$$

$$V_{\mathrm{cz}}(s)/R_m^3 \approx \pi^2\left(\frac{6}{\Lambda}\right)^{1/4}\mathrm{s}^{1/2}+\frac{\pi^2 6^{1/4}\mathrm{s}^{3/2}}{576\Lambda^{5/4}}\left[(\Gamma-4)6^{3/2}\Lambda^{1/2}-72\Gamma-504\Lambda-256^{1/2}\Lambda^{3/2}\right]$$
$$+\frac{\pi^2 6^{1/4}\mathrm{s}^{5/2}}{110592\Lambda^{9/4}}\left[1505\Lambda^3+54006^{1/2}\Lambda^{5/2}+36(7\Gamma+24C_0R_m+800)\Lambda^2\right.$$
$$-(55\Gamma+96C_0R_m-72)246^{1/2}\Lambda^{3/2}+(\Gamma^2+232\Gamma+576\,C_0R_m-848)36\Lambda$$
$$\left.-4326^{1/2}\Lambda^{1/2}\Gamma(\Gamma-4)+4320\Gamma^2\right]+\cdots.\ \text{(S15)}$$

$$F_{\mathrm{cz}}(s)R_m/\kappa \approx \frac{2\pi^2 6^{1/4}\Lambda^{3/4}}{3s^{1/2}}-\frac{\pi^2 6^{1/4}\mathrm{s}^{1/2}}{96\Lambda^{3/4}}\left[56^{1/2}\Lambda^2+168\Lambda^{3/2}-(\Gamma-4)6^{3/2}\Lambda-72\Gamma\Lambda^{1/2}\right]$$
$$+\frac{5\pi^2 6^{1/4}\mathrm{s}^{3/2}}{55296\Lambda^{7/4}}\left[215\Lambda^{7/2}+10806^{1/2}\Lambda^3+(7\Gamma-24C_0R_m+800)12\Lambda^{5/2}\right.$$
$$-(55\Gamma+96C_0R_m-72)246^{1/2}\Lambda^2+(848-576C_0R_m-232\Gamma-\Gamma^2)36\Lambda^{3/2}$$
$$\left.+(\Gamma-4)1446^{1/2}\Gamma\Lambda-864\Gamma^2\Lambda^{1/2}\right]+\cdots.\ \text{(S16)}$$

3 Relation between forces exerted at maximum radius and constriction sites

The Canham-Helfrich hamiltonian gives the energy of a membrane vesicle with non-zero spontaneous curvature under the actions of surface tension and osmotic pressure difference between external and internal environments. However, the constriction process of a vesicle considered here requires additional contributions to the total energy of the vesicle. Keeping fixed R_m and R_c along the constriction pathway mean new constraints, which can be incorporated into the total energy of Eq. (4) via Lagrange multipliers $\lambda_m = -4\pi\sigma_m$ and $\lambda_c = 2\pi\sigma_c$, respectively

$$E_{T,\sigma} = E_T + 4\pi\sigma_m R_m + 2\pi\sigma_c R_c. \text{(S17)}$$

This equation leads to a new stable shape obtained from

$$\delta E_{T,\sigma} = \delta(E_T + 4\pi\sigma_m R_m + 2\pi\sigma_c R_c) = 0, \text{(S18)}$$

which leads to

$$\frac{\partial E_{T,\sigma}}{\partial R_c} = 0 \rightarrow F_c \equiv -\frac{\partial E_T}{\partial R_c} = 2\pi\sigma_{c,}, \text{(S19a)}$$
$$\frac{\partial E_{T,\sigma}}{\partial R_m} = 0 \rightarrow F_m \equiv -\frac{\partial E_T}{\partial R_m} = 4\pi\sigma_m\ , \text{(S19b)}$$

where F_c is the force exerted at the constriction site toward the interior, and F_m is the force exerted at the maximum radius sites toward the exterior. In general, $E_T = E_T(s, C_0 R_m, \Sigma R_m^2, \Delta p R_m^3)$, but in the case of zero spontaneous curvature ($C_0 = 0$), negligible surface tension ($\Sigma\ = 0$), and isotonic medium ($\Delta p\ = 0$) this energy, which corresponds to the bending energy of a membrane vesicle with $C_0 = 0$, only depends on the constriction parameter s. For this particular case, the Eq. (S19) gives

$$F_c = \frac{1}{R_m}\frac{\partial E_T}{\partial s} = 2\pi\sigma_{c,}, \text{(S20a)}$$
$$F_m \equiv -\frac{(1-s)}{R_m}\frac{\partial E_T}{\partial s} = 4\pi\sigma_m\ . \text{(S20b)}$$

From the Eq. (S20) we can extract the following relation between F_c and F_m during the constriction process for the case with $C_0 = \Sigma = \Delta p = 0$

$$F_c = -\frac{F_m}{(1-s)} = -\frac{4\pi\sigma_m}{1-s}, \text{(21)}$$

or equivalently, the following relation between σ_m and σ_c

$$\sigma_c = -\frac{2\sigma_m}{1-s}. \text{(S22)}$$

In the general case, when these parameters are non-zero, there is a similar relation between F_c and F_m, but it is more complicated due to the presence of the partial derivatives of E_T with respect to $C_0 R_m$, ΣR_m^2, and $\Delta p R_m^3$ in Eq. (S20b).

The constriction force computed from the line tensions σ_c and σ_m [Eqs. (S20a) and (S21)] and from the variations of E_T with respect to constriction radius $\partial E_T/\partial R_c$ as a function of the constriction ratio s for the case with $C_0 = \Sigma = \Delta p = 0$ is shown in Figure 10. We obtain an excellent agreement between the three possible ways of computing it. The constriction force computed from $-\partial E_T/\partial R_c$ has been calculated using the mean value theorem; this is the reason why these points are between those arising from the Eqs. (S20a) and (S21).

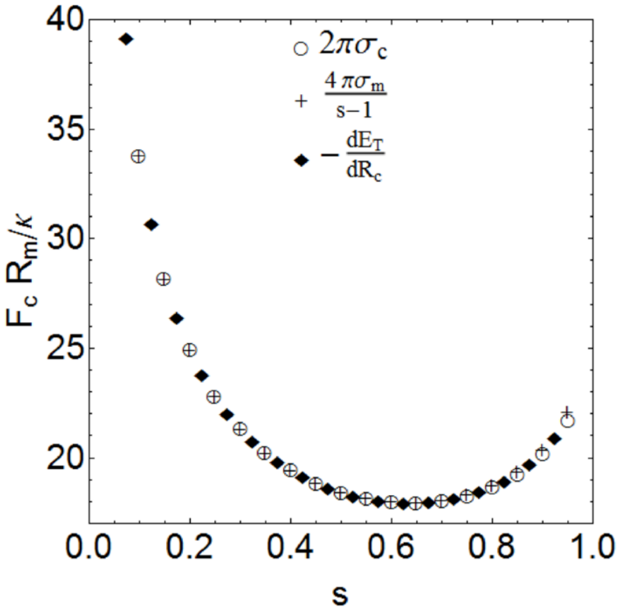

FigureS2. Constriction force (in units of R_m/κ) numerically computed for $C_0 = \Sigma = \Delta p = 0$. Plotted are $2\pi\sigma_c$ [Eq. (S20a)], where σ_c is the line tension at the constriction site (computed numerically), $-4\pi\,\sigma_m/(1-s)$ [Eq. (S21)], where σ_m is the line tension at the maximum polar radius site (computed numerically) and $-\partial E_T/\partial R_c$, calculated numerically from variations of the total energy along the constriction pathway. We obtain an excellent agreement between the three possible ways of computing the constriction force
