# Supplementary material for: Modeling the Mechanics of Cell Division: Influence of Spontaneous Membrane Curvature, Surface Tension, and Osmotic Pressure
Source: Front Physiol. 2017 May 19;8:312. doi: 10.3389/fphys.2017.00312 (PMC5437162; doi:10.3389/fphys.2017.00312)
Supplement: Supplementary file 2 [file DataSheet1.zip › Mathematica_Files/ANALYTICAL.pdf]

Supplementary File: Approximate analytical results for symmetric constriction

These *Mathematica* and pdf files are Supplementary Files of paper

E. Beltrán-Heredia, V. G. Almendro-Vedia, F. Monroy, and F. J. Cao,

Modelling the mechanics of cell division: Influence of spontaneous membrane curvature, surface tension and osmotic pressure. (2017)

doi: 10.3389/fphys.2017.00312

Below are the analytical expressions for poles and constriction zone up to sixth-order perturbative expansions (Section 2 of Supplementary Material).

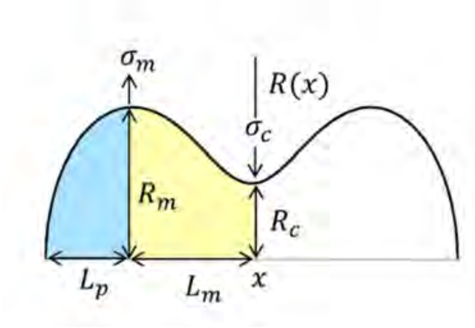

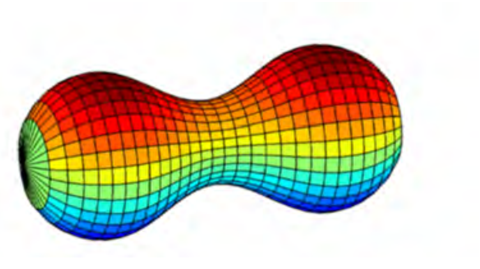

To load the functions of this notebook, execute NotebookEvaluate["C:\\Users\\Directory\\ANALYTICAL.nb"] where C:\\Users\\Directory\\ has to be replaced by the directory path where the file ANALYTICAL.nb is saved.

Results in Eqs. (24), (25) and (29)

```
G[sC0_, sbarsigma_] := (2 - sC0 ) ^2 + 2 sbarsigma - 1;
L[sC0_, sbarp_, sbarsigma_] := (1 - sC0) ^2 + 2 sbarsigma + sbarp;
eps[sC0_, sbarp_, sbarsigma_] := -5 (L[sC0, sbarp, sbarsigma] - 1) / (29 + G[sC0, sbarsigma]);
```

where

sC0 = $C_0 R_m$, sbarsigma = $\tilde{\Sigma} R_m^2 = \Sigma R_m^2 / \kappa$, and sbarp = $\tilde{\Delta \text{p}} R_m^3 = \Delta \text{p} R_m^3 / \kappa$.

Membrane area

Sum of the results in Eq. (16) (membrane area of the poles) and in Eq. (S14) (membrane area of the constriction zone).

```
AT[sC0_, sbarp_, sbarsigma_, s_] :=
```

$$4\pi\,\mathrm{Rm}^2 + \frac{8}{3}\,\mathrm{eps}[\mathrm{sC0},\mathrm{sbarp},\mathrm{sbarsigma}]\,\pi\,\mathrm{Rm}^2 + \frac{4}{15}\,\mathrm{eps}[\mathrm{sC0},\mathrm{sbarp},\mathrm{sbarsigma}]^2\,\pi\,\mathrm{Rm}^2 + \frac{2\times 6^{1/4}\,\pi^2\,\mathrm{Rm}^2\,\sqrt{s}}{L[\mathrm{sC0},\mathrm{sbarp},\mathrm{sbarsigma}]^{1/4}} - \frac{1}{48\times 6^{1/4}\,L[\mathrm{sC0},\mathrm{sbarp},\mathrm{sbarsigma}]^{5/4}}\,\pi^2 \Big(12\sqrt{6}\,G[\mathrm{sC0},\mathrm{sbarsigma}]\,\mathrm{Rm}^2 + 24\sqrt{L[\mathrm{sC0},\mathrm{sbarp},\mathrm{sbarsigma}]}\,\mathrm{Rm}^2 - 6\,G[\mathrm{sC0},\mathrm{sbarsigma}]\sqrt{L[\mathrm{sC0},\mathrm{sbarp},\mathrm{sbarsigma}]}\,\mathrm{Rm}^2 + 36\sqrt{6}\,L[\mathrm{sC0},\mathrm{sbarp},\mathrm{sbarsigma}]\,\mathrm{Rm}^2 + L[\mathrm{sC0},\mathrm{sbarp},\mathrm{sbarsigma}]^{3/2}\,\mathrm{Rm}^2\Big)\,s^{3/2} + \frac{1}{55\,296\,L[\mathrm{sC0},\mathrm{sbarp},\mathrm{sbarsigma}]^{9/4}}\,\pi^2 \Big(4320\times 6^{1/4}\,G[\mathrm{sC0},\mathrm{sbarsigma}]^2\,\mathrm{Rm}^2 + 1728\times 6^{3/4}\,G[\mathrm{sC0},\mathrm{sbarsigma}]\sqrt{L[\mathrm{sC0},\mathrm{sbarp},\mathrm{sbarsigma}]}\,\mathrm{Rm}^2 - 432\times 6^{3/4}\,G[\mathrm{sC0},\mathrm{sbarsigma}]^2\sqrt{L[\mathrm{sC0},\mathrm{sbarp},\mathrm{sbarsigma}]}\,\mathrm{Rm}^2 - 30\,528\times 6^{1/4}\,L[\mathrm{sC0},\mathrm{sbarp},\mathrm{sbarsigma}]\,\mathrm{Rm}^2 + 1440\times 6^{1/4}\,G[\mathrm{sC0},\mathrm{sbarsigma}]\,L[\mathrm{sC0},\mathrm{sbarp},\mathrm{sbarsigma}]\,\mathrm{Rm}^2 + 36\times 6^{1/4}\,G[\mathrm{sC0},\mathrm{sbarsigma}]^2\,L[\mathrm{sC0},\mathrm{sbarp},\mathrm{sbarsigma}]\,\mathrm{Rm}^2 - 576\times 6^{3/4}\,L[\mathrm{sC0},\mathrm{sbarp},\mathrm{sbarsigma}]^{3/2}\,\mathrm{Rm}^2 - 168\times 6^{3/4}\,G[\mathrm{sC0},\mathrm{sbarsigma}]\,L[\mathrm{sC0},\mathrm{sbarp},\mathrm{sbarsigma}]^{3/2}\,\mathrm{Rm}^2 - 4608\times 6^{1/4}\,L[\mathrm{sC0},\mathrm{sbarp},\mathrm{sbarsigma}]^2\,\mathrm{Rm}^2 - 36\times 6^{1/4}\,G[\mathrm{sC0},\mathrm{sbarsigma}]\,L[\mathrm{sC0},\mathrm{sbarp},\mathrm{sbarsigma}]^2\,\mathrm{Rm}^2 + 120\times 6^{3/4}\,L[\mathrm{sC0},\mathrm{sbarp},\mathrm{sbarsigma}]^{5/2}\,\mathrm{Rm}^2 + 1841\times 6^{1/4}\,L[\mathrm{sC0},\mathrm{sbarp},\mathrm{sbarsigma}]^3\,\mathrm{Rm}^2 + 20\,736\times 6^{1/4}\,\mathrm{sC0}\,L[\mathrm{sC0},\mathrm{sbarp},\mathrm{sbarsigma}]\,\mathrm{Rm}^2 - 2304\times 6^{3/4}\,\mathrm{sC0}\,L[\mathrm{sC0},\mathrm{sbarp},\mathrm{sbarsigma}]^{3/2}\,\mathrm{Rm}^2 + 864\times 6^{1/4}\,\mathrm{sC0}\,L[\mathrm{sC0},\mathrm{sbarp},\mathrm{sbarsigma}]^2\,\mathrm{Rm}^2\Big)\,s^{5/2}$$

Volume enclosed

Sum of the results in Eq. (17) (volume enclosed by the poles) and in Eq. (S15) (volume enclosed by the constriction zone).

```
VT[sC0_, sbarp_, sbarsigma_, s_] :=
```

$$\frac{4\pi \text{Rm}^3}{3}+\frac{4}{3}\text{eps}[\text{sC0},\text{sbarp},\text{sbarsigma}]\,\pi\,\text{Rm}^3+\frac{6^{1/4}\pi^2\text{Rm}^3\sqrt{s}}{L[\text{sC0},\text{sbarp},\text{sbarsigma}]^{1/4}}-$$

$$\frac{1}{576\,L[\text{sC0},\text{sbarp},\text{sbarsigma}]^{5/4}}\Big(72\times 6^{1/4}G[\text{sC0},\text{sbarsigma}]+24\times 6^{3/4}\sqrt{L[\text{sC0},\text{sbarp},\text{sbarsigma}]}-$$

$$6\times 6^{3/4}G[\text{sC0},\text{sbarsigma}]\sqrt{L[\text{sC0},\text{sbarp},\text{sbarsigma}]}+504\times 6^{1/4}L[\text{sC0},\text{sbarp},\text{sbarsigma}]+$$

$$25\times 6^{3/4}L[\text{sC0},\text{sbarp},\text{sbarsigma}]^{3/2}\Big)\pi^2\,\text{Rm}^3\,s^{3/2}+$$

$$\frac{1}{110592\,L[\text{sC0},\text{sbarp},\text{sbarsigma}]^{9/4}}\pi^2\,\text{Rm}^3$$

$$\Big(4320\times 6^{1/4}G[\text{sC0},\text{sbarsigma}]^2+1728\times 6^{3/4}G[\text{sC0},\text{sbarsigma}]\sqrt{L[\text{sC0},\text{sbarp},\text{sbarsigma}]}-$$

$$432\times 6^{3/4}G[\text{sC0},\text{sbarsigma}]^2\sqrt{L[\text{sC0},\text{sbarp},\text{sbarsigma}]}-$$

$$30528\times 6^{1/4}L[\text{sC0},\text{sbarp},\text{sbarsigma}]+$$

$$8352\times 6^{1/4}G[\text{sC0},\text{sbarsigma}]\,L[\text{sC0},\text{sbarp},\text{sbarsigma}]+$$

$$36\times 6^{1/4}G[\text{sC0},\text{sbarsigma}]^2\,L[\text{sC0},\text{sbarp},\text{sbarsigma}]+$$

$$1728\times 6^{3/4}L[\text{sC0},\text{sbarp},\text{sbarsigma}]^{3/2}-$$

$$1320\times 6^{3/4}G[\text{sC0},\text{sbarsigma}]\,L[\text{sC0},\text{sbarp},\text{sbarsigma}]^{3/2}+$$

$$28800\times 6^{1/4}L[\text{sC0},\text{sbarp},\text{sbarsigma}]^2+$$

$$252\times 6^{1/4}G[\text{sC0},\text{sbarsigma}]\,L[\text{sC0},\text{sbarp},\text{sbarsigma}]^2+$$

$$5400\times 6^{3/4}L[\text{sC0},\text{sbarp},\text{sbarsigma}]^{5/2}+1505\times 6^{1/4}L[\text{sC0},\text{sbarp},\text{sbarsigma}]^3+$$

$$20736\times 6^{1/4}\,\text{sC0}\,L[\text{sC0},\text{sbarp},\text{sbarsigma}]-2304\times 6^{3/4}\,\text{sC0}\,L[\text{sC0},\text{sbarp},\text{sbarsigma}]^{3/2}+$$

$$864\times 6^{1/4}\,\text{sC0}\,L[\text{sC0},\text{sbarp},\text{sbarsigma}]^2\Big)s^{5/2}$$

Total Energy

Sum of the results in Eq. (27) (total energy of the poles) and in Eq. (S12) (total energy of the constriction zone).

```
ET[sC0_, sbarp_, sbarsigma_, s_] :=
 2/15 eps[sC0, sbarp, sbarsigma]^2 (29 + G[sC0, sbarsigma]) k π +
  4/3 eps[sC0, sbarp, sbarsigma] k (-1 + L[sC0, sbarp, sbarsigma]) π +
  2/3 k π (7 + G[sC0, sbarsigma] + 2 L[sC0, sbarp, sbarsigma] - 4 sC0) +
  (4 × 2^(1/4) k L[sC0, sbarp, sbarsigma]^(3/4) π^2 Sqrt[s]) / 3^(3/4) -
  1/(144 L[sC0, sbarp, sbarsigma]^(1/4)) k
   (-72 6^(1/4) G[sC0, sbarsigma] + 24 × 6^(3/4) Sqrt[L[sC0, sbarp, sbarsigma]] -
     6 × 6^(3/4) G[sC0, sbarsigma] Sqrt[L[sC0, sbarp, sbarsigma]] + 168 × 6^(1/4) L[sC0, sbarp, sbarsigma] +
     5 × 6^(3/4) L[sC0, sbarp, sbarsigma]^(3/2)) π^2 s^(3/2) -
  1/(27 648 L[sC0, sbarp, sbarsigma]^(5/4)) k π^2
   (864 × 6^(1/4) G[sC0, sbarsigma]^2 + 576 × 6^(3/4) G[sC0, sbarsigma] Sqrt[L[sC0, sbarp, sbarsigma]] -
     144 × 6^(3/4) G[sC0, sbarsigma]^2 Sqrt[L[sC0, sbarp, sbarsigma]] -
     30 528 × 6^(1/4) L[sC0, sbarp, sbarsigma] +
     8352 × 6^(1/4) G[sC0, sbarsigma] L[sC0, sbarp, sbarsigma] +
     36 × 6^(1/4) G[sC0, sbarsigma]^2 L[sC0, sbarp, sbarsigma] -
     1728 × 6^(3/4) L[sC0, sbarp, sbarsigma]^(3/2) +
     1320 × 6^(3/4) G[sC0, sbarsigma] L[sC0, sbarp, sbarsigma]^(3/2) -
     9600 × 6^(1/4) L[sC0, sbarp, sbarsigma]^2 - 84 × 6^(1/4) G[sC0, sbarsigma] L[sC0, sbarp, sbarsigma]^2 -
     1080 × 6^(3/4) L[sC0, sbarp, sbarsigma]^(5/2) - 215 × 6^(1/4) L[sC0, sbarp, sbarsigma]^3 +
     20 736 × 6^(1/4) sC0 L[sC0, sbarp, sbarsigma] + 2304 × 6^(3/4) sC0 L[sC0, sbarp, sbarsigma]^(3/2) -
     288 × 6^(1/4) sC0 L[sC0, sbarp, sbarsigma]^2) s^(5/2)
```

Polar Distance

Polar distance [results in Eq. (15) and (29)].

```
Lp[sC0_, sbarp_, sbarsigma_, s_] := (1 + eps[sC0, sbarp, sbarsigma]) Rm
```

Constriction Length

Constriction length [result in Eq. (S11)].

$$\mathrm{Lm}[\mathrm{sC0\_},\ \mathrm{sbarp\_},\ \mathrm{sbarsigma\_},\ \mathrm{s\_}] := \frac{3^{1/4}\,\pi\,\mathrm{Rm}\,\sqrt{\mathrm{s}}}{2^{3/4}\,\mathrm{L}[\mathrm{sC0},\ \mathrm{sbarp},\ \mathrm{sbarsigma}]^{1/4}} + \frac{1}{192\times 6^{1/4}\,\mathrm{L}[\mathrm{sC0},\ \mathrm{sbarp},\ \mathrm{sbarsigma}]^{5/4}} \Big(6\,\mathrm{G}[\mathrm{sC0},\ \mathrm{sbarsigma}]\left(-2\sqrt{6} + \sqrt{\mathrm{L}[\mathrm{sC0},\ \mathrm{sbarp},\ \mathrm{sbarsigma}]}\right) - 24\sqrt{\mathrm{L}[\mathrm{sC0},\ \mathrm{sbarp},\ \mathrm{sbarsigma}]} + 12\sqrt{6}\,\mathrm{L}[\mathrm{sC0},\ \mathrm{sbarp},\ \mathrm{sbarsigma}] - 25\,\mathrm{L}[\mathrm{sC0},\ \mathrm{sbarp},\ \mathrm{sbarsigma}]^{3/2}\Big)\,\pi\,\mathrm{Rm}\,\mathrm{s}^{3/2} + \frac{1}{36\,864\times 6^{3/4}\,\mathrm{L}[\mathrm{sC0},\ \mathrm{sbarp},\ \mathrm{sbarsigma}]^{9/4}}\,\pi\,\mathrm{Rm} \Big(36\,\mathrm{G}[\mathrm{sC0},\ \mathrm{sbarsigma}]^2 \left(120 - 12\sqrt{6}\sqrt{\mathrm{L}[\mathrm{sC0},\ \mathrm{sbarp},\ \mathrm{sbarsigma}]} + \mathrm{L}[\mathrm{sC0},\ \mathrm{sbarp},\ \mathrm{sbarsigma}]\right) - 12\,\mathrm{G}[\mathrm{sC0},\ \mathrm{sbarsigma}] \left(-144\sqrt{6}\sqrt{\mathrm{L}[\mathrm{sC0},\ \mathrm{sbarp},\ \mathrm{sbarsigma}]} + 456\,\mathrm{L}[\mathrm{sC0},\ \mathrm{sbarp},\ \mathrm{sbarsigma}] + 14\sqrt{6}\,\mathrm{L}[\mathrm{sC0},\ \mathrm{sbarp},\ \mathrm{sbarsigma}]^{3/2} - 21\,\mathrm{L}[\mathrm{sC0},\ \mathrm{sbarp},\ \mathrm{sbarsigma}]^2\right) + \mathrm{L}[\mathrm{sC0},\ \mathrm{sbarp},\ \mathrm{sbarsigma}] \Big(600\sqrt{6}\,\mathrm{L}[\mathrm{sC0},\ \mathrm{sbarp},\ \mathrm{sbarsigma}]^{3/2} + 1505\,\mathrm{L}[\mathrm{sC0},\ \mathrm{sbarp},\ \mathrm{sbarsigma}]^2 + 288\,\mathrm{L}[\mathrm{sC0},\ \mathrm{sbarp},\ \mathrm{sbarsigma}]\,(4 + 3\,\mathrm{sC0}) - 576\sqrt{6}\sqrt{\mathrm{L}[\mathrm{sC0},\ \mathrm{sbarp},\ \mathrm{sbarsigma}]}\,(5 + 4\,\mathrm{sC0}) + 576\,(-53 + 36\,\mathrm{sC0})\Big)\Big)\,\mathrm{s}^{5/2};$$

Constriction Force

Constriction force [result in Eq. (S16)].

$$\mathrm{FC}[\mathrm{sC0\_},\ \mathrm{sbarp\_},\ \mathrm{sbarsigma\_},\ \mathrm{s\_}] := \frac{2\times 2^{1/4}\,\mathrm{k}\,\mathrm{L}[\mathrm{sC0},\ \mathrm{sbarp},\ \mathrm{sbarsigma}]^{3/4}\,\pi^2}{3^{3/4}\,\mathrm{Rm}\,\sqrt{\mathrm{s}}} - \frac{1}{16\left(6^{3/4}\,\mathrm{L}[\mathrm{sC0},\ \mathrm{sbarp},\ \mathrm{sbarsigma}]^{1/4}\,\mathrm{Rm}\right)} \Big(\mathrm{k}\Big(-72\,\mathrm{G}[\mathrm{sC0},\ \mathrm{sbarsigma}] + 24\sqrt{6}\sqrt{\mathrm{L}[\mathrm{sC0},\ \mathrm{sbarp},\ \mathrm{sbarsigma}]} - 6\sqrt{6}\,\mathrm{G}[\mathrm{sC0},\ \mathrm{sbarsigma}]\sqrt{\mathrm{L}[\mathrm{sC0},\ \mathrm{sbarp},\ \mathrm{sbarsigma}]} + 168\,\mathrm{L}[\mathrm{sC0},\ \mathrm{sbarp},\ \mathrm{sbarsigma}] + 5\sqrt{6}\,\mathrm{L}[\mathrm{sC0},\ \mathrm{sbarp},\ \mathrm{sbarsigma}]^{3/2}\Big)\,\pi^2\Big)\sqrt{\mathrm{s}} + \frac{1}{9216\times 6^{3/4}\,\mathrm{L}[\mathrm{sC0},\ \mathrm{sbarp},\ \mathrm{sbarsigma}]^{5/4}\,\mathrm{Rm}}\,\mathrm{k}\,\pi^2 \Big(-4320\,\mathrm{G}[\mathrm{sC0},\ \mathrm{sbarsigma}]^2 - 2880\sqrt{6}\,\mathrm{G}[\mathrm{sC0},\ \mathrm{sbarsigma}]\sqrt{\mathrm{L}[\mathrm{sC0},\ \mathrm{sbarp},\ \mathrm{sbarsigma}]} + 720\sqrt{6}\,\mathrm{G}[\mathrm{sC0},\ \mathrm{sbarsigma}]^2\sqrt{\mathrm{L}[\mathrm{sC0},\ \mathrm{sbarp},\ \mathrm{sbarsigma}]} + 152\,640\,\mathrm{L}[\mathrm{sC0},\ \mathrm{sbarp},\ \mathrm{sbarsigma}] - 41\,760\,\mathrm{G}[\mathrm{sC0},\ \mathrm{sbarsigma}]\,\mathrm{L}[\mathrm{sC0},\ \mathrm{sbarp},\ \mathrm{sbarsigma}] - 180\,\mathrm{G}[\mathrm{sC0},\ \mathrm{sbarsigma}]^2\,\mathrm{L}[\mathrm{sC0},\ \mathrm{sbarp},\ \mathrm{sbarsigma}] + 8640\sqrt{6}\,\mathrm{L}[\mathrm{sC0},\ \mathrm{sbarp},\ \mathrm{sbarsigma}]^{3/2} - 6600\sqrt{6}\,\mathrm{G}[\mathrm{sC0},\ \mathrm{sbarsigma}]\,\mathrm{L}[\mathrm{sC0},\ \mathrm{sbarp},\ \mathrm{sbarsigma}]^{3/2} + 48\,000\,\mathrm{L}[\mathrm{sC0},\ \mathrm{sbarp},\ \mathrm{sbarsigma}]^2 + 420\,\mathrm{G}[\mathrm{sC0},\ \mathrm{sbarsigma}]\,\mathrm{L}[\mathrm{sC0},\ \mathrm{sbarp},\ \mathrm{sbarsigma}]^2 + 5400\sqrt{6}\,\mathrm{L}[\mathrm{sC0},\ \mathrm{sbarp},\ \mathrm{sbarsigma}]^{5/2} + 1075\,\mathrm{L}[\mathrm{sC0},\ \mathrm{sbarp},\ \mathrm{sbarsigma}]^3 - 103\,680\,\mathrm{sC0}\,\mathrm{L}[\mathrm{sC0},\ \mathrm{sbarp},\ \mathrm{sbarsigma}] - 11\,520\sqrt{6}\,\mathrm{sC0}\,\mathrm{L}[\mathrm{sC0},\ \mathrm{sbarp},\ \mathrm{sbarsigma}]^{3/2} + 1440\,\mathrm{sC0}\,\mathrm{L}[\mathrm{sC0},\ \mathrm{sbarp},\ \mathrm{sbarsigma}]^2\Big)\,\mathrm{s}^{3/2};$$

Some Results

Analysis of the surface tension and osmotic pressure

The values $\tilde{\Sigma} R_m^2 = \pm 0.3$ and $\Delta \tilde{p} R_m^3 = \pm 0.3$ used correspond to $\Sigma = \pm 1.2 \times 10^{-8}$ N / m and $\Delta p = \pm 1.2 \times 10^{-2}$ N / m^2 for a cell-sized artificial vesicle ($R_m = 1$ μm).

```
PlotFC5 =
 Rasterize[Plot[{Expand[FC[0, 0, 0.3, s] Rm / k], Expand[FC[0, 0.3, 0, s] Rm / k],
    Expand[FC[0, 0, 0, s] Rm / k], Expand[FC[0, -0.3, 0, s] Rm / k],
    Expand[FC[0, 0, -0.3, s] Rm / k]}, {s, 0, 1},
   PlotStyle → {{Blue, Thick}, {Red, Thick}, {Black, Thick}, {Orange, Thick}, {Cyan, Thick}},
   PlotRange → {{0, 1}, Automatic},
   FrameLabel → {{Style["FcRm/κ", SingleLetterItalics → False], ""}, {"s", Style["", Bold]}},
   LabelStyle → Directive[FontSize → 30, FontFamily → "Helvetica"], ImageSize → 560,
   AspectRatio → 1, Frame → True,
   PlotLegends → Placed[{Style["Δp̃R³m=0, Σ̃R²m=0.3", 30, "Helvetica"],
      Style["Δp̃R³m=0.3, Σ̃R²m=0", 30, "Helvetica"], Style["Δp̃R³m=0, Σ̃R²m=0", 30, "Helvetica"],
      Style["Δp̃R³m=-0.3, Σ̃R²m=0", 30, "Helvetica"],
      Style["Δp̃R³m=0, Σ̃R²m=-0.3", 30, "Helvetica"]}, {{Right, Top}}]]]
```

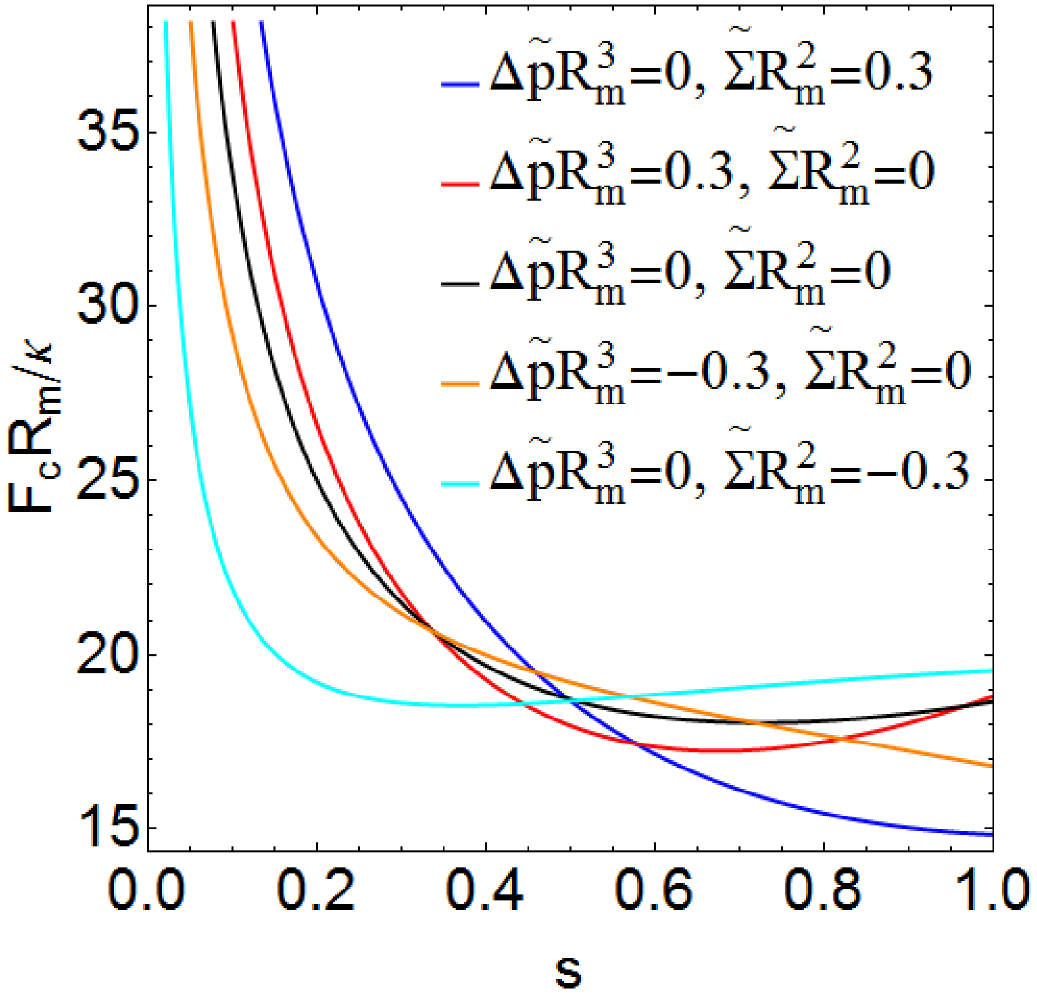

```
PlotEnergy5 =
 Rasterize[
  Plot[{ET[0, 0, 0.3, s] / (8 Pi k), ET[0, 0.3, 0, s] / (8 Pi k), ET[0, 0, 0, s] / (8 Pi k),
    ET[0, -0.3, 0, s] / (8 Pi k), ET[0, 0, -0.3, s] / (8 Pi k)}, {s, 0, 1},
   PlotStyle → {{Blue, Thick}, {Red, Thick}, {Black, Thick}, {Orange, Thick}, {Cyan, Thick}},
   PlotRange → {{0, 1}, Automatic},
   FrameLabel → {{Style["ET/(8πκ)", SingleLetterItalics → False], ""},
     {"s", Style["", Bold]}},
   LabelStyle → Directive[FontSize → 30, FontFamily → "Helvetica"], ImageSize → 560,
   AspectRatio → 1, Frame → True]]
```

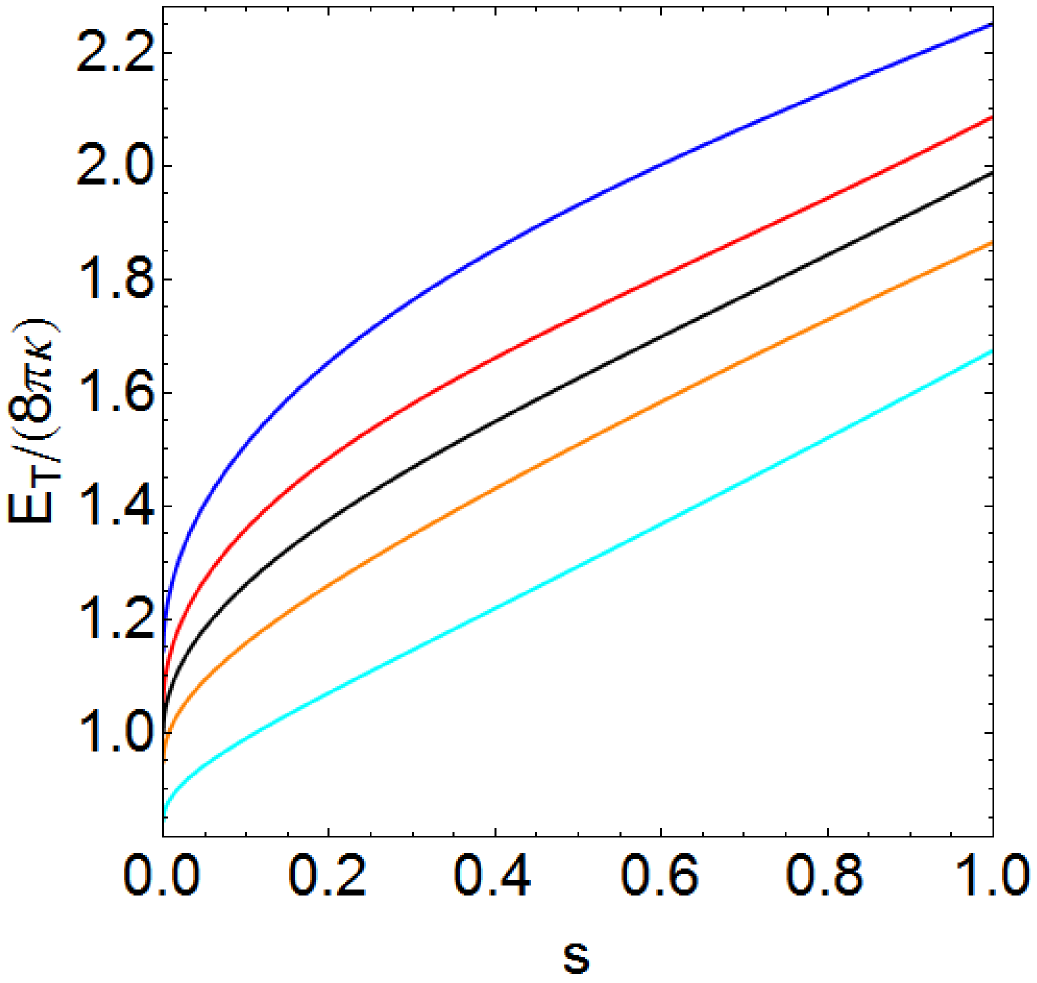

```
PlotArea5 =
 Rasterize[
  Plot[{Expand[AT[0, 0, 0.3, s] / (4 Pi Rm^2)], Expand[AT[0, 0.3, 0, s] / (4 Pi Rm^2)],
    Expand[AT[0, 0, 0, s] / (4 Pi Rm^2)], Expand[AT[0, -0.3, 0, s] / (4 Pi Rm^2)],
    Expand[AT[0, 0, -0.3, s] / (4 Pi Rm^2)]}, {s, 0, 1},
   PlotStyle → {{Blue, Thick}, {Red, Thick}, {Black, Thick}, {Orange, Thick}, {Cyan, Thick}},
   PlotRange → {{0, 1}, Automatic},
   FrameLabel → {{Style["A/(4πRm^2)", SingleLetterItalics → False], ""},
     {"s", Style["", Bold]}},
   LabelStyle → Directive[FontSize → 30, FontFamily → "Helvetica"], ImageSize → 560,
   AspectRatio → 1, Frame → True]]
```

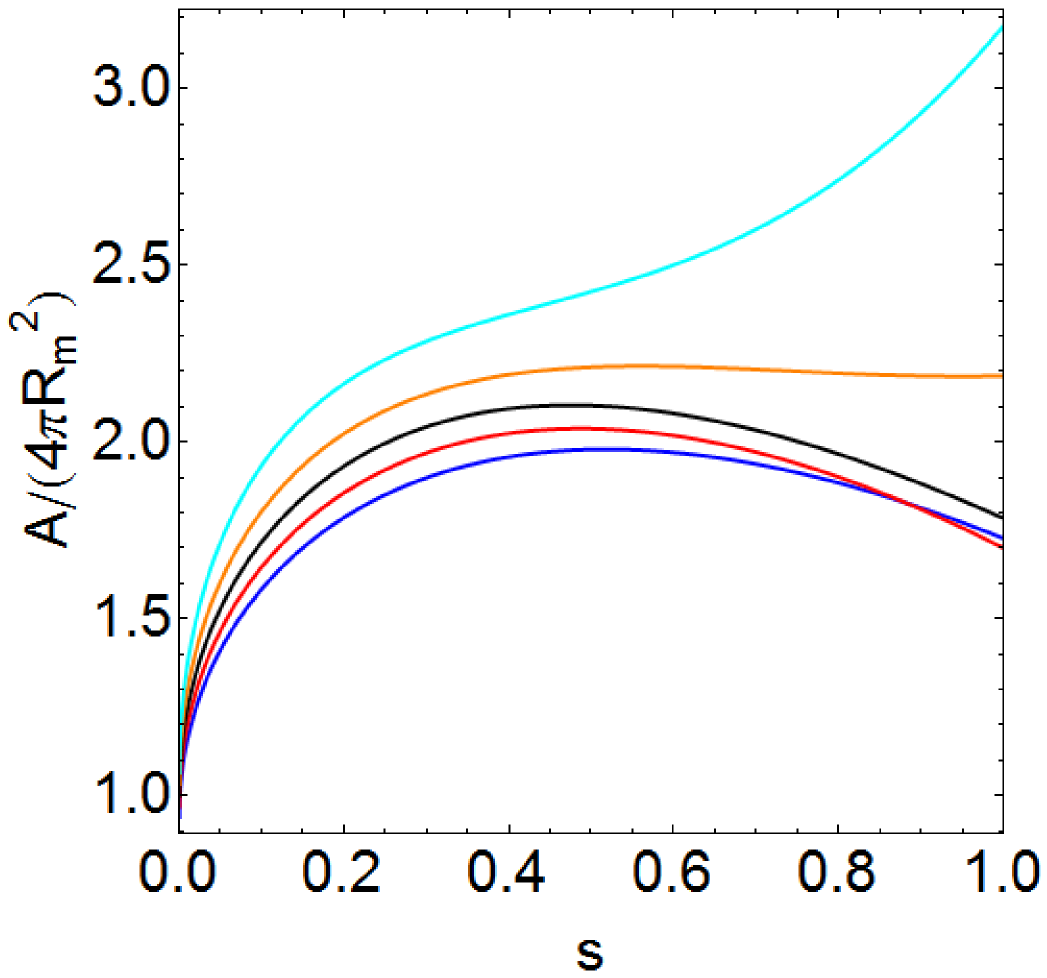

```
PlotVolume5 =
 Rasterize[
  Plot[{Expand[VT[0, 0, 0.3, s] / (4 Pi / 3 Rm^3)], Expand[VT[0, 0.3, 0, s] / (4 Pi / 3 Rm^3)],
    Expand[VT[0, 0, 0, s] / (4 Pi  / 3 Rm^3)], Expand[VT[0, -0.3, 0, s] / (4 Pi / 3 Rm^3)],
    Expand[VT[0, 0, -0.3, s] / (4 Pi  / 3 Rm^3)]}, {s, 0, 1},
   PlotStyle → {{Blue, Thick}, {Red, Thick}, {Black, Thick}, {Orange, Thick}, {Cyan, Thick}},
   PlotRange → {{0, 1}, Automatic},
   FrameLabel → {{Style["V/(4π/3Rm^3)", SingleLetterItalics → False], ""},
     {"s", Style["", Bold]}},
   LabelStyle → Directive[FontSize → 30, FontFamily →  "Helvetica"], ImageSize → 560,
   AspectRatio → 1, Frame → True]]
```

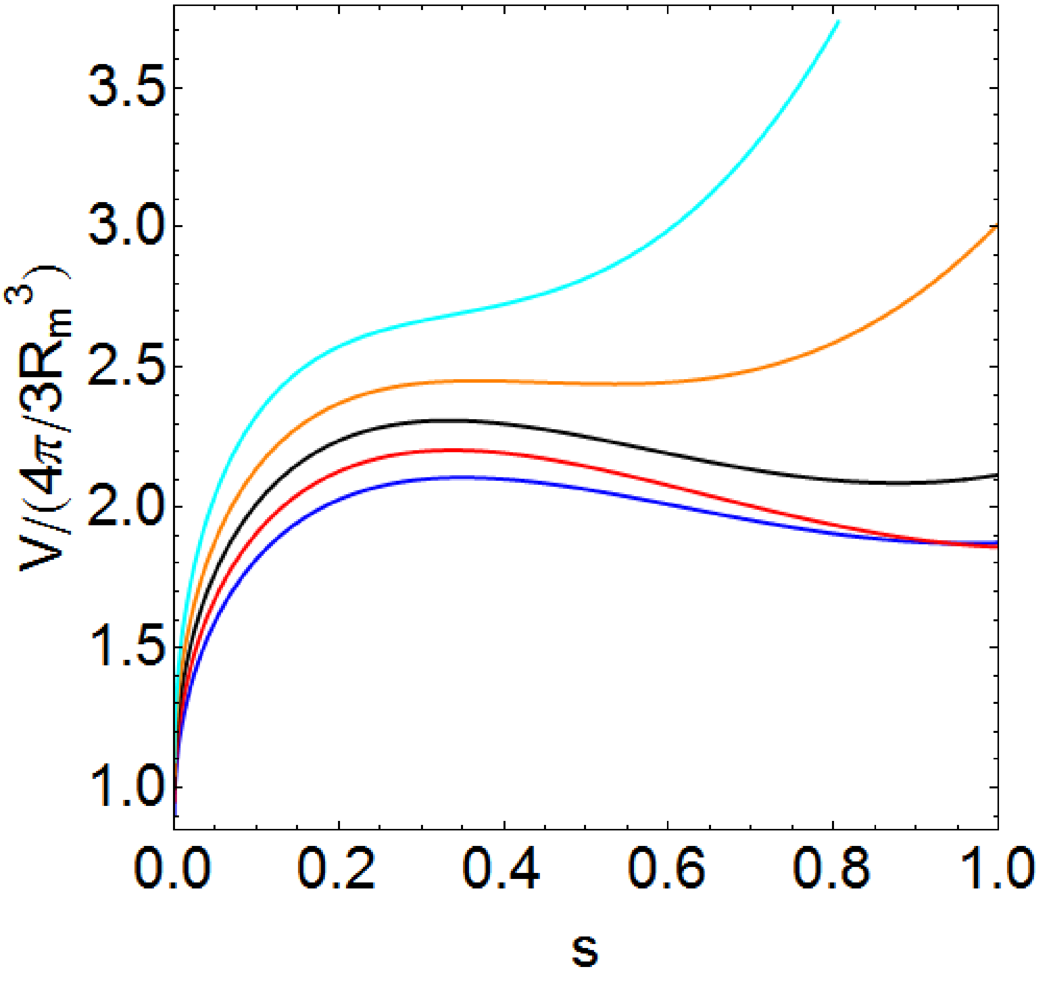

```
PlotLm5 =
 Rasterize[Plot[{Expand[Lm[0, 0, 0.3, s] / Rm], Expand[Lm[0, 0.3, 0, s] / Rm],
     Expand[Lm[0, 0, 0, s] / Rm], Expand[Lm[0, -0.3, 0, s] / Rm], Expand[Lm[0, 0, -0.3, s] / Rm]},
   {s, 0, 1}, PlotStyle → {{Blue, Thick}, {Red, Thick}, {Black, Thick}, {Orange, Thick},
     {Cyan, Thick}}, PlotRange → {{0, 1}, Automatic},
   FrameLabel → {{Style["Lm/Rm", SingleLetterItalics → False], ""}, {"s", Style["", Bold]}},
   LabelStyle → Directive[FontSize → 30, FontFamily → "Helvetica"], ImageSize → 560,
   AspectRatio → 1, Frame → True]]
```

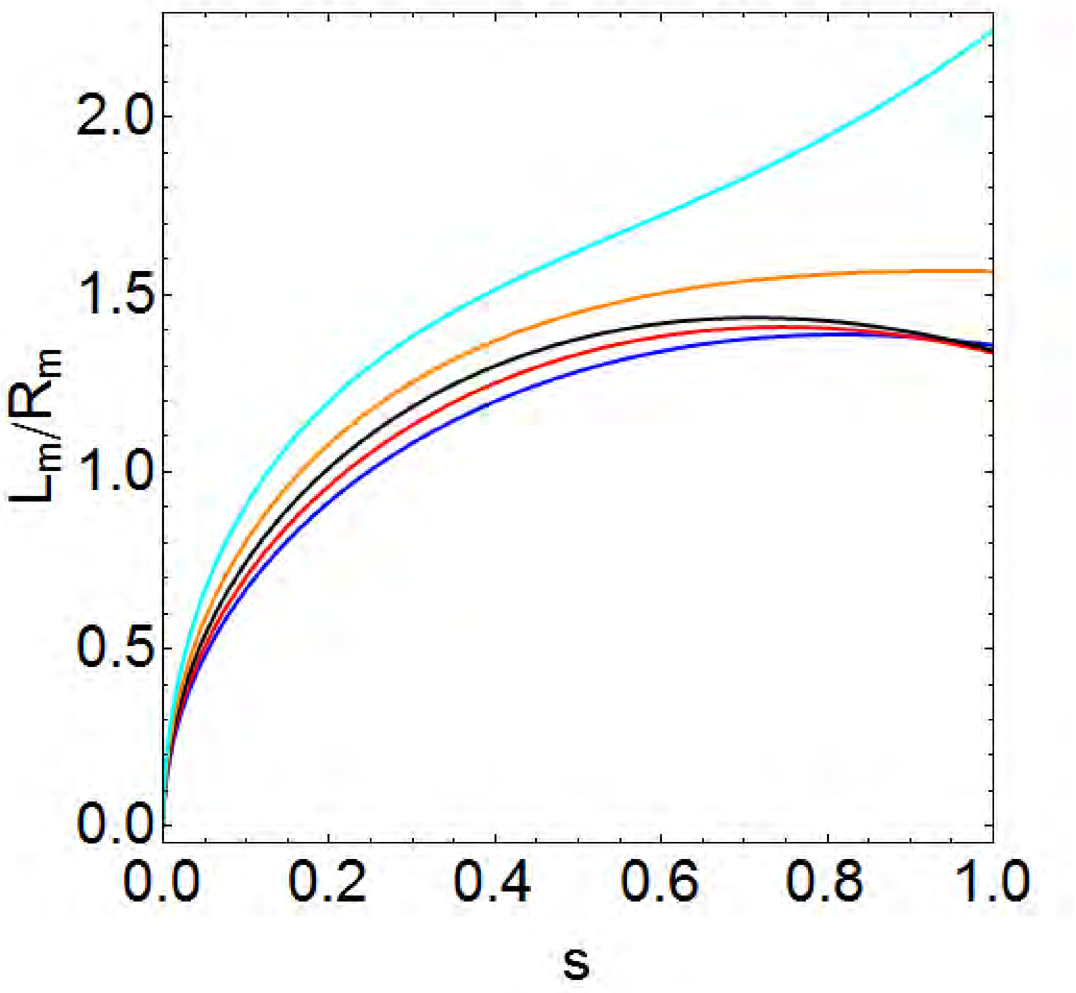

```
PlotLp5 =
 Rasterize[Plot[{Expand[Lp[0, 0, 0.3, s] / Rm], Expand[Lp[0, 0.3, 0, s] / Rm],
    Expand[Lp[0, 0, 0, s] / Rm], Expand[Lp[0, -0.3, 0, s] / Rm], Expand[Lp[0, 0, -0.3, s] / Rm]},
   {s, 0, 1}, PlotStyle → {{Blue, Thick}, {Red, Thick}, {Black, Thick}, {Orange, Thick},
     {Cyan, Thick}}, PlotRange → {{0, 1}, Automatic},
   FrameLabel → {{Style["Lp/Rm", SingleLetterItalics → False], ""}, {"s", Style["", Bold]}},
   LabelStyle → Directive[FontSize → 30, FontFamily → "Helvetica"], ImageSize → 560,
   AspectRatio → 1, Frame → True]]
```

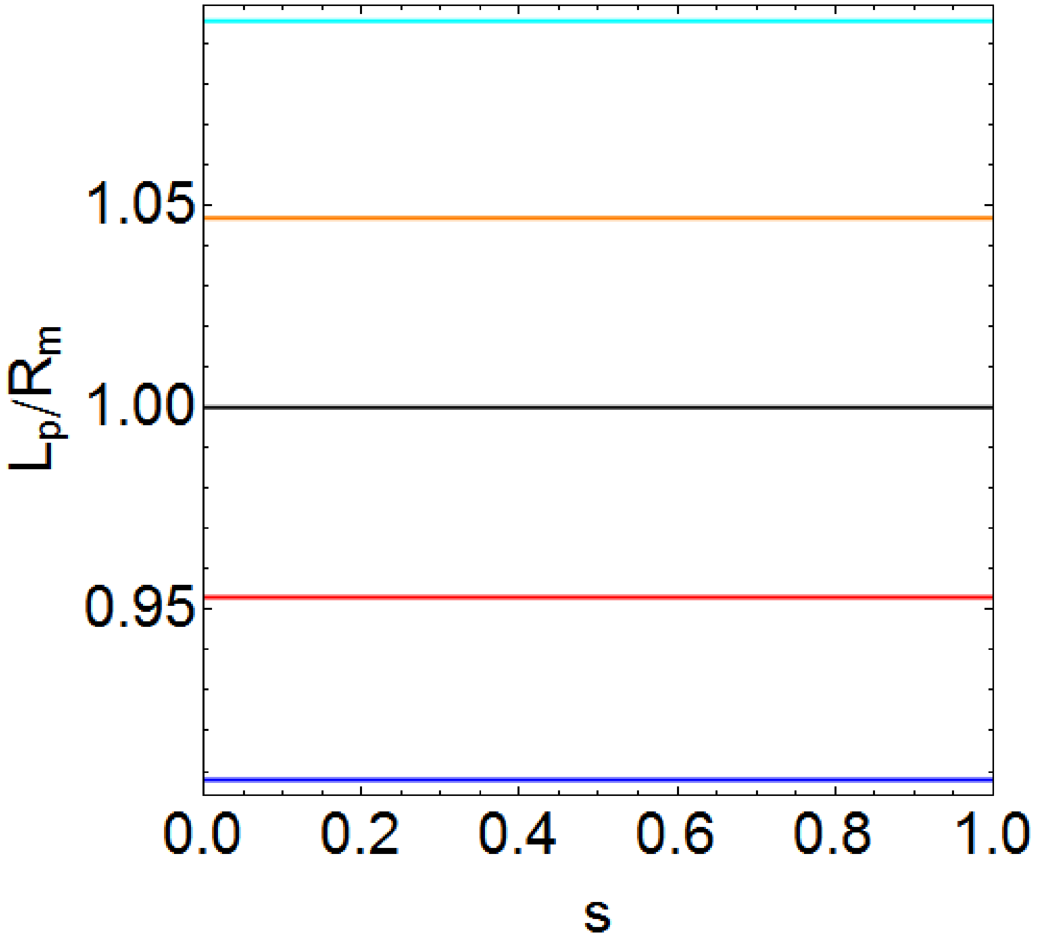

Analysis of the spontaneous curvature

The values $C_0 R_m = \pm 0.3$ used correspond to $C_0 R_m = \pm 0.3\,\mu m$ for a cell-sized artificial vesicle ($R_m = 1\,\mu m$).

```
PlotFC3 =
 Rasterize[Plot[{Expand[FC[-0.3, 0, 0, s] Rm / k], Expand[FC[0, 0, 0, s] Rm / k],
    Expand[FC[0.3, 0, 0, s] Rm / k]}, {s, 0, 1},
   PlotStyle → {{Brown, Thick}, {Black, Thick}, {Green, Thick}},
   PlotRange → {{0, 1}, Automatic},
   FrameLabel → {{Style["FcRm/κ", SingleLetterItalics → False], ""}, {"s", Style["", Bold]}},
   LabelStyle → Directive[FontSize → 30, FontFamily → "Helvetica"], ImageSize → 560,
   AspectRatio → 1, Frame → True,
   PlotLegends →
    Placed[{Style["C0Rm=-0.3", 30, "Helvetica"], Style["C0Rm=0", 30, "Helvetica"],
      Style["C0Rm=0.3", 30, "Helvetica"]}, {{Right, Top}}]]]
```

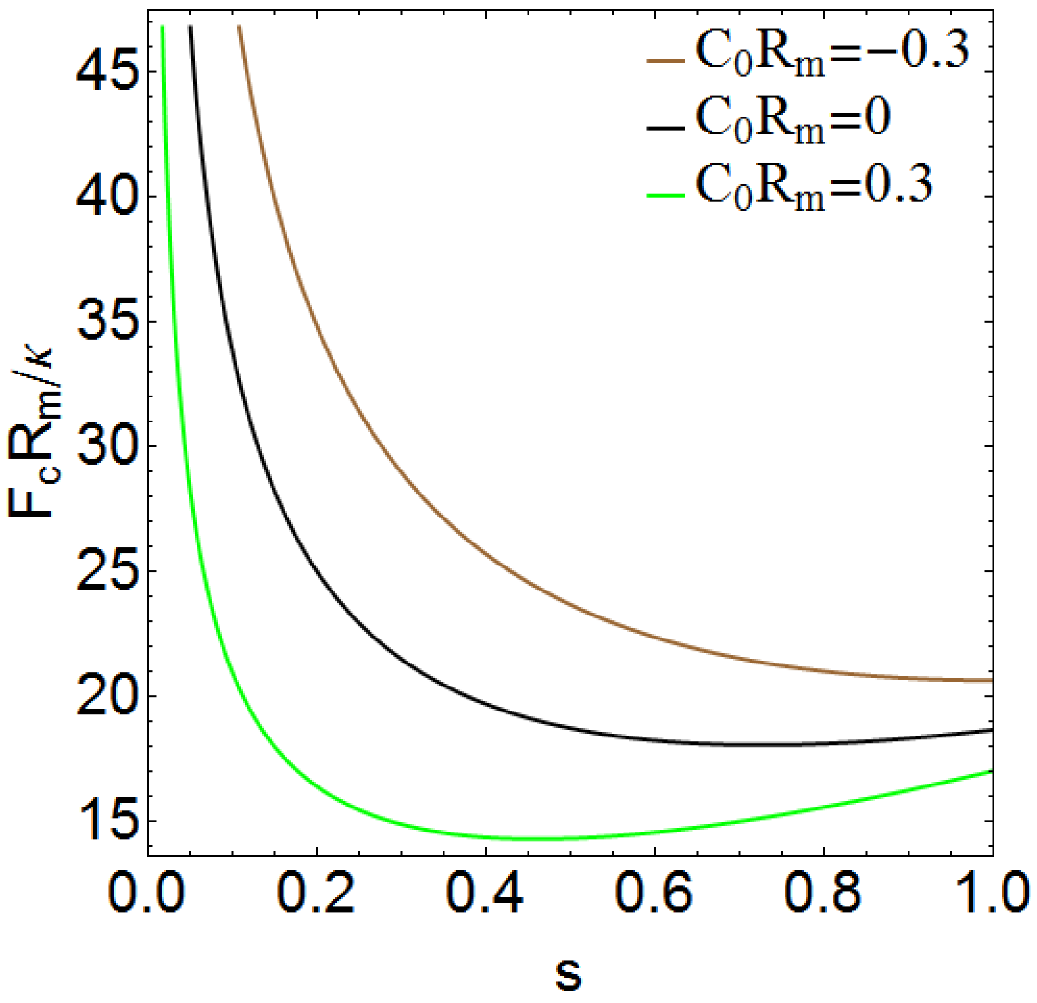

```
PlotEnergy3 =
 Rasterize[
  Plot[{ET[-0.3, 0, 0, s] / (8 Pi k), ET[0, 0, 0, s] / (8 Pi k), ET[0.3, 0, 0, s] / (8 Pi k)},
   {s, 0, 1}, PlotStyle → {{Brown, Thick}, {Black, Thick}, {Green, Thick}},
   PlotRange → {{0, 1}, Automatic},
   FrameLabel → {{Style["ET/(8πκ)", SingleLetterItalics → False], ""},
     {"s", Style["", Bold]}},
   LabelStyle → Directive[FontSize → 30, FontFamily → "Helvetica"], ImageSize → 560,
   AspectRatio → 1, Frame → True]]
```

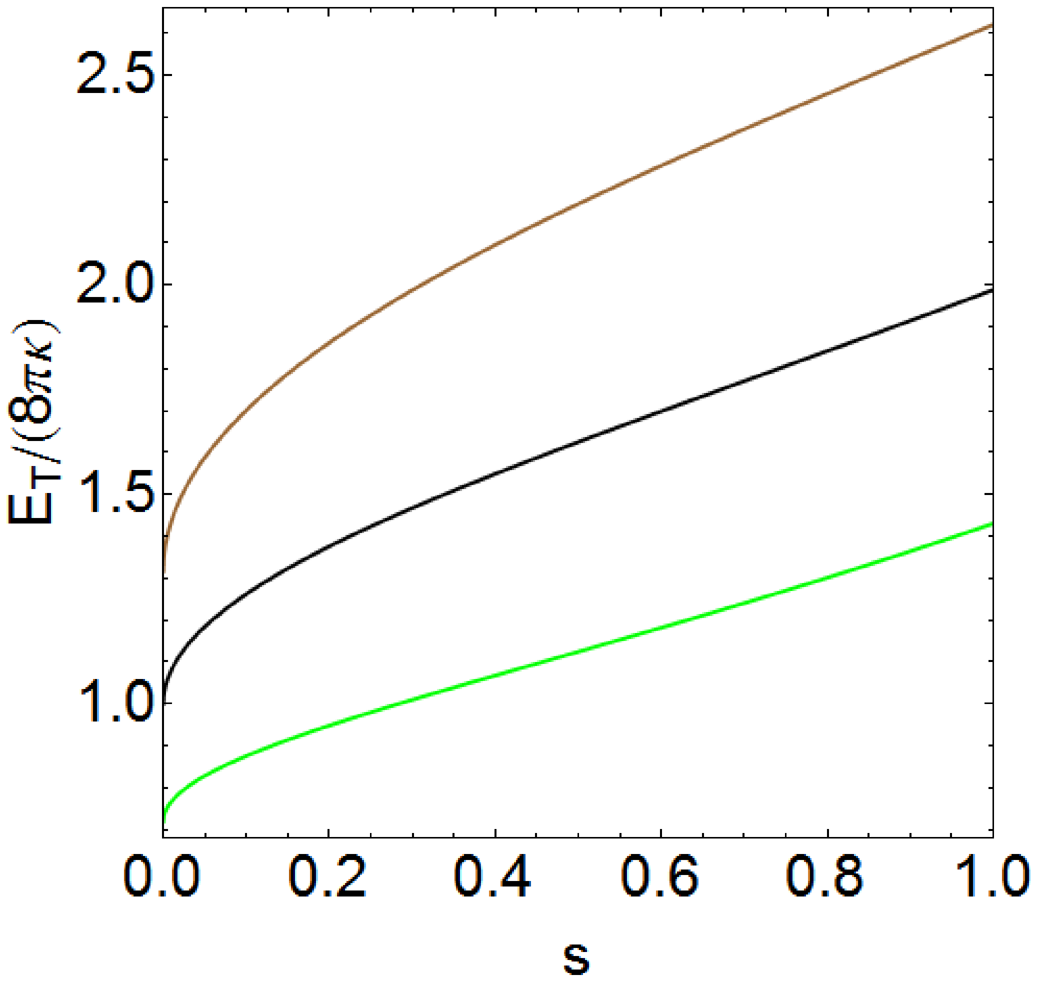

```
PlotArea3 =
 Rasterize[
  Plot[{Expand[AT[0.3, 0, 0, s] / (4 Pi Rm^2)], Expand[AT[0, 0, 0, s] / (4 Pi Rm^2)],
    Expand[AT[-0.3, 0, 0, s] / (4 Pi Rm^2)]}, {s, 0, 1},
   PlotStyle → {{Brown, Thick}, {Black, Thick}, {Green, Thick}},
   PlotRange → {{0, 1}, Automatic},
   FrameLabel → {{Style["A/(4πRm²)", SingleLetterItalics → False], ""},
     {"s", Style["", Bold]}},
   LabelStyle → Directive[FontSize → 30, FontFamily → "Helvetica"], ImageSize → 560,
   AspectRatio → 1, Frame → True]]
```

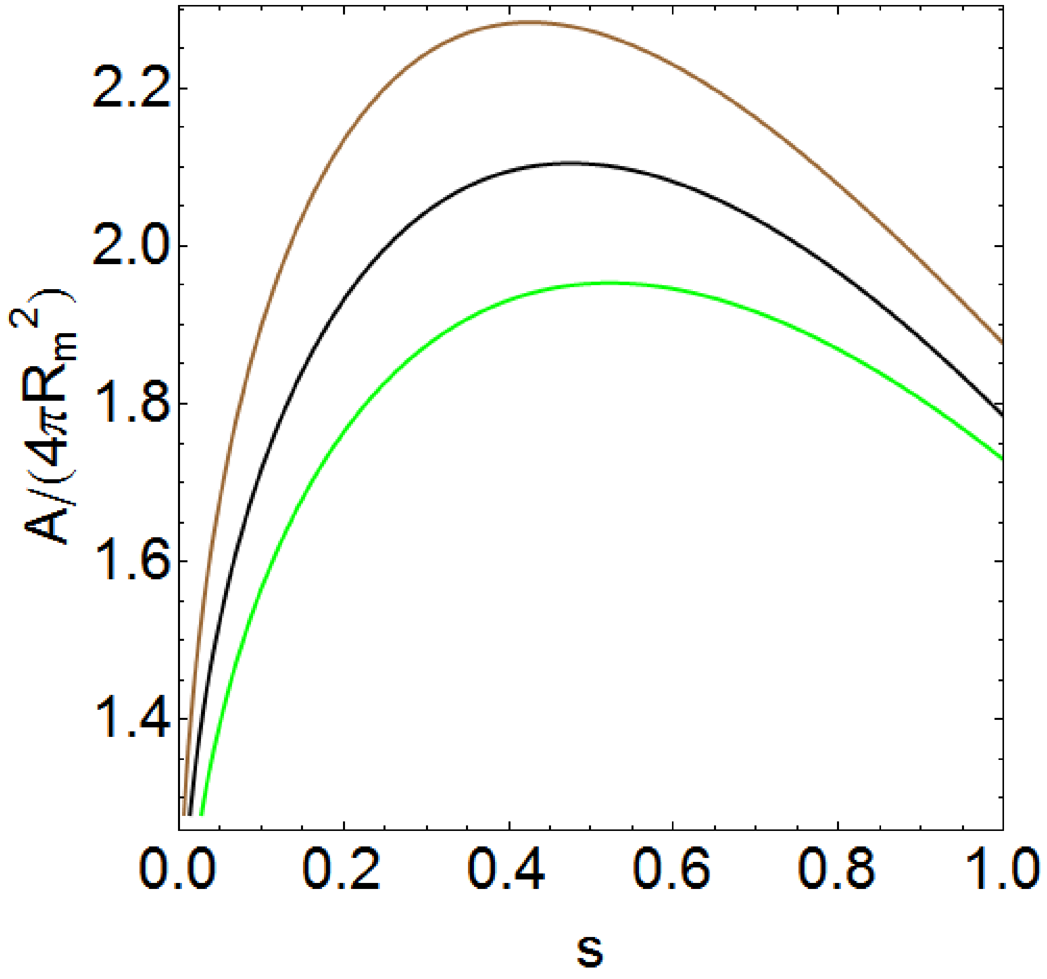

```
PlotVolume3 =
 Rasterize[
  Plot[{Expand[VT[0.3, 0, 0, s] / (4 Pi / 3 Rm^3)], Expand[VT[0, 0, 0, s] / (4 Pi / 3 Rm^3)],
     Expand[VT[-0.3, 0, 0, s] / (4 Pi / 3 Rm^3)]}, {s, 0, 1},
   PlotStyle → {{Brown, Thick}, {Black, Thick}, {Green, Thick}},
   PlotRange → {{0, 1}, Automatic},
   FrameLabel → {{Style["V/(4π/3Rm^3)", SingleLetterItalics → False], ""},
     {"s", Style["", Bold]}},
   LabelStyle → Directive[FontSize → 30, FontFamily → "Helvetica"], ImageSize → 560,
   AspectRatio → 1, Frame → True]]
```

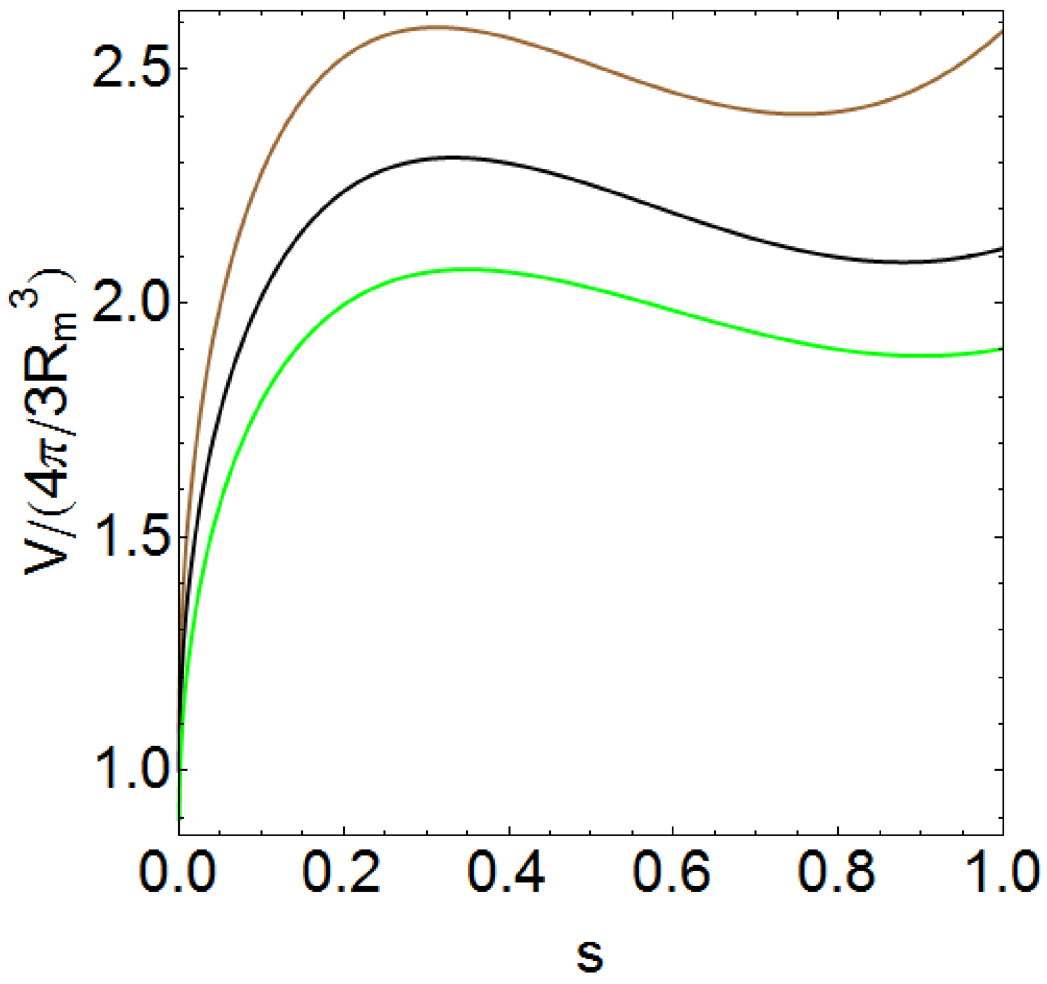

```
PlotLm3 =
 Rasterize[Plot[{Expand[Lm[-0.3, 0, 0, s] / Rm], Expand[Lm[0, 0, 0, s] / Rm],
    Expand[Lm[0.3, 0, 0, s] / Rm]}, {s, 0, 1},
   PlotStyle → {{Brown, Thick}, {Black, Thick}, {Green, Thick}},
   PlotRange → {{0, 1}, Automatic},
   FrameLabel → {{Style["Lm/Rm", SingleLetterItalics → False], ""}, {"s", Style["", Bold]}},
   LabelStyle → Directive[FontSize → 30, FontFamily → "Helvetica"], ImageSize → 560,
   AspectRatio → 1, Frame → True]]
```

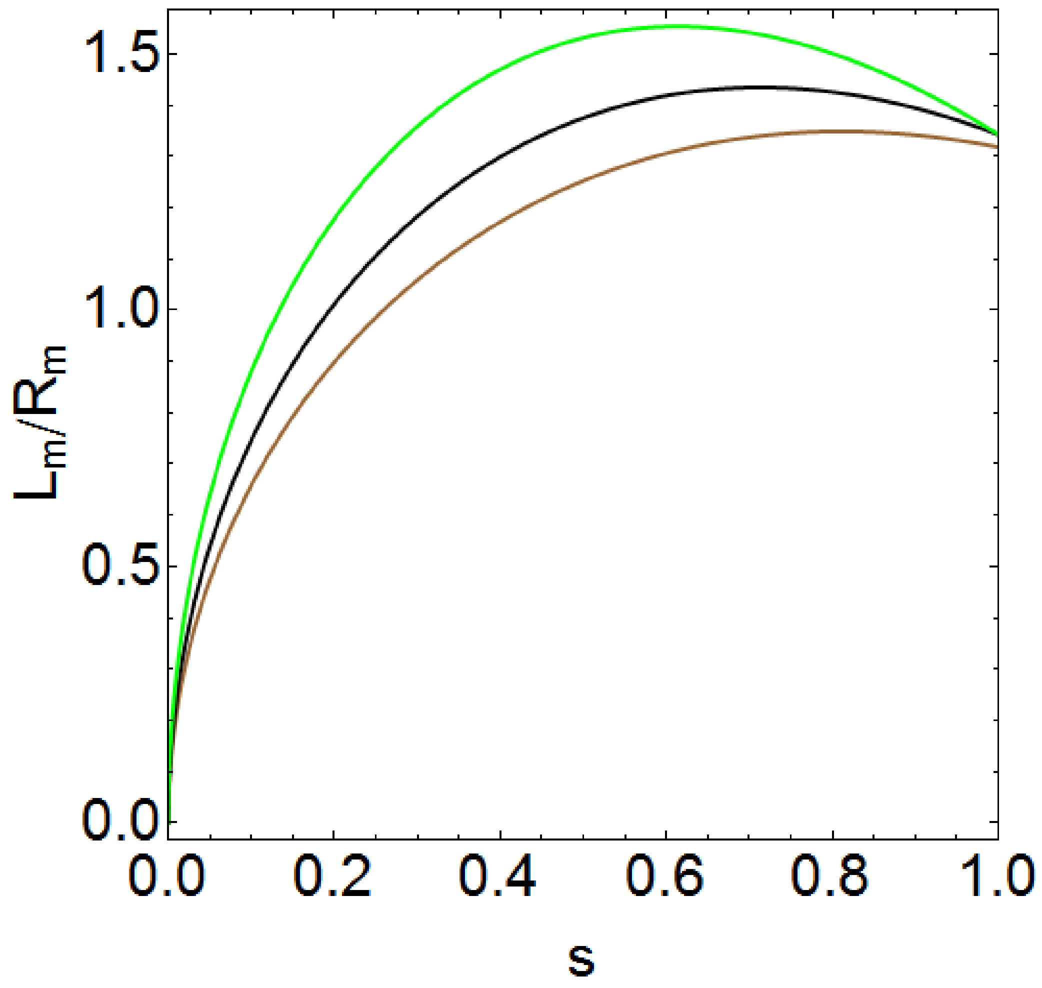

```
PlotLp3 =
 Rasterize[Plot[{Expand[Lp[-0.3, 0, 0, s] / Rm], Expand[Lp[0, 0, 0, s] / Rm],
    Expand[Lp[0.3, 0, 0, s] / Rm]}, {s, 0, 1},
   PlotStyle → {{Brown, Thick}, {Black, Thick}, {Green, Thick}},
   PlotRange → {{0, 1}, Automatic},
   FrameLabel → {{Style["Lp/Rm", SingleLetterItalics → False], ""}, {"s", Style["", Bold]}},
   LabelStyle → Directive[FontSize → 30, FontFamily → "Helvetica"], ImageSize → 560,
   AspectRatio → 1, Frame → True]]
```

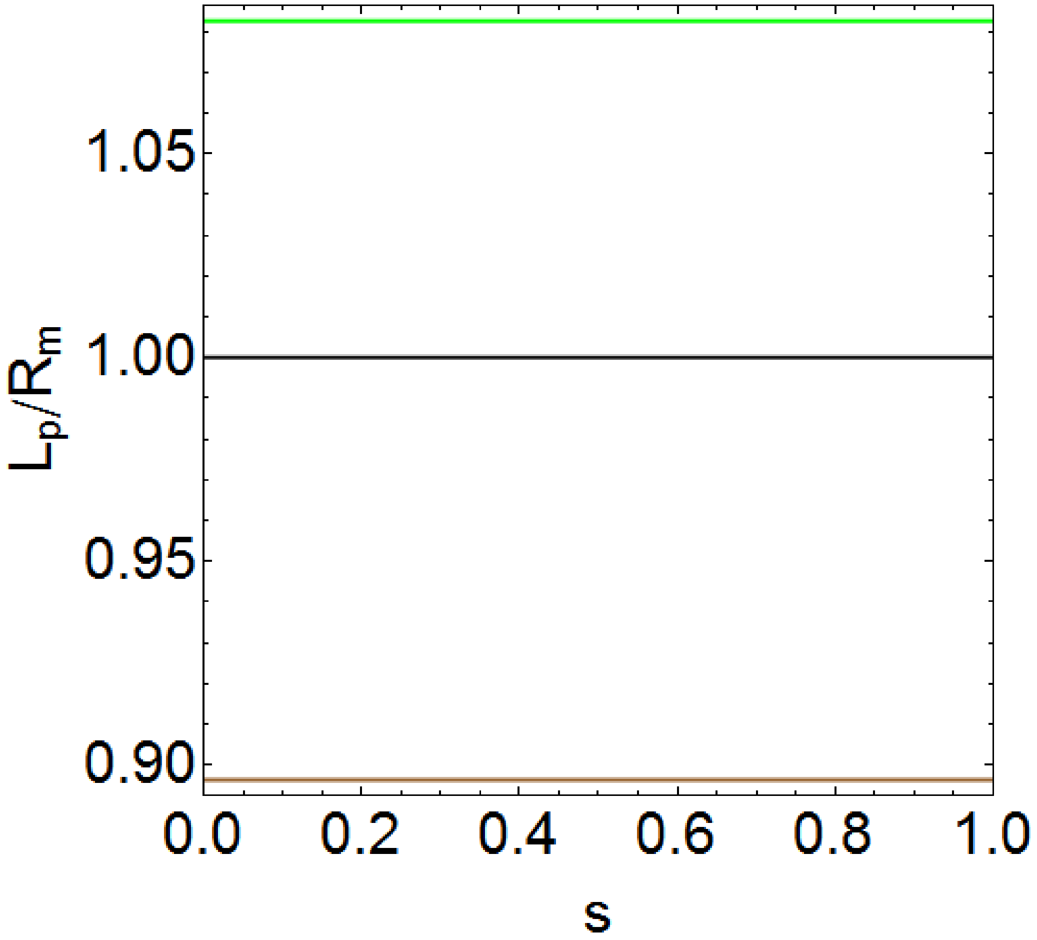

References

[1] Almendro-Vedia, V.G., Monroy, F., and Cao, F.J. (2015). Analytical results for cell constriction dominated by bending energy. *Phys. Rev. E*, **91**, 012713.

[2] Almendro-Vedia, V.G., Monroy, F., and Cao, F.J. (2013). Mechanics of Constriction during Cell Division: A Variational Approach. *PLoS One*, **8**, e69750.

[3] Beltran-Heredia, E., Almendro-Vedia, V.G., Monroy, F., and Cao, F.J. (2017). Modelling the mechanics of cell division: influence of spontaneous curvature, surface tension, and osmotic pressure. *Front. Physio.*
