# Supplementary material for: Modeling the Mechanics of Cell Division: Influence of Spontaneous Membrane Curvature, Surface Tension, and Osmotic Pressure
Source: Front Physiol. 2017 May 19;8:312. doi: 10.3389/fphys.2017.00312 (PMC5437162; doi:10.3389/fphys.2017.00312)
Supplement: Supplementary file 2 [file DataSheet1.zip › Mathematica_Files/DEDUCTION.pdf]

Supplementary File: Deduction of the approximate analytical results for symmetric constriction

These *Mathematica* and pdf files are Supplementary Files of paper

E. Beltrán-Heredia, V. G. Almendro-Vedia, F. Monroy, and F. J. Cao,

Modelling the mechanics of cell division: Influence of spontaneous membrane curvature, surface tension and osmotic pressure. (2017)

doi: 10.3389/fphys.2017.00312

Below is the deduction of the analytical expressions for poles and constriction zone up to sixth-order perturbative expansions.

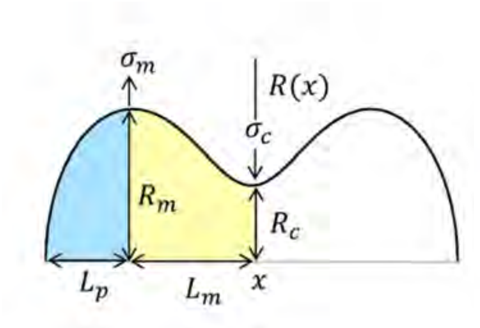

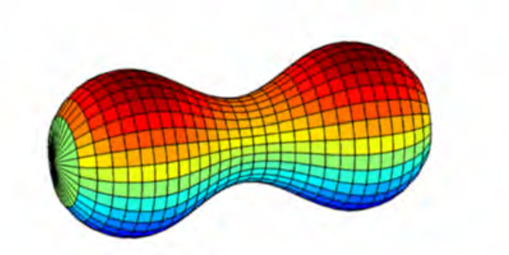

To load the functions of this notebook, execute NotebookEvaluate["C:\\Users\\Directory\\DEDUCTION.nb"] where C:\\Users\\Directory\\ has to be replaced by the directory path where the file DEDUCTION.nb is saved.

Polar caps zone

Profile of the polar caps zone [Eqs. (13) and (14)]. As the left and the right polar caps are identical (due to the central symmetry assumed), we have considered the right one and then multiply the quantities by a factor 2.

```
Lp = (1 + eps) Rm;

Rcap = Rm (1 - x^2 / Lp^2) ^ (1 / 2);
```

Membrane Area

Integrand of the membrane area of the poles up to third-order in ϵ [Eq. (8)].

```
Apolesserie = Series[4 Pi Rcap (1 + D[Rcap, x] ^2) ^ (1 / 2), {eps, 0, 3}];
```

Simplification of the coefficients.

```
Apolesserieorder0 = Assuming[{Rm^2 ≥ x^2, Rm > 0},
   Simplify[SeriesCoefficient[Apolesserie, 0]]];
Apolesserieorder1 = Assuming[{Rm^2 ≥ x^2, Rm > 0},
   Simplify[SeriesCoefficient[Apolesserie, 1]]];
Apolesserieorder2 = Assuming[{Rm^2 ≥ x^2, Rm > 0},
   Simplify[SeriesCoefficient[Apolesserie, 2]]];
Apolesserieorder3 = Assuming[{Rm^2 ≥ x^2, Rm > 0},
   Simplify[SeriesCoefficient[Apolesserie, 3]]];
```

Integration between $x = 0$ and $x = L_p$ [Eq. (11)].

```
Apolesorder0 = Integrate[Apolesserieorder0, {x, 0, Lp}];
Apolesorder1 = Integrate[Apolesserieorder1, {x, 0, Lp}];
Apolesorder2 = Integrate[Apolesserieorder2, {x, 0, Lp}];
Apolesorder3 = Integrate[Apolesserieorder3, {x, 0, Lp}];
```

Area of the poles [result in Eq. (16)].

```
Apoles = Series[Apolesorder0 + Apolesorder1 eps + Apolesorder2 eps^2 + Apolesorder3 eps^3,
  {eps, 0, 2}]
```

$$4\pi \mathrm{Rm}^2 + \frac{8}{3}\pi \mathrm{Rm}^2\,\mathrm{eps} + \frac{4}{15}\pi \mathrm{Rm}^2\,\mathrm{eps}^2 + O[\mathrm{eps}]^3$$

Volume enclosed

Integrand of the volume enclosed by the poles up to third-order in ϵ [Eq. (9)].

```
Vpolesserie = Series[2 Pi Rcap^2, {eps, 0, 3}];
```

Simplification of the coefficients.

```
Vpolesserieorder0 = Assuming[{Rm^2 ≥ x^2, Rm > 0},
   Simplify[SeriesCoefficient[Vpolesserie, 0]]];
Vpolesserieorder1 = Assuming[{Rm^2 ≥ x^2, Rm > 0},
   Simplify[SeriesCoefficient[Vpolesserie, 1]]];
Vpolesserieorder2 = Assuming[{Rm^2 ≥ x^2, Rm > 0},
   Simplify[SeriesCoefficient[Vpolesserie, 2]]];
Vpolesserieorder3 = Assuming[{Rm^2 ≥ x^2, Rm > 0},
   Simplify[SeriesCoefficient[Vpolesserie, 3]]];
```

Integration between $x = 0$ and $x = L_p$ [Eq. (11)].

```
Vpolesorder0 = Integrate[Vpolesserieorder0, {x, 0, Lp}];
Vpolesorder1 = Integrate[Vpolesserieorder1, {x, 0, Lp}];
Vpolesorder2 = Integrate[Vpolesserieorder2, {x, 0, Lp}];
Vpolesorder3 = Integrate[Vpolesserieorder3, {x, 0, Lp}];
```

Volume of the poles up to second-order in ϵ [result in Eq. (17)].

```
Vpoles = Series[Vpolesorder0 + Vpolesorder1 eps + Vpolesorder2 eps^2 + Vpolesorder3 eps^3,
  {eps, 0, 2}]
```

$$\frac{4\pi \mathrm{Rm}^3}{3} + \frac{4}{3}\pi \mathrm{Rm}^3\,\mathrm{eps} + O[\mathrm{eps}]^3$$

Total Energy

Integrand of the energy of the poles up to third-order in ϵ [Eqs. (4), (6) and (7)].

```
Epolesserie =
  Series[
   2 Pi k (1 + D[Rcap, x]^2 - D[D[Rcap, x], x] Rcap - Rcap C0 (1 + D[Rcap, x]^2)^(3/2))^2 / Rcap /
      (1 + D[Rcap, x]^2)^(5/2) + 2 Pi barp k Rcap^2 +
    4 Pi barsigma k Rcap (1 + D[Rcap, x]^2)^(1/2), {eps, 0, 3}];
```

Simplification of the coefficients.

```
Epolesserieorder0 = Assuming[{Rm^2 ≥ x^2, Rm > 0},
   Simplify[SeriesCoefficient[Epolesserie, 0]]];
Epolesserieorder1 = Assuming[{Rm^2 ≥ x^2, Rm > 0},
   Simplify[SeriesCoefficient[Epolesserie, 1]]];
Epolesserieorder2 = Assuming[{Rm^2 ≥ x^2, Rm > 0},
   Simplify[SeriesCoefficient[Epolesserie, 2]]];
Epolesserieorder3 = Assuming[{Rm^2 ≥ x^2, Rm > 0},
   Simplify[SeriesCoefficient[Epolesserie, 3]]];
```

Integration between $x = 0$ and $x = L_p$ [Eq. (11)].

```
Epolesorder0 = Integrate[Epolesserieorder0, {x, 0, Lp}];
Epolesorder1 = Integrate[Epolesserieorder1, {x, 0, Lp}];
Epolesorder2 = Integrate[Epolesserieorder2, {x, 0, Lp}];
Epolesorder3 = Integrate[Epolesserieorder3, {x, 0, Lp}];
```

Total energy of the poles up to second-order in ϵ [result in Eq. (27)].

```
Epoles =
 Series[
  Simplify[Epolesorder0 + Epolesorder1 eps + Epolesorder2 eps^2 + Epolesorder3 eps^3 /.
     barp → (L - 1 - C0^2 Rm^2 + 2 C0 Rm - 2 barsigma Rm^2) / (Rm^3) /.
    barsigma → (G - 4 + 1 - C0^2 Rm^2 + 4 C0 Rm) / (2 Rm^2)], {eps, 0, 2}]
```

$$\frac{2}{3} k \pi (7 + G + 2 L - 4 C0 Rm) + \frac{4}{3} k (-1 + L) \pi eps + \frac{2}{15} (29 + G) k \pi eps^2 + O[eps]^3$$

Polar Distance

Minimization of the total energy. Result in Eq. (29).

```
eps = epsx /. Solve[D[Normal[Series[Epoles /. eps → epsx, {epsx, 0, 2}]], epsx] == 0, epsx][[1]]
```

$$-\frac{5 (-1 + L)}{29 + G}$$

Polar distance.

```
Lp
```

$$\left(1 - \frac{5 (-1 + L)}{29 + G}\right) Rm$$

Constriction zone

Profile of the polar caps zone [Eqs. (7), (19), and (20)]. As the left and the right polar caps are identical (due to the central symmetry assumed), we have considered the right one and then multiply the quantities by a factor 2.

```
u = Rm / 2 s (1 + Cos[Pi x / Lm]);
ux = D[u, x];
uxx = D[ux, x];
```

Total Energy and Constriction Length

Integrand of the energy of the constriction zone [Eqs. (4), (6) and (7)].

```
KCZ = (1 + ux^2 + (Rm - u ) uxx - (Rm - u) C0 (1 + ux^2) ^ (3 / 2)) ^2 / (Rm - u) / (1 + ux^2) ^ (5 / 2) +
   barp (Rm - u) ^2 + 2 barsigma (Rm - u) (1 + ux^2) ^ (1 / 2);
```

Integration between $x = 0$ and $x = L_m$ [Eq. (11)].

```
ECZ = 2 Pi k Integrate[Normal[Series[KCZ, {s, 0, 6}]], {x, 0, Lm}];
```

Minimization of the total energy in terms of L and G.

```
dECZ = D[ECZ, Lm];

Lm = (A1 + A2 s + A3 s^2) s^ (1 / 2);

dECZseries =
  Series[dECZ, {s, 0, 3}] /. barp → (L - 1 - C0^2 Rm^2 + 2 C0 Rm - 2 barsigma Rm^2) / (Rm^3) /.
   barsigma → (G - 4 + 1 - C0^2 Rm^2 + 4 C0 Rm) / (2 Rm^2);
```

```
A1sol = A1 /. Solve[Numerator[SeriesCoefficient[dECZseries, 0]] == 0, A1, Reals]
```

$$\left\{\text{ConditionalExpression}\left[-\frac{3^{1/4}\,\pi\left(\frac{Rm^4}{L}\right)^{1/4}}{2^{3/4}},\ L>0\right],\ \text{ConditionalExpression}\left[\frac{3^{1/4}\,\pi\left(\frac{Rm^4}{L}\right)^{1/4}}{2^{3/4}},\ L>0\right]\right\}$$

We take the second solution (positive length).

```
A1 = Assuming[{L > 0, Rm > 0}, Simplify[Normal@A1sol[[2]]]];
```

```
A2sol = A2 /. Solve[Numerator[SeriesCoefficient[dECZseries, 1]] == 0, A2]
```

$$\left\{\frac{\left(-12\sqrt{6}\,G-24\sqrt{L}+6\,G\sqrt{L}+12\sqrt{6}\,L-25\,L^{3/2}\right)\pi\,Rm}{192\times 6^{1/4}\,L^{5/4}}\right\}$$

```
A2 = Assuming[{L > 0, Rm > 0}, Simplify[Normal@A2sol[[1]]]];
```

```
A3sol = A3 /. Solve[Numerator[SeriesCoefficient[dECZseries, 2]] == 0, A3]
```

$$\left\{\frac{1}{36\,864\times 6^{3/4}\,L^{9/4}}\pi\,Rm\left(4320\,G^2+1728\sqrt{6}\,G\sqrt{L}-432\sqrt{6}\,G^2\sqrt{L}-30\,528\,L-5472\,G\,L+36\,G^2\,L-2880\sqrt{6}\,L^{3/2}-168\sqrt{6}\,G\,L^{3/2}+1152\,L^2+252\,G\,L^2+600\sqrt{6}\,L^{5/2}+1505\,L^3+20\,736\,C0\,L\,Rm-2304\sqrt{6}\,C0\,L^{3/2}\,Rm+864\,C0\,L^2\,Rm\right)\right\}$$

```
A3 = Assuming[{L > 0, Rm > 0}, Simplify[Normal@A3sol[[1]]]];
```

Constriction length up to third-order in s. It can be shown, after simple re-arrangement, that this expression is equivalent to the results in Eqs. (30) and (S11).

```
LmCZ = Series[Lm, {s, 0, 3}]
```

$$\frac{3^{1/4}\,\pi\,Rm\sqrt{s}}{2^{3/4}\,L^{1/4}}+\frac{\left(6\,G\left(-2\sqrt{6}+\sqrt{L}\right)-24\sqrt{L}+12\sqrt{6}\,L-25\,L^{3/2}\right)\pi\,Rm\,s^{3/2}}{192\times 6^{1/4}\,L^{5/4}}+\frac{1}{36\,864\times 6^{3/4}\,L^{9/4}}\pi\,Rm\left(36\,G^2\left(120-12\sqrt{6}\sqrt{L}+L\right)-12\,G\left(-144\sqrt{6}\sqrt{L}+456\,L+14\sqrt{6}\,L^{3/2}-21\,L^2\right)+L\left(600\sqrt{6}\,L^{3/2}+1505\,L^2+288\,L\,(4+3\,C0\,Rm)-576\sqrt{6}\sqrt{L}\,(5+4\,C0\,Rm)+576\,(-53+36\,C0\,Rm)\right)\right)s^{5/2}+O[s]^{7/2}$$

Constriction energy up to third-order in s. It can be shown, after simple re-arrangement, that this expression is equivalent to the expressions in Eqs. (31) and (S12).

```
ECZ = Series[ECZ /. barp → (L - 1 - C0^2 Rm^2 + 2 C0 Rm - 2 barsigma Rm^2) / (Rm^3) /.
    barsigma → (G - 4 + 1 - C0^2 Rm^2 + 4 C0 Rm) / (2 Rm^2), {s, 0, 3}]
```

$$\frac{4\times 2^{1/4}\,k\,L^{3/4}\,\pi^2\sqrt{s}}{3^{3/4}}-\frac{\left(k\left(-72\,6^{1/4}\,G+24\times 6^{3/4}\sqrt{L}-6\times 6^{3/4}\,G\sqrt{L}+168\times 6^{1/4}\,L+5\times 6^{3/4}\,L^{3/2}\right)\pi^2\right)s^{3/2}}{144\,L^{1/4}}-\frac{1}{27\,648\,L^{5/4}}\left(k\,\pi^2\left(864\times 6^{1/4}\,G^2+576\times 6^{3/4}\,G\sqrt{L}-144\times 6^{3/4}\,G^2\sqrt{L}-30\,528\times 6^{1/4}\,L+8352\times 6^{1/4}\,G\,L+36\times 6^{1/4}\,G^2\,L-1728\times 6^{3/4}\,L^{3/2}+1320\times 6^{3/4}\,G\,L^{3/2}-9600\times 6^{1/4}\,L^2-84\times 6^{1/4}\,G\,L^2-1080\times 6^{3/4}\,L^{5/2}-215\times 6^{1/4}\,L^3+20\,736\times 6^{1/4}\,C0\,L\,Rm+2304\times 6^{3/4}\,C0\,L^{3/2}\,Rm-288\times 6^{1/4}\,C0\,L^2\,Rm\right)\right)s^{5/2}+O[s]^{7/2}$$

Membrane Area

Membrane area of the constriction zone up to third-order in s [Eq. (22)], integration between $x = 0$ and $x = L_m$ [Eq. (11)]. It can be shown, after simple re-arrangement, that this expression is equivalent to the results in Eqs. (32) and (S14).

```
Clear[Lm]
```

$$\textbf{ACZ} = \textbf{Series}\left[4\,\textbf{Pi Integrate}\left[\textbf{Normal}\left[\textbf{Series}\left[\textbf{Rm} - \textbf{u} + \left(\frac{\textbf{Rm}}{2} - \frac{\textbf{u}}{2}\right)\textbf{ux}^2 + \left(-\frac{\textbf{Rm}}{8} + \frac{\textbf{u}}{8}\right)\textbf{ux}^4 + \left(\frac{\textbf{Rm}}{16} - \frac{\textbf{u}}{16}\right)\textbf{ux}^6, \{\textbf{s}, 0, 6\}\right]\right], \{\textbf{x}, 0, \textbf{Lm}\}\right] \;/.\; \{\textbf{Lm} \to \textbf{LmCZ}\}, \{\textbf{s}, 0, 3\}\right]$$

$$\frac{2\times 6^{1/4}\,\pi^2\,\mathrm{Rm}^2\sqrt{s}}{L^{1/4}} - \frac{\left(\pi^2\left(12\sqrt{6}\,G\,\mathrm{Rm}^2 + 24\sqrt{L}\,\mathrm{Rm}^2 - 6\,G\sqrt{L}\,\mathrm{Rm}^2 + 36\sqrt{6}\,L\,\mathrm{Rm}^2 + L^{3/2}\,\mathrm{Rm}^2\right)\right)s^{3/2}}{48\left(6^{1/4}\,L^{5/4}\right)} + \frac{1}{55\,296\,L^{9/4}}\pi^2\left(4320\times 6^{1/4}\,G^2\,\mathrm{Rm}^2 + 1728\times 6^{3/4}\,G\sqrt{L}\,\mathrm{Rm}^2 - 432\times 6^{3/4}\,G^2\sqrt{L}\,\mathrm{Rm}^2 - 30\,528\times 6^{1/4}\,L\,\mathrm{Rm}^2 + 1440\times 6^{1/4}\,G\,L\,\mathrm{Rm}^2 + 36\times 6^{1/4}\,G^2\,L\,\mathrm{Rm}^2 - 576\times 6^{3/4}\,L^{3/2}\,\mathrm{Rm}^2 - 168\times 6^{3/4}\,G\,L^{3/2}\,\mathrm{Rm}^2 - 4608\times 6^{1/4}\,L^2\,\mathrm{Rm}^2 - 36\times 6^{1/4}\,G\,L^2\,\mathrm{Rm}^2 + 120\times 6^{3/4}\,L^{5/2}\,\mathrm{Rm}^2 + 1841\times 6^{1/4}\,L^3\,\mathrm{Rm}^2 + 20\,736\times 6^{1/4}\,C0\,L\,\mathrm{Rm}^3 - 2304\times 6^{3/4}\,C0\,L^{3/2}\,\mathrm{Rm}^3 + 864\times 6^{1/4}\,C0\,L^2\,\mathrm{Rm}^3\right)s^{5/2} + O[s]^{7/2}$$

Volume enclosed

Volume enclosed by the constriction zone up to third-order in s [Eq. (23)], integration between $x = 0$ and $x = L_m$ [Eq. (11)]. It can be shown, after simple re-arrangement, that this expression is equivalent to the results in Eqs. (33) and (S15).

```
Clear[Lm]

VCZ = Series[2 Pi Integrate[u^2 - 2 Rm u + Rm^2, {x, 0, Lm}] /. {Lm → LmCZ}, {s, 0, 3}]
```

$$\frac{6^{1/4}\,\pi^2\,\mathrm{Rm}^3\sqrt{s}}{L^{1/4}} - \frac{1}{576\,L^{5/4}}\left(\left(72\times 6^{1/4}\,G + 24\times 6^{3/4}\sqrt{L} - 6\times 6^{3/4}\,G\sqrt{L} + 504\times 6^{1/4}\,L + 25\times 6^{3/4}\,L^{3/2}\right)\pi^2\,\mathrm{Rm}^3\right)s^{3/2} + \frac{1}{110\,592\,L^{9/4}}\pi^2\,\mathrm{Rm}^3\left(4320\times 6^{1/4}\,G^2 + 1728\times 6^{3/4}\,G\sqrt{L} - 432\times 6^{3/4}\,G^2\sqrt{L} - 30\,528\times 6^{1/4}\,L + 8352\times 6^{1/4}\,G\,L + 36\times 6^{1/4}\,G^2\,L + 1728\times 6^{3/4}\,L^{3/2} - 1320\times 6^{3/4}\,G\,L^{3/2} + 28\,800\times 6^{1/4}\,L^2 + 252\times 6^{1/4}\,G\,L^2 + 5400\times 6^{3/4}\,L^{5/2} + 1505\times 6^{1/4}\,L^3 + 20\,736\times 6^{1/4}\,C0\,L\,\mathrm{Rm} - 2304\times 6^{3/4}\,C0\,L^{3/2}\,\mathrm{Rm} + 864\times 6^{1/4}\,C0\,L^2\,\mathrm{Rm}\right)s^{5/2} + O[s]^{7/2}$$

Constriction Force

Constriction force up to third-order in s [Eq. (34)]. It can be shown, after simple re-arrangement, that this expression is equivalent to the results in Eqs. (35) and (S16).

```
FC = Series[Simplify[1 / Rm D[ECZ, s]], {s, 0, 6}]
```

$$\frac{2\times 2^{1/4}\,k\,L^{3/4}\,\pi^2}{3^{3/4}\,\mathrm{Rm}\sqrt{s}} - \frac{1}{16\left(6^{3/4}\,L^{1/4}\,\mathrm{Rm}\right)}\left(k\left(-6\,G\left(12 + \sqrt{6}\sqrt{L}\right) + 24\sqrt{6}\sqrt{L} + 168\,L + 5\sqrt{6}\,L^{3/2}\right)\pi^2\right)\sqrt{s} + \frac{1}{9216\times 6^{3/4}\,L^{5/4}\,\mathrm{Rm}}5\,k\,\pi^2\left(36\,G^2\left(-24 + 4\sqrt{6}\sqrt{L} - L\right) - 12\,G\left(48\sqrt{6}\sqrt{L} + 696\,L + 110\sqrt{6}\,L^{3/2} - 7\,L^2\right) + L\left(1080\sqrt{6}\,L^{3/2} + 215\,L^2 + 96\,L\,(100 + 3\,C0\,\mathrm{Rm}) - 576\sqrt{6}\sqrt{L}\,(-3 + 4\,C0\,\mathrm{Rm}) - 576\,(-53 + 36\,C0\,\mathrm{Rm})\right)\right)s^{3/2} + O[s]^{5/2}$$

References

[1] Almendro-Vedia, V.G., Monroy, F., and Cao, F.J. (2015). Analytical results for cell constriction dominated by bending energy. *Phys. Rev. E*, **91**, 012713.

[2] Almendro-Vedia, V.G., Monroy, F., and Cao, F.J. (2013). Mechanics of Constriction during Cell Division: A Variational Approach. *PLoS One*, **8**, e69750.

[3] Beltran-Heredia, E., Almendro-Vedia, V.G., Monroy, F., and Cao, F.J. (2017). Modelling the mechanics of cell division: influence of spontaneous curvature, surface tension, and osmotic pressure. *Front. Physio.*
