# Supplementary material for: Modeling the Mechanics of Cell Division: Influence of Spontaneous Membrane Curvature, Surface Tension, and Osmotic Pressure
Source: Front Physiol. 2017 May 19;8:312. doi: 10.3389/fphys.2017.00312 (PMC5437162; doi:10.3389/fphys.2017.00312)
Supplement: Supplementary file 2 [file DataSheet1.zip › Mathematica_Files/NUMERICAL.pdf]

Supplementary File: Algorithm used to compute the exact solution for symmetric constriction

These *Mathematica* and pdf files are Supplementary Files of paper

E. Beltrán-Heredia, V. G. Almendro-Vedia, F. Monroy, and F. J. Cao,

Modelling the mechanics of cell division: Influence of spontaneous membrane curvature, surface tension and osmotic pressure. (2017)

doi:10.3389/fphys.2017.00312

Below is the algorithm used to compute the exact solution for poles and constriction zone of symmetric constriction. The profile is divided in four zones: left polar cap, left half of the constriction zone, right half of the constriction zone and right polar cap.

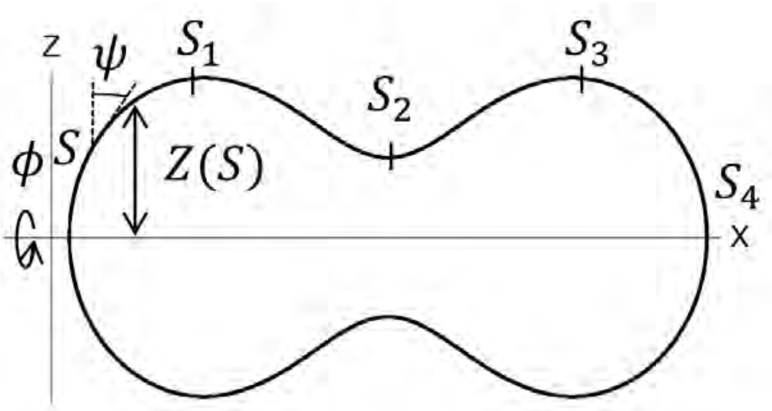

To load the functions of this notebook, execute NotebookEvaluate["C:\\Users\\Directory\\NUMERICAL.nb"] where C:\\Users\\Directory\\ has to be replaced by the directory path where the file NUMERICAL.nb is saved.

Parameters

Values for the parameters C_0, $\tilde{\Sigma}$ and $\tilde{\Delta p}$, where $\tilde{\Sigma} = \Sigma / \kappa$, and $\tilde{\Delta p} = \Delta p / \kappa$. and for the maximum radius R_m and the constriction radius R_c. For example

```
C0 = 0.3;
barp = 0;
barsigma = 0;
Rm = 1;
Rc = 0.5;
```

System

System of ordinary differential equations (Euler-Lagrange equations) [Eq. (S5)].

```
system = {psi'[S] - u[S], u'[S] + u[S] Cos[psi[S]] / z[S] - Sin[psi[S]] Cos[psi[S]] / z[S]^2 -
     gamma[S] Sin[psi[S]] / z[S] - barp z[S] / 2 Cos[psi[S]],
   gamma'[S] - (u[S] - C0)^2 / 2 + Sin[psi[S]]^2 / 2 / z[S]^2 - barp z[S] Sin[psi[S]],
   z'[S] - Cos[psi[S]], x'[S] - Sin[psi[S]]};
```

Left Polar Cap

Initial conditions

Initial conditions [Eqs. (S6)].

```
Sini = 0.001;
psiini[u0_?NumericQ] := u0 * Sini;
zini = Sini;
xini = 1 / 2 * Sini^2;
uini[u0_?NumericQ] := u0 + 1 / 4 * (barp + 4 * barsigma + u0 * (u0^2 + C0^2 + 4 * u0 * C0)) * Sini^2;
gammaini[u0_?NumericQ] := ((u0 - C0)^2 / 2 + barsigma) * Sini;
```

Solve the system from initial conditions to S_1 (see figure above).

```
caps1[u0_?NumericQ, S1_?NumericQ] :=
  NDSolve[{system[[1]] == 0, system[[2]] == 0, system[[3]] == barsigma, system[[4]] == 0,
    system[[5]] == 0, psi[Sini] == psiini[u0], u[Sini] == uini[u0], gamma[Sini] == gammaini[u0],
    x[Sini] == xini, z[Sini] == zini}, {psi, u, gamma, x, z}, {S, Sini, S1}];

end1z[u0_?NumericQ, S1_?NumericQ] := z[S1] /. caps1[u0, S1][[1]];
end1psi[u0_?NumericQ, S1_?NumericQ] := psi[S1] /. caps1[u0, S1][[1]];
```

At the end of the left pole $Z(S_1) = R_m$ and $\psi(S_1) = \pi/2$. We determine the values of U_0 and S_1 that verifiy these boundaries conditions. (Note we have to insert starting points of U_0 and S_1. These starting points may be not useful for other conditions).

```
sol1 = FindRoot[{end1z[U0, S1] == Rm, end1psi[U0, S1] == Pi / 2}, {{U0, 1}, {S1, π / 2}}]

{U0 → 1.05583, S1 → 1.65936}
```

Final values.

```
u0 = U0 /. sol1;
S1 = S1 /. sol1;
psi1 = end1psi[u0, S1];
z1 = end1z[u0, S1];
u1 = u[S1] /. caps1[u0, S1][[1]];
x1 = x[S1] /. caps1[u0, S1][[1]];
gamma1 = gamma[S1] /. caps1[u0, S1][[1]];
```

Left Half of Constriction Zone

Solve the system from the final conditions of the left polar cap to S_2 (see figure above). There is a jump in γ to maintain the maximum radius R_m fixed (see Section 1 of Supplementary Material).

```
ZC1[gammajump1_?NumericQ, S2_?NumericQ] :=
  NDSolve[{system[[1]] == 0, system[[2]] == 0, system[[3]] == barsigma, system[[4]] == 0,
    system[[5]] == 0, psi[S1] == psi1, u[S1] == u1, gamma[S1] == gammajump1, x[S1] == x1,
    z[S1] == z1}, {psi, u, gamma, x, z}, {S, S1, S2}];

end2z[gammajump1_?NumericQ, S2_?NumericQ] := z[S2] /. ZC1[gammajump1, S2][[1]];
end2psi[gammajump1_?NumericQ, S2_?NumericQ] := psi[S2] /. ZC1[gammajump1, S2][[1]];
```

At the end of the left half of constriction zone $Z(S_2) = R_c$ and $\psi(S_2) = \pi/2$. We determine the values of γ_1^+ and S_2 that verifiy these boundaries conditions. (Note we have to insert starting points of γ_1^+ and S_2. These starting points may be not useful for other conditions).

```
sol2 = FindRoot[{end2z[gammajump1, S2] == Rc, end2psi[gammajump1, S2] == Pi / 2},
  {{gammajump1, 0}, {S2, S1 + 1}}]

{gammajump1 → -0.143725, S2 → 3.30291}
```

Final values.

```
gammajump1 = gammajump1 /. sol2;
S2 = S2 /. sol2;
psi2 = end2psi[gammajump1, S2];
z2 = end2z[gammajump1, S2];
u2 = u[S2] /. ZC1[gammajump1, S2][[1]];
x2 = x[S2] /. ZC1[gammajump1, S2][[1]];
gamma2 = gamma[S2] /. ZC1[gammajump1, S2][[1]];
```

Right Half of Constriction Zone

Solve the system from the final conditions of the left half of the constriction zone to S_3 (see figure above). There is a jump in γ to maintain the constriction radius R_c fixed (see Section 1 of Supplementary Material).

```
ZC2[gammajump2_?NumericQ, S3_?NumericQ] :=
  NDSolve[{system[[1]] == 0, system[[2]] == 0, system[[3]] == barsigma, system[[4]] == 0,
    system[[5]] == 0, psi[S2] == psi2, u[S2] == u2, gamma[S2] == gammajump2, x[S2] == x2,
    z[S2] == z2}, {psi, u, gamma, x, z}, {S, S2, S3}];

end3psi[gammajump2_?NumericQ, S3_?NumericQ] := psi[S3] /. ZC2[gammajump2, S3][[1]];
end3z[gammajump2_?NumericQ, S3_?NumericQ] := z[S3] /. ZC2[gammajump2, S3][[1]];
```

At the end of the right half of constriction zone $Z(S_3) = R_m$ and $\psi(S_3) = \pi/2$. We determine the values of γ_2^+ and S_3 that verifiy these boundaries conditions. (Note we have to insert starting points of γ_2^+ and S_3 . These starting points may be not useful for other conditions).

```
sol3 = FindRoot[{end3z[gammajump2, S3] == Rm, end3psi[gammajump2, S3] == Pi / 2},
  {{gammajump2, 1}, {S3, S2 + 1}}]

{gammajump2 → 1.09913, S3 → 4.94646}

gammajump2 = gammajump2 /. sol3;
S3 = S3 /. sol3;
psi3 = end3psi[gammajump2, S3];
z3 = end3z[gammajump2, S3];
u3 = u[S3] /. ZC2[gammajump2, S3][[1]];
x3 = x[S3] /. ZC2[gammajump2, S3][[1]];
gamma3 = gamma[S3] /. ZC2[gammajump2, S3][[1]];
```

Right Polar Cap

Solve the system from the final conditons of the right half of the constriction zone to S_4 (see figure above). There is a jump in γ to maintain the maximum radius R_m fixed (see Section 1 of Supplementary Material).

```
caps2[gammajump3_?NumericQ, S4_?NumericQ] :=
  NDSolve[{system[[1]] == 0, system[[2]] == 0, system[[3]] == barsigma, system[[4]] == 0,
    system[[5]] == 0, psi[S3] == psi3, u[S3] == u3, gamma[S3] == gammajump3, x[S3] == x3,
    z[S3] == z3}, {psi, u, gamma, x, z}, {S, S3, S4}];

end4psi[gammajump3_?NumericQ, S4_?NumericQ] := psi[S4] /. caps2[gammajump3, S4][[1]];
end4u[gammajump3_?NumericQ, S4_?NumericQ] := u[S4] /. caps2[gammajump3, S4][[1]];
end4z[gammajump3_?NumericQ, S4_?NumericQ] := z[S4] /. caps2[gammajump3, S4][[1]];
```

At the end of the right half of constriction zone $Z(S_4) + S_{\text{ini}} = 0$ and $\psi\,(S_4) + S_{\text{ini}}\,U(S_4) = \pi$ (since $Z(S_4) = 0$ is a singular point of the system, we use the Taylor expansion around $S = S_4$). We determine the values of γ_3^+ and S_4 that verifiy these boundaries conditions. (Note we have to insert starting points of γ_3^+ and S_4. These starting points may be not useful for other conditions).

```
sol4 =
 FindRoot[{end4z[gammajump3, S4] - Sini == 0,
   end4psi[gammajump3, S4] + Sini * end4u[gammajump3, S4] == Pi},
  {{gammajump3, 0}, {S4, S3 + 1}}]

{gammajump3 → 0.525854, S4 → 6.60481}
```

```
gammajump3 = gammajump3 /. sol4;
S4 = S4 /. sol4;
psi4 = end4psi[gammajump3, S4];
z4 = end4z[gammajump3, S4];
u4 = end4u[gammajump3, S4];
x4 = x[S4] /. caps2[gammajump3, S4][[1]];
gamma4 = gamma[S4] /. caps2[gammajump3, S4][[1]];
```

Calculation of the variables Z, X, U, ψ

We calculate the variables Z, X, U, ψ from S_1 to S_4.

```
fXcaps1[S] = x[S] /. caps1[u0, S1][[1]];
fZcaps1[S] = z[S] /. caps1[u0, S1][[1]];
fUcaps1[S] = u[S] /. caps1[u0, S1][[1]];
fpsicaps1[S] = psi[S] /. caps1[u0, S1][[1]];

fXZC1[S] = x[S] /. ZC1[gammajump1, S2][[1]];
fZZC1[S] = z[S] /. ZC1[gammajump1, S2][[1]];
fUZC1[S] = u[S] /. ZC1[gammajump1, S2][[1]];
fpsiZC1[S] = psi[S] /. ZC1[gammajump1, S2][[1]];

fXZC2[S] = x[S] /. ZC2[gammajump2, S3][[1]];
fZZC2[S] = z[S] /. ZC2[gammajump2, S3][[1]];
fUZC2[S] = u[S] /. ZC2[gammajump2, S3][[1]];
fpsiZC2[S] = psi[S] /. ZC2[gammajump2, S3][[1]];

fXcaps2[S] = x[S] /. caps2[gammajump3, S4][[1]];
fZcaps2[S] = z[S] /. caps2[gammajump3, S4][[1]];
fUcaps2[S] = u[S] /. caps2[gammajump3, S4][[1]];
fpsicaps2[S] = psi[S] /. caps2[gammajump3, S4][[1]];
```

Profile of the solution

We plot the profile of the solution.

```
grafcaps1 = Table[Evaluate[{fXcaps1[S], fZcaps1[S]}], {S, Sini, S1, (S1 - Sini) / 5000}];
grafZC1 = Table[Evaluate[{fXZC1[S], fZZC1[S]}], {S, S1, S2, (S2 - S1) / 5000}];
grafZC2 = Table[Evaluate[{fXZC2[S], fZZC2[S]}], {S, S2, S3, (S3 - S2) / 5000}];
grafcaps2 = Table[Evaluate[{fXcaps2[S], fZcaps2[S]}], {S, S3, S4, (S4 - S3) / 5000}];

invgrafcaps1 = Table[Evaluate[{fXcaps1[S], -fZcaps1[S]}], {S, Sini, S1, (S1 - Sini) / 5000}];
invgrafZC1 = Table[Evaluate[{fXZC1[S], -fZZC1[S]}], {S, S1, S2, (S2 - S1) / 5000}];
invgrafZC2 = Table[Evaluate[{fXZC2[S], -fZZC2[S]}], {S, S2, S3, (S3 - S2) / 5000}];
invgrafcaps2 = Table[Evaluate[{fXcaps2[S], -fZcaps2[S]}], {S, S3, S4, (S4 - S3) / 5000}];

profile = Join[grafcaps1, invgrafcaps1, grafZC1, invgrafZC1, grafZC2, invgrafZC2,
   grafcaps2, invgrafcaps2];
```

```
ListPlot[{profile}, ImageSize → 560, AxesLabel → {"X", "Z=R(x)"},
 LabelStyle → Directive[FontSize → 18, FontFamily → "Helvetica"], AspectRatio → 0.5]
```

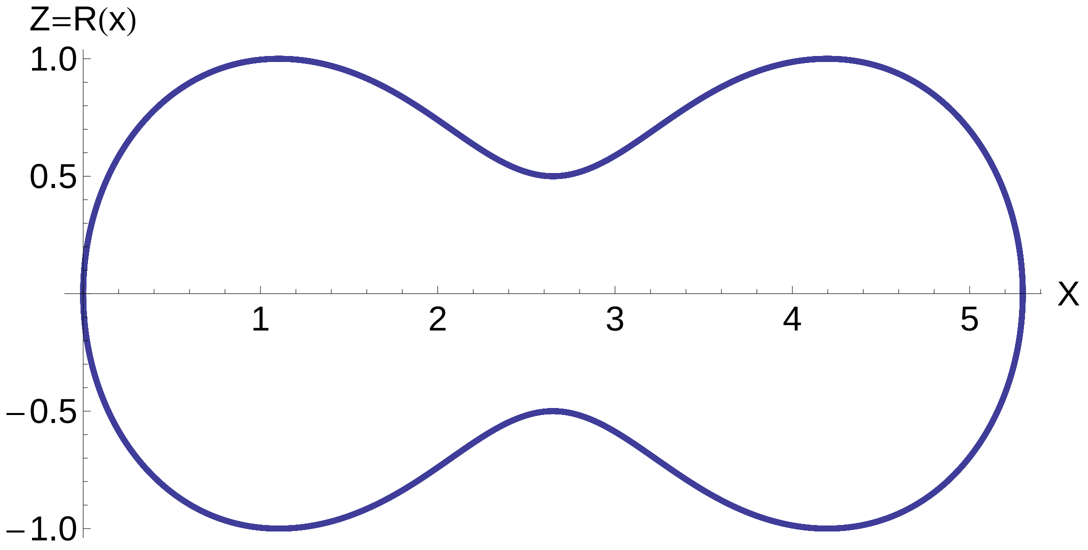

Membrane Area

Membrane area [Eq. (S8)].

```
Acaps1 = NIntegrate[fZcaps1[S] * 2 * Pi, {S, Sini, S1}];
AZC1 = NIntegrate[fZZC1[S] * 2 * Pi, {S, S1, S2}];
Acaps2 = NIntegrate[fZcaps2[S] * 2 * Pi, {S, S3, S4}];
AZC2 = NIntegrate[fZZC2[S] * 2 * Pi, {S, S2, S3}];
AT = Acaps1 + Acaps2 + AZC1 + AZC2;

AT / (4 Pi Rm^2)

2.35132
```

Volume enclosed

Volume enclosed [Eq. (S9)].

```
Vcaps1 = NIntegrate[fZcaps1[S]^2 * Pi * Sin[fpsicaps1[S]], {S, Sini, S1}];
VZC1 = NIntegrate[fZZC1[S]^2 * Pi * Sin[fpsiZC1[S]], {S, S1, S2}];
Vcaps2 = NIntegrate[fZcaps2[S]^2 * Pi * Sin[fpsicaps2[S]], {S, S3, S4}];
VZC2 = NIntegrate[fZZC2[S]^2 * Pi * Sin[fpsiZC2[S]], {S, S2, S3}];
VT = Vcaps1 + Vcaps2 + VZC1 + VZC2;

VT / (4 / 3 Pi Rm^3)

2.58763
```

Total Energy

Total energy [Eqs. (S3) and (S4)].

```
ETcaps1 =
  k NIntegrate[fZcaps1[S] Pi (fUcaps1[S] + Sin[fpsicaps1[S]] / fZcaps1[S] - C0) ^2 +
     barsigma Acaps1 + barp Vcaps1, {S, Sini, S1}];
ETZC1 =
  k NIntegrate[fZZC1[S] Pi (fUZC1[S] + Sin[fpsiZC1[S]] / fZZC1[S] - C0) ^2 + barsigma AZC1 +
     barp VZC1, {S, S1, S2}];
ETZC2 =
  k NIntegrate[fZZC2[S] Pi (fUZC2[S] + Sin[fpsiZC2[S]] / fZZC2[S] - C0) ^2 +
     barsigma AZC2 + barp VZC2, {S, S2, S3}];
ETcaps2 =
  k NIntegrate[fZcaps2[S] Pi (fUcaps2[S] + Sin[fpsicaps2[S]] / fZcaps2[S] - C0) ^2 +
     barsigma Acaps2 + barp Vcaps2, {S, S3, S4}];
ET = ETcaps1 + ETcaps2 + ETZC1 + ETZC2;
```

```
ET / (8 Pi k)
```

```
1.11791
```

Polar Distance

```
Lp = Evaluate[fXcaps1[S] /. S → S1];
```

```
Lp / (Rm)
```

```
1.10327
```

Constriction Length

```
Lm = Evaluate[fXZC1[S] /. S → S2] - Evaluate[fXcaps1[S] /. S → S1];
```

```
Lm / (Rm)
```

```
1.54674
```

Line tensions and Constriction Force

Line tension σ_c [Eq. (S7b)].

```
sigmac = gammajump2 - gamma2
```

```
2.19826
```

Line tension σ_m [Eq. (S7a)].

```
sigmam = gammajump1 - gamma1
```

```
0.382128
```

Line tension $\sigma_{\mathrm{m}}^{'}$ [Eq. (S7a)].

```
sigmamprima = gammajump3 - gamma3
```

```
0.382129
```

Force exerted at the maximum radius sites toward the exterior [Eq. (S20b)].

```
Fm = 4 * Pi * sigmam
```

```
4.80197
```

Force exerted at the constriction site toward the interior [Eq. (S20a)].

```
Fc = 2 * Pi * sigmac
```

```
13.8121
```

References

[1] Almendro-Vedia, V.G., Monroy, F., and Cao, F.J. (2015). Analytical results for cell constriction dominated by bending energy. *Phys. Rev. E,* **91**, 012713.

[2] Almendro-Vedia, V.G., Monroy, F., and Cao, F.J. (2013). Mechanics of Constriction during Cell Division: A Variational Approach. *PLoS One,* **8**, e69750.

[3] Beltran-Heredia, E., Almendro-Vedia, V.G., Monroy, F., and Cao, F.J. (2017). Modelling the mechanics of cell division: influence of spontaneous curvature, surface tension, and osmotic pressure. *Front. Physio.*

[4] Seifert, U., Berndl K., and Lipowsky, R. (1991). Shape transformations of vesicles: Phase diagram for spontaneous-curvature and bilayer-coupling models, *Phys. Rev. A*. **44**, 1182-1202.

[5] Jülicher, F., Lipowsky, R. (1996). Shape transformations of vesicles with intramembrane domains. *Phys. Rev. E.* **53**, 2670-2683.
